# Supplementary material for: Traffic Noise and Mental Health: A Systematic Review and Meta-Analysis
Source: Int J Environ Res Public Health. 2020 Aug 25;17(17):6175. doi: 10.3390/ijerph17176175 (PMC7503511; doi:10.3390/ijerph17176175)
Supplement: Supplementary file 1 [file ijerph-17-06175-s001.pdf]

International Journal of
Environmental Research and Public Health

MDPI

1. Data Extraction

Table 1. Data Extraction.

<table>
<tr><th rowspan="2">Reference (First author, publication year, S/N) Meta-analysis</th><th rowspan="2">Study design</th><th>Population</th><th>Outcome</th><th colspan="3">Exposure</th><th rowspan="2">Results</th><th rowspan="2">Comments (study quality [overall assessment according to SIGN/CASP], conflict of interest [stated vs. not stated], funding [financed from public funds vs. financed from industry], confounding, strengths / weaknesses [potential bias, over- or underestimation of potential effects])</th></tr>
<tr><th>Study region
Sample population
Sample size (M, F, M+F):
Age (mean, range)
No. of cases / no. of controls or exposed/unexposed
Time of recruitment / follow-up (mean, range)
Response (%)
(baseline minus loss to follow-up)</th><th>Disease (ICD-10)
Prescription
Questionnaire</th><th>exposure source</th><th>exposure assessment</th><th>exposure levels</th></tr>
<tr><td>Andersson, 2018, 8552</td><td>Cohort study

(Betula project)</td><td>Study region:
Umeå municipality, Northern Sweden

Sample size:
M+F=1,721
M= 985, F= 736
Random selection of participants

Sample population:</td><td>Dementia diagnosed through 3-phase procedure: 1. Health examination with neuropsychological testing, structured interviews and observations</td><td>Road traffic noise

Data from 2012

Assessment of NO_x (for 2009-2010)</td><td>$L_{eq,24h}$</td><td>< 55 dB
≥ 55 dB</td><td>Table 2: HR (Model 3)<table><tr><th>dB</th><th>N</th><th>HR</th><th>95% CI</th></tr><tr><td>< 55</td><td>1,619</td><td></td><td></td></tr><tr><td>≥ 55</td><td>102</td><td>0.95</td><td>0.57-1.57</td></tr></table>(not adjusted for air pollution)</td><td>study quality:
-
Cohort study
conflict of interest:
stated (none declared)
funding:
stated: Swedish Research Council Formas, Stockholm, Sweden [registration number</td></tr>
</table>

Reference (First author, publication year, S/N) Meta-analysis	Study design	Population: Study region, Sample population, Sample size (M, F, M+F): Age (mean, range), No. of cases / no. of controls or exposed/unexposed, Time of recruitment / follow-up (mean, range), Response (%) (baseline minus loss to follow-up)	Outcome: Disease (ICD-10), Prescription, Questionnaire	Exposure: exposure source	Exposure: exposure assessment	Exposure: exposure levels	Results	Comments (study quality [overall assessment according to SIGN/CASP], conflict of interest [stated vs. not stated], funding [financed from public funds vs. financed from industry], confounding, strengths / weaknesses [potential bias, over- or underestimation of potential effects])
		Inclusion criteria: ≥ 55 years at baseline,<br>Exclusion criteria: any subtype of dementia that was not vascular dementia or Alzheimer's disease, dead, lost to follow-up<br><br>Age:<br>Mean 68.5 ± 9.4 years (55-85 years)<br><br>Time of recruitment:<br>1993-2010<br><br>Response:	2. in case of a suspected case of dementia→ examination by specialist in geriatric medicine or geriatric psychiatry, careful review of each individual´s medical record<br>3. specialist in geriatric psychiatry or in geriatric					942–2015-1099]<br>confounding (controlled for): baseline age, education, physical activity, smoking, sex, body mass index, waist–hip ratio, alcohol, ApoE4, baseline medical history of diabetes, hypertension, and stroke.<br>strengths, weaknesses:<br>- Exposure data

Reference (First author, publication year, S/N) Meta-analysis	Study design	Population<br>Study region<br>Sample population<br>Sample size (M, F, M+F):<br>Age (mean, range)<br>No. of cases / no. of controls or exposed/unexposed<br>Time of recruitment / follow-up (mean, range)<br>Response (%)<br>(baseline minus loss to follow-up)	Outcome<br>Disease (ICD-10)<br>Prescription<br>Questionnaire	Exposure<br>exposure source	exposure assessment	exposure levels	Results	Comments (study quality [overall assessment according to SIGN/CASP], conflict of interest [stated vs. not stated], funding [financed from public funds vs. financed from industry], confounding, strengths / weaknesses [potential bias, over- or underestimation of potential effects])
		Not reported (also checked Oudin et al. 2016, Boraxberkk et al. 2015)	medicine made last decision of the diagnosis					collected after outcome (chronology)<br>- No information on response<br>- Exclusion of lost follow-up<br>- No adjustment of air pollution<br>+cohort study-> hazard ratios<br>+ random selection of participants<br>+ adequate definition and assessment of outcome<br>+adequate control for

Reference (First author, publication year, S/N) Meta-analysis	Study design	Population<br>Study region<br>Sample population<br>Sample size (M, F, M+F):<br>Age (mean, range)<br>No. of cases / no. of controls or exposed/unexposed<br>Time of recruitment / follow-up (mean, range)<br>Response (%)<br>(baseline minus loss to follow-up)	Outcome<br>Disease (ICD-10)<br>Prescription<br>Questionnaire	Exposure<br>exposure source	exposure assessment	exposure levels	Results	Comments (study quality [overall assessment according to SIGN/CASP], conflict of interest [stated vs. not stated], funding [financed from public funds vs. financed from industry], confounding, strengths / weaknesses [potential bias, over- or underestimation of potential effects])
								confounders<br>+ adequate exposure assessment<br>+ ethics approval by Regional Ethics Review Board at Umeå University
Baudin, C.2018, 8456	Cross-sectional	Study region<br>Population >18 living Around 3 airports in France (Paris-Charles de Gaulle, Lyon-Saint-Exupery and Toulouse-Blagnac)<br><br>Sample size (participated)	Face-to-face interview at home<br>Psychological ill health:<br>GHQ-12	Aircraft	L_{DEN}<br>$L_{eq,24h}$<br>$L_{eq,16h}$ (6-22h)<br>L_N (22-06h)	<40,<br>50–54,<br>55–59,<br>≥60 dB	Per 10 dB increase<br><br>L_{DEN}: OR = 1.02 (0.78-1.34)<br><br>L_{DEN}: OR = 0.93 (0.69-1.24) – additionally adjusted for noise sensitivity and annoyance	study quality:<br>-(to+)<br>Cross-sectional study design<br>conflict of interest:<br>stated<br>funding:<br>stated

Reference (First author, publication year, S/N) Meta-analysis	Study design	Population: Study region Sample population Sample size (M, F, M+F): Age (mean, range) No. of cases / no. of controls or exposed/unexposed Time of recruitment / follow-up (mean, range) Response (%) (baseline minus loss to follow-up)	Outcome: Disease (ICD-10) Prescription Questionnaire	Exposure: exposure source	exposure assessment	exposure levels	Results	Comments (study quality [overall assessment according to SIGN/CASP], conflict of interest [stated vs. not stated], funding [financed from public funds vs. financed from industry], confounding, strengths / weaknesses [potential bias, over- or underestimation of potential effects])
		M = 549, F = 695<br>M+F 1,244<br><br>Age<br>18-34y: 226<br>35-44y: 236<br>45-54y: 266<br>55-64y: 260<br>65-74y: 185<br>≥ 75y: 71<br>Sample size (analyzed)<br>M = 534, F = 688<br>M+F 1,222<br><br>Time of recruitment<br>2013					(only results for L_{DEN} presented -> reason: similar results)	confounding (controlled for): sex, age, country of birth, occupational activity, education, marital status, smoking, alcohol consumption, number of work-related stress and major stressful life events, monthly household income, sleep duration, antidepressant use, self-reported anxiety strengths,

Reference (First author, publication year, S/N) Meta-analysis	Study design	Population: Study region Sample population Sample size (M, F, M+F): Age (mean, range) No. of cases / no. of controls or exposed/unexposed Time of recruitment / follow-up (mean, range) Response (%) (baseline minus loss to follow-up)	Outcome: Disease (ICD-10) Prescription Questionnaire	Exposure: exposure source	Exposure: exposure assessment	Exposure: exposure levels	Results	Comments (study quality [overall assessment according to SIGN/CASP], conflict of interest [stated vs. not stated], funding [financed from public funds vs. financed from industry], confounding, strengths / weaknesses [potential bias, over- or underestimation of potential effects])
		Response rate (%) 30% (N=4,202, responders: n = 1,244) 40% of non-participants answered a short questionnaire -> Characteristics of participants and non-participants presented descriptively.-> slight differences in marital status, socio-occupation						weaknesses: -Cross-sectional study, no information about time of exposure assessment - response only 30% + random selection of households from a phone directory, then random selection of a respondent within the household + Non-responder analysis (but slight differences in regard to marital status or

Reference (First author, publication year, S/N) Meta-analysis	Study design	Population: Study region Sample population Sample size (M, F, M+F): Age (mean, range) No. of cases / no. of controls or exposed/unexposed Time of recruitment / follow-up (mean, range) Response (%) (baseline minus loss to follow-up)	Outcome: Disease (ICD-10) Prescription Questionnaire	Exposure: exposure source	Exposure: exposure assessment	Exposure: exposure levels	Results	Comments (study quality [overall assessment according to SIGN/CASP], conflict of interest [stated vs. not stated], funding [financed from public funds vs. financed from industry], confounding, strengths / weaknesses [potential bias, over- or underestimation of potential effects])
								occupation)<br>+ adequate definition and assessment of outcome<br>+ adequate exposure assessment<br>+ high statistical power<br>adequate control for confounders (sensitivity analyses adjusting for noise sensitivity and annoyance)<br>+ ethics approval by 2 national authorities in

Reference (First author, publication year, S/N) Meta-analysis	Study design	Population<br>Study region<br>Sample population<br>Sample size (M, F, M+F):<br>Age (mean, range)<br>No. of cases / no. of controls or exposed/unexposed<br>Time of recruitment / follow-up (mean, range)<br>Response (%)<br>(baseline minus loss to follow-up)	Outcome<br>Disease (ICD-10)<br>Prescription<br>Questionnaire	Exposure<br>exposure source	exposure assessment	exposure levels	Results	Comments (study quality [overall assessment according to SIGN/CASP], conflict of interest [stated vs. not stated], funding [financed from public funds vs. financed from industry], confounding, strengths / weaknesses [potential bias, over- or underestimation of potential effects])
								France (French Advisory Committee for Data Processing in Health Research; French National Commission for Data Protection and the Liberties)
Bocquier, A. 2014, 300, France Yes	Retro prospective cohort study	Study region: Marseilles, France<br><br>Sample size: M+F=190,617<br>M= 87,975, F=102,642<br><br>Sample population:	Prescriptions total number of purchases of anxiolytics–hypnotics (N05B-Anxiolytics, N05CD-Benzodia	Road traffic	L_N (22-06h)<br><br>Environmental Noise Directive (END) 2002/49/EC, using the CadnaA	L_N<br>< 45dB (Ref)<br>45-50 dB,<br>50-55 dB,<br>≥ 55 dB	Anxiolytic-hypnotic purchase in 2008-9 (from Table 3)<br><br>Low deprivation (n=41,054)<br>L_N (dB) / RR (95%CI) / n<br>< 45 / Ref / 16,289<br>45-50 / 0.96 / 15,267	study quality: - (to +)<br><br>conflict of interest: not stated<br><br>funding: Agence

		Population	Outcome	Exposure				
**Reference (First author, publication year, S/N) Meta-analysis**	**Study design**	**Study region<br>Sample population<br>Sample size (M, F, M+F):<br>Age (mean, range)<br>No. of cases / no. of controls or exposed/unexposed<br>Time of recruitment / follow-up (mean, range)<br>Response (%)<br>(baseline minus loss to follow-up)**	**Disease (ICD-10)<br>Prescription<br>Questionnaire**	**exposure source**	**exposure assessment**	**exposure levels**	**Results**	**Comments<br>(study quality [overall assessment according to SIGN/CASP], conflict of interest [stated vs. not stated], funding [financed from public funds vs. financed from industry], confounding, strengths / weaknesses [potential bias, over- or underestimation of potential effects])**
		Those registered at French National Health Insurance Fund in SE France<br><br>Age:<br>41.5 years (SD 12.5)<br><br>No of cases/ no of controls:<br>See results<br><br>Time of recruitment/<br>follow-up: 2008-9<br><br>Response:<br>47% baseline participation	zepine derivates and N05CF-Benzodiazepine related drugs)		environmental noise prediction model<br><br>Measurement in 2006		(0.91-1.01)<br>50-55 1.04 (0.97-1.11) 8,075<br>≥ 55 1.16 (1.01-1.32) 1,423<br><br>Intermediate deprivation (n=102,284)<br>L_N (dB) RR (95%CI) n<br>< 45 Ref 17,074<br>45-50 1.02 (0.97-1.07) 31,800<br>50-55 0.99 (0.94-1.03) 40,013<br>≥ 55 0.96 (0.91-1.02) 13,397	de l'environnement et de la maîtrise de l'énergie (Ademe) + Groupement d'intérêt scientifique Institut de recherche en santé publique (GIS-IReSP) (French Environment & Energy Management Agency + Institute for Public Health Research)<br><br>confounding (adjusted for):<br>age, gender, chronic

Reference (First author, publication year, S/N) Meta-analysis	Study design	Population: Study region Sample population Sample size (M, F, M+F): Age (mean, range) No. of cases / no. of controls or exposed/unexposed Time of recruitment / follow-up (mean, range) Response (%) (baseline minus loss to follow-up)	Outcome: Disease (ICD-10) Prescription Questionnaire	Exposure: exposure source	Exposure: exposure assessment	Exposure: exposure levels	Results	Comments (study quality [overall assessment according to SIGN/CASP], conflict of interest [stated vs. not stated], funding [financed from public funds vs. financed from industry], confounding, strengths / weaknesses [potential bias, over- or underestimation of potential effects])
							High deprivation (n=47.279)<br>L_N (dB) / RR 95%CI / n<br>< 45 / Ref / 7,342<br>45-50 / 1.00 (0.93-1.08) / 13,190<br>50-55 / 1.02 (0.94-1.10) / 19,015<br>≥ 55d / 0.95 (0.86-1.04) / 7732<br><br>Deprivation index was calculated by 17 questions concerning socio-economic state (census block)	somatic disease, severe psychiatric disorder, CMUC coverage as a proxy for low income, number of consultations in 2008–9 with GPs, characteristics of the linked physicians and their clientele, density of GPs and of psychiatrists, and number of complaints filed for noise problems other

Reference (First author, publication year, S/N) Meta-analysis	Study design	Population: Study region Sample population Sample size (M, F, M+F): Age (mean, range) No. of cases / no. of controls or exposed/unexposed Time of recruitment / follow-up (mean, range) Response (%) (baseline minus loss to follow-up)	Outcome: Disease (ICD-10) Prescription Questionnaire	Exposure: exposure source	Exposure: exposure assessment	Exposure: exposure levels	Results	Comments (study quality [overall assessment according to SIGN/CASP], conflict of interest [stated vs. not stated], funding [financed from public funds vs. financed from industry], confounding, strengths / weaknesses [potential bias, over- or underestimation of potential effects])
								than traffic noise in each individual's census block<br><br>strengths/ weaknesses:<br>+ cohort<br>+ adequate exposure assessment<br>+ high number of cases & controls<br>+ objective measurement (prescriptions)<br>+ 47% baseline participation (53%

Reference (First author, publication year, S/N) Meta-analysis	Study design	Population: Study region Sample population Sample size (M, F, M+F): Age (mean, range) No. of cases / no. of controls or exposed/unexposed Time of recruitment / follow-up (mean, range) Response (%) (baseline minus loss to follow-up)	Outcome: Disease (ICD-10) Prescription Questionnaire	Exposure: exposure source	Exposure: exposure assessment	Exposure: exposure levels	Results	Comments (study quality [overall assessment according to SIGN/CASP], conflict of interest [stated vs. not stated], funding [financed from public funds vs. financed from industry], confounding, strengths / weaknesses [potential bias, over- or underestimation of potential effects])
								could not be geo-located), low lost-to-follow-up might be expected (although not measureable)<br>- limited differentiation between incident and prevalent cases<br>- outcome medication does not allow for separation of anxiolytics and hypnotics<br>- potential over

Reference (First author, publication year, S/N) Meta-analysis	Study design	Population: Study region Sample population Sample size (M, F, M+F): Age (mean, range) No. of cases / no. of controls or exposed/unexposed Time of recruitment / follow-up (mean, range) Response (%) (baseline minus loss to follow-up)	Outcome: Disease (ICD-10) Prescription Questionnaire	Exposure: exposure source	Exposure: exposure assessment	Exposure: exposure levels	Results	Comments (study quality [overall assessment according to SIGN/CASP], conflict of interest [stated vs. not stated], funding [financed from public funds vs. financed from industry], confounding, strengths / weaknesses [potential bias, over- or underestimation of potential effects])
								adjustment for number of consultations with GPs<br>- no other source of noise considered<br>- other confounders, such as air quality not considered
Carey, 2018, 8494	Retrospective Cohort stuy	Study region:<br>Greater London (75 practices): study area bounded by M25 motorway around Greater London: 60 practices in outer London boroughs, 15 inner London boroughs	Primary care database (Clinical Practice Research Datalink) -> first dementia diagnosis using Read codes for	Road traffic noise<br><br>Estimated at postcode	L_N (23:00-07:00) ($L_{eq,16h}$ produced identical data)	no categories	Incident dementia: Table 3<br>HR = 1.02 (95% CI 1.00-1.05)<br><br>Sensitivity analysis with NO_2:<br>HR = 1.01 (95% CI 0.98-1.03)	study quality:<br>+<br>Cohort study<br>conflict of interest:<br>stated (none declared)<br>funding:<br>stated (UK Natural

<table>
<tr><th rowspan="2">Reference (First author, publication year, S/N) Meta-analysis</th><th rowspan="2">Study design</th><th>Population</th><th>Outcome</th><th colspan="3">Exposure</th><th rowspan="2">Results</th><th rowspan="2">Comments (study quality [overall assessment according to SIGN/CASP], conflict of interest [stated vs. not stated], funding [financed from public funds vs. financed from industry], confounding, strengths / weaknesses [potential bias, over- or underestimation of potential effects])</th></tr>
<tr><th>Study region
Sample population
Sample size (M, F, M+F):
Age (mean, range)
No. of cases / no. of controls or exposed/unexposed
Time of recruitment / follow-up (mean, range)
Response (%)
(baseline minus loss to follow-up)</th><th>Disease (ICD-10)
Prescription
Questionnaire</th><th>exposure source</th><th>exposure assessment</th><th>exposure levels</th></tr>
<tr><td></td><td></td><td>Sample size:
(N=555,385 patients registered (7% of Greater London population)
M+F= 130,978
M= 65,130, F= 65,848

Sample population:
> only patients between 50-79 years included
->exclusion: existing dementia diagnosis by 1 January 2005 (n=391), living in care home (n=423), no Index of Multiple Deprivation (IMD) (n=77))</td><td>dementia</td><td>level

Data estimated annually 2004-2010</td><td></td><td></td><td>(sensitivity analysis performed with classifying dementia as Alzheimer's disease, vascular dementia or non-specific)
Alzheimer´s disease:<table><tr><th>dB</th><th>HR</th><th>95% CI</th></tr><tr><td>0-49.4</td><td>1</td><td></td></tr><tr><td>>49.4-49.6</td><td>0.95</td><td>(0.76–1.18)</td></tr><tr><td>>49.6-50.3</td><td>0.96</td><td>(0.77–1.20)</td></tr><tr><td>>50.3-53.8</td><td>0.94</td><td>(0.75–1.18)</td></tr><tr><td>≥53.8</td><td>1.05</td><td>(0.84–1.31)</td></tr><tr><td>+2,7 (IQR change)</td><td>1.03</td><td>(0.99–1.07)</td></tr></table></td><td>Environment Research Council, Medical Research Council, Economic and Social Research Counciel, Department for Environment, Food and Rural Affairs, and Department of Health through Environmental Exposure & Health Initiative. Partly by National Institute for Environmental</td></tr>
</table>

Reference (First author, publication year, S/N) Meta-analysis	Study design	Population: Study region Sample population Sample size (M, F, M+F): Age (mean, range) No. of cases / no. of controls or exposed/unexposed Time of recruitment / follow-up (mean, range) Response (%) (baseline minus loss to follow-up)	Outcome: Disease (ICD-10) Prescription Questionnaire	Exposure: exposure source	Exposure: exposure assessment	Exposure: exposure levels	Results	Comments (study quality [overall assessment according to SIGN/CASP], conflict of interest [stated vs. not stated], funding [financed from public funds vs. financed from industry], confounding, strengths / weaknesses [potential bias, over- or underestimation of potential effects])
		Age:<br>50-59 years: n=59,587<br>60-69 years: n=41,013<br>70-79 years: n=30,378<br><br>Time of recruitment:<br>2005-2013<br><br>Response:<br>-not applicable: secondary data<br>Follow-up period: mean=6.9 years					Vascular dementia:<br>dB / HR / 95% CI<br>0-49.4 / 1 /<br>>49.4-49.6 / 1.22 / (0.94–1.58)<br>>49.6-50.3 / 1.23 / (0.94–1.59)<br>>50.3-53.8 / 1.17 / (0.90–1.52)<br>≥53.8 / 1.09 / (0.83–1.42)<br>+2,7 (IQR change) / 1.00 / (0.96–1.05)<br><br>Non-specific:<br>dB / HR / 95% CI<br>0-49.4 / 1 /	Hazards at King`s College andon in partnership with Public Health England and Imperial College London.<br>confounding (controlled for): age, sex, ethnicity, smoking and body mass index, Index of Multiple Deprivation, ischaemic heart disease, stroke, diabetes, heart failure, NO2

Reference (First author, publication year, S/N) Meta-analysis	Study design	Population: Study region<br>Sample population<br>Sample size (M, F, M+F):<br>Age (mean, range)<br>No. of cases / no. of controls or exposed/unexposed<br>Time of recruitment / follow-up (mean, range)<br>Response (%)<br>(baseline minus loss to follow-up)	Outcome: Disease (ICD-10)<br>Prescription<br>Questionnaire	Exposure: exposure source	Exposure: exposure assessment	Exposure: exposure levels	Results			Comments<br>(study quality [overall assessment according to SIGN/CASP], conflict of interest [stated vs. not stated], funding [financed from public funds vs. financed from industry], confounding, strengths / weaknesses [potential bias, over- or underestimation of potential effects])
							>49.4-49.6	1.07	(0.85–1.34)	strengths, weaknesses:<br>+cohort study-> hazard ratios<br>+ adequate definition and assessment of outcome<br>+adequate control for confounders+ adjustment of NO_2<br>+ adequate exposure assessment<br>+ high statistical power<br>+ ethics approval by Independent Scientific
							>49.6-50.3	0.97	(0.77–1.23)	
							>50.3-53.8	0.93	(0.73–1.19)	
							≥53.8	1.14	(0.91–1.43)	
							+2,7 (IQR change)	1.03	(0.99–1.07)	

Reference (First author, publication year, S/N) Meta-analysis	Study design	Population: Study region, Sample population, Sample size (M, F, M+F): Age (mean, range), No. of cases / no. of controls or exposed/unexposed, Time of recruitment / follow-up (mean, range), Response (%) (baseline minus loss to follow-up)	Outcome: Disease (ICD-10), Prescription, Questionnaire	Exposure: exposure source	Exposure: exposure assessment	Exposure: exposure levels	Results	Comments (study quality [overall assessment according to SIGN/CASP], conflict of interest [stated vs. not stated], funding [financed from public funds vs. financed from industry], confounding, strengths / weaknesses [potential bias, over- or underestimation of potential effects])
								Advisory Committee
Floud, S. 2011, 751, Europe<br><br>HYENA Study Yes	Cross-sectional study	Study region:<br>London Heathrow (UK), Amsterdam Schiphol (The Netherlands), Stockholm Arlanda & Bromma (Sweden), Milan Malpensa (Italy), Berlin Tegel (Germany), Athen Elephtherios Venizelos (Greece) airports<br><br>Study population:<br>randomly selected sample (stratified random sampling	- self-reported prescribed medication two weeks preceding the interview<br>- coded according to the ATC classification system: anxiolytics, hypnotics, antidepressants	Aircraft, Road traffic	Aircraft noise (2002):<br>$L_{eq,16h}$<br>(07-23h or 06-22h)<br><br>L_N<br>(23-07h or 22-06h)<br><br>Integrated noise Model	$L_{eq,\ 16h}$<br>35-76 dB<br><br>L_N<br>30-70 dB<br><br>Cut offs*:<br>→<br>$L_{eq,16h}$: 35 dB aircraft; 45dB road	ORs of medication use related to aircraft noise per 10 dB (from Table 4)<br><br>Anxiolytics or hypnotics (noise, OR (95%CI), N)<br>Aircraft: $L_{eq,16h}$: 1.14 (0.97-1.34), N=4,642<br>Aircraft: L_N: 1.10 (0.93- 1.31), N= 4,641<br>Road traffic: $L_{eq,24h}$: 1.11 (0.92-1.34), N=4,642<br><br>Anxiolytics (noise, OR (95%CI), N)<br>Aircraft: $L_{eq,16h}$: 1.28 (1.04-1.57),	study quality:<br>-<br>Cross sectional study design<br><br>conflict of interest:<br>none declared<br><br>funding:<br>stated (HYENA by European Commission and individual grants see

Reference (First author, publication year, S/N) Meta-analysis	Study design	Population: Study region, Sample population, Sample size (M, F, M+F): Age (mean, range), No. of cases / no. of controls or exposed/unexposed, Time of recruitment / follow-up (mean, range), Response (%) (baseline minus loss to follow-up)	Outcome: Disease (ICD-10) Prescription Questionnaire	Exposure: exposure source	Exposure: exposure assessment	Exposure: exposure levels	Results	Comments (study quality [overall assessment according to SIGN/CASP], conflict of interest [stated vs. not stated], funding [financed from public funds vs. financed from industry], confounding, strengths / weaknesses [potential bias, over- or underestimation of potential effects])
		using noise contour maps)<br><br>Sample size:<br>M+F=4861<br>M: 2404, F= 2457<br>For regression models:<br>M+F: 4642<br><br>Age:<br>Mean: 57.9 +/- 7.1 years<br><br>Exposed/unexposed:<br>not applicable<br><br>Time of recruitment / follow-up: 2004-2006			(INM) except UK (national Aircraft Noise Contour model, ANCON)<br>- Maps: 1dB resolution (250x250m spatial resolution)<br><br>Road traffic noise:<br>$L_{eq,\ 24h}$<br>- cut off: 45 dB	noise<br>→ $L_{eq,24h}$: 35 dB<br>→ L_N: 30 dB aircraft; 45 dB road noise<br><br>*All noise values under cut-offs	N=4,642<br>Aircraft: L_N: 1.27 (1.01- 1.59), N= 4,641<br>Road traffic: $L_{eq,24h}$: 1.06 (0.84-1.33), N=4,642<br><br>Hypnotics (noise, OR (95%CI), N)<br>Aircraft: $L_{eq,16h}$: 0.96 (0.76-1.22), N=4,642<br>Aircraft: L_N: 0.90 (0.70- 1.14), N= 4,641<br>Road traffic: $L_{eq,24h}$: 1.28 (0.96-1.71), N=4,642<br><br>Antidepressants (noise, OR (95%CI), N)<br>Aircraft: $L_{eq,16h}$: 1.07 (0.90-1.26), N=4,642	study)→ public funding<br><br>confounding (adjusted for): age, sex, BMI, smoking, alcohol, education, physical activity (and road traffic noise for aircraft noise exposure as well as aircraft noise for road traffic noise exposure in tab. 4)

Reference (First author, publication year, S/N) Meta-analysis	Study design	Population: Study region Sample population Sample size (M, F, M+F): Age (mean, range) No. of cases / no. of controls or exposed/unexposed Time of recruitment / follow-up (mean, range) Response (%) (baseline minus loss to follow-up)	Outcome: Disease (ICD-10) Prescription Questionnaire	Exposure: exposure source	Exposure: exposure assessment	Exposure: exposure levels	Results	Comments (study quality [overall assessment according to SIGN/CASP], conflict of interest [stated vs. not stated], funding [financed from public funds vs. financed from industry], confounding, strengths / weaknesses [potential bias, over- or underestimation of potential effects])
		Response: 39% response for aircraft noise (<50 dB) 45% (50 to < 65 dB) 45% (≥ 65 dB) large differences between countries (for information see Floud et al. 2013)			National noise models - Maps: 1dB resolution (10x10m spatial resolution), except for UK (5 dB resolution)	were set to cut-off values	Aircraft: L_N: 0.96 (0.81- 1.13), N= 4,641 Road traffic: $L_{eq,24h}$: 0.97 (0.78-1.21), N=4,642	strengths/ weaknesses: + adequate consideration of potential confounding + multilevel logistic regression (taking differences between countries into account) + stratified random sampling + adequate exposure measurement + difference between daytime and

Reference (First author, publication year, S/N) Meta-analysis	Study design	Population<br>Study region<br>Sample population<br>Sample size (M, F, M+F):<br>Age (mean, range)<br>No. of cases / no. of controls or exposed/unexposed<br>Time of recruitment / follow-up (mean, range)<br>Response (%)<br>(baseline minus loss to follow-up)	Outcome<br>Disease (ICD-10)<br>Prescription<br>Questionnaire	Exposure<br>exposure source	exposure assessment	exposure levels	Results	Comments<br>(study quality [overall assessment according to SIGN/CASP], conflict of interest [stated vs. not stated], funding [financed from public funds vs. financed from industry], confounding, strengths / weaknesses [potential bias, over- or underestimation of potential effects])
								nighttime aircraft noise<br>- cross-sectional<br>- response not stated in this publication → low response mentioned in Floud et al. 2013<br>- self-reported outcome measurement<br>- non-prescribed medications not mentioned (other associations to hypnotics possible)

Reference (First author, publication year, S/N) Meta-analysis	Study design	Population: Study region Sample population Sample size (M, F, M+F): Age (mean, range) No. of cases / no. of controls or exposed/unexposed Time of recruitment / follow-up (mean, range) Response (%) (baseline minus loss to follow-up)	Outcome: Disease (ICD-10) Prescription Questionnaire	Exposure: exposure source	Exposure: exposure assessment	Exposure: exposure levels	Results	Comments (study quality [overall assessment according to SIGN/CASP], conflict of interest [stated vs. not stated], funding [financed from public funds vs. financed from industry], confounding, strengths / weaknesses [potential bias, over- or underestimation of potential effects])
								- air particulate level not considered
Fuks, 2019, 8750	Longitudinal but cross-sectional analysis	Study region: Germany (North Rhine-Westphalia)<br><br>Sample size: F = 4.874 (baseline sample) F= 834 (follow-up sample) F= 288 (analysis sample with complete data)<br><br>Sample population: Data from ongoing SALIA study	Cognitive function<br><br>CERAD-Plus (Consortium to Establish a Registry on Alzheimer's Disease) neuropsychological assessment battery 4 cognitive domains:	Road 2006	Modelled for most exposed façade (30.5-73.8 dB) L_{DEN}<br><br>L_{NIGHT}	L_{DEN} ≥50dB vs. <50dB<br><br>L_{NIGHT}<br><br>≥50dB vs. <50dB<br>≥40dB vs. <40dB (sensitivity analysis)	physician –diagnosed depression: n = 82, 11.0%<br><br>MMSE<br>*L_{DEN}*<br>Main model: OR = 0.97 (95% CI 0.52-1.81)<br>+ annoyance: OR = 0.98 (95% CI 0.52-1.85)<br>+PM_{10}, NO_2: OR = 0.85 (95% CI 0.44-1.65)<br>+ depression: OR = 0.97 (95% CI 0.52-1.81)	study quality: - (to +)<br>Cross-sectional analysis<br>conflict of interest: stated<br>funding: stated<br>confounding (controlled for): age, smoking, passive smoking, education. Additional models controlled for noise

Reference (First author, publication year, S/N) Meta-analysis	Study design	Population<br>Study region<br>Sample population<br>Sample size (M, F, M+F):<br>Age (mean, range)<br>No. of cases / no. of controls or exposed/unexposed<br>Time of recruitment / follow-up (mean, range)<br>Response (%)<br>(baseline minus loss to follow-up)	Outcome<br>Disease (ICD-10)<br>Prescription<br>Questionnaire	Exposure<br>exposure source	exposure assessment	exposure levels	Results	Comments (study quality [overall assessment according to SIGN/CASP], conflict of interest [stated vs. not stated], funding [financed from public funds vs. financed from industry], confounding, strengths / weaknesses [potential bias, over- or underestimation of potential effects])
		Age:<br>analysis sample: 74.2 years (± 2.2)<br><br>No. of cases<br>-<br><br>Time of recruitment<br>Baseline 1985-1994<br>Follow-up 2007-2010<br><br>Response:<br>Baseline: 70%<br>Follow-up: 17% of baseline sample	semantic memory, episodic memory, constructional praxis, executive functions<br><br>Mini-Mental State Examination (MMSE) -> used for diagnosis of dementia and Alzheimer<br><br>Cognitive scores				L_{NIGHT}<br>Main model: OR = 1.33 (95% CI 0.75-2.36)<br>+ annoyance: OR = 1.38 (95% CI 0.76-2.49)<br>+PM_{10}, NO_2: OR = 1.26 (95% CI 0.68-2.34)<br>+ depression: OR = 1.33 (95% CI 0.75-2.36)<br><br>Total score<br>L_{DEN}<br>Main model: OR = 1.69 (95% CI 0.94-3.04)<br>+ annoyance: OR = 1.84 (95% CI 1.01-3.38)	annoyance, PM_{10} & NO_2, or diagnosed depression<br><br>strengths, weaknesses:<br>-Cross-sectional analysis within longitudinal study, with basic information about chronology between exposition and outcome<br>-very low follow-up and only a small

Reference (First author, publication year, S/N) Meta-analysis	Study design	Population: Study region Sample population Sample size (M, F, M+F): Age (mean, range) No. of cases / no. of controls or exposed/unexposed Time of recruitment / follow-up (mean, range) Response (%) (baseline minus loss to follow-up)	Outcome: Disease (ICD-10) Prescription Questionnaire	Exposure: exposure source	Exposure: exposure assessment	Exposure: exposure levels	Results	Comments (study quality [overall assessment according to SIGN/CASP], conflict of interest [stated vs. not stated], funding [financed from public funds vs. financed from industry], confounding, strengths / weaknesses [potential bias, over- or underestimation of potential effects])
			dichotomized using age and education standardized z-scores > 0 to indicate impaired cognition.<br><br>Note: Only results for MMSE and total score extracted and used				+PM_{10}, NO_2: OR = 1.87 (95% CI 0.99-3.52)<br>+ depression: OR = 1.68 (95% CI 0.93-3.04)<br><br>*LNIGHT*<br>Main model: OR = 0.87 (95% CI 0.51-1.49)<br>+ annoyance: OR = 0.95 (95% CI 0.54-1.65)<br>+PM_{10}, NO_2: OR = 0.83 (95% CI 0.47-1.48)<br>+ depression: OR = 0.87(95% CI 0.51-1.49)	subset with noise data included in the analysis<br>- noise dichotomized<br>- no threshold for cognitive disorder used<br><br>+ adequate definition and assessment of outcome<br>+adequate control for confounders, including air particulates<br>+ adequate exposure

Reference (First author, publication year, S/N) Meta-analysis	Study design	Population: Study region, Sample population, Sample size (M, F, M+F): Age (mean, range), No. of cases / no. of controls or exposed/unexposed, Time of recruitment / follow-up (mean, range), Response (%) (baseline minus loss to follow-up)	Outcome: Disease (ICD-10), Prescription, Questionnaire	Exposure: exposure source	Exposure: exposure assessment	Exposure: exposure levels	Results	Comments (study quality [overall assessment according to SIGN/CASP], conflict of interest [stated vs. not stated], funding [financed from public funds vs. financed from industry], confounding, strengths / weaknesses [potential bias, over- or underestimation of potential effects]) assessment
Genera al, 2019a, 8488	Case-control study with Cross-sectional analysis within cohort study (NESDA	Study region: Netherlands (mostly recruited from cities of Amsterdam, Leiden and Groningen)<br><br>Sample size: M+F=2,980 M= 1,007, F=1,973<br><br>Sample population: "persons with a range of pathophysiology were included" -> Recruitment from primary	Cases: current diagnosis of depressive disorders (major depressive disorder and dysthymia) and anxiety disorder (panic disorder, agrophobia, generalized anxiety disorder, social phobia) -> CIDI interview	Traffic noise = Road+ Rail+ Air<br><br>Noise data from 2007<br><br>->combined analysis	L_{DEN}	-	Depressive disorder/ and or anxiety disorder<br>Cases: n=1,783/ Controls (n=1,197)<br>OR: 1.17, 95% CI 1.05-1.30<br><br>Depressive disorder:<br>Cases: n=1,275/ Controls (n=1,197)<br>OR: 1.17, 95% CI 1.03-1.32<br><br>Anxiety disorder:<br>Cases: n=1,363/ Controls (n=1,197)<br>OR: 1.22, 95% CI 1.09-1.38	study quality: -to(+) Cross-sectional analysis<br>conflict of interest: stated<br>funding: stated<br>confounding (controlled for): sex, age, education and household income.<br>strengths, weaknesses:

Reference (First author, publication year, S/N) Meta-analysis	Study design	Population: Study region Sample population Sample size (M, F, M+F): Age (mean, range) No. of cases / no. of controls or exposed/unexposed Time of recruitment / follow-up (mean, range) Response (%) (baseline minus loss to follow-up)	Outcome: Disease (ICD-10) Prescription Questionnaire	Exposure: exposure source	Exposure: exposure assessment	Exposure: exposure levels	Results	Comments (study quality [overall assessment according to SIGN/CASP], conflict of interest [stated vs. not stated], funding [financed from public funds vs. financed from industry], confounding, strengths / weaknesses [potential bias, over- or underestimation of potential effects])
	study)	care (54%), specialized mental health care (27%), community (19%)<br><br>Age:<br>43 years (14) controls<br>41 years (12) cases<br><br>No. of cases / no. of controls<br>See results<br><br>Time of recruitment<br>2004-2007	(DSM-IV criteria)<br>Controls: individuals without currant depression and/ or anxiety disorder diagnosis<br><br>Measures:<br>-Inventory of Depression Symptomatology (IDS)<br>-Beck Anxiety Inventory (BAI)				(linear regression for association of disease severity and noise was also performed: results are not displayed)	-Cross-sectional analysis within longitudinal study, with basic information about chronology between exposition and outcome<br><br>+ adequate definition and assessment of outcome<br>+adequate control for confounders<br>+ adequate exposure assessment

Reference (First author, publication year, S/N) Meta-analysis	Study design	Population: Study region Sample population Sample size (M, F, M+F): Age (mean, range) No. of cases / no. of controls or exposed/unexposed Time of recruitment / follow-up (mean, range) Response (%) (baseline minus loss to follow-up)	Outcome: Disease (ICD-10) Prescription Questionnaire	Exposure: exposure source	Exposure: exposure assessment	Exposure: exposure levels	Results	Comments (study quality [overall assessment according to SIGN/CASP], conflict of interest [stated vs. not stated], funding [financed from public funds vs. financed from industry], confounding, strengths / weaknesses [potential bias, over- or underestimation of potential effects])
		Response: Unclear	- Fear questionnaire (FQ)					+ high statistical power<br>+ ethics approval by Ethical Committee of participating universities
Generaal, 2019b, 8757	Cross-sectional analysis of pooled data from 8	Study region: Netherlands<br><br>Sample size: N = 32,487<br>*NEMESIS-2*: n = 6,381<br>*HELIUS*: n = 4,634<br>*NTR*: n = 11,388	Depression<br><br>*NEMESIS-2 & NESDA*: 12-month prevalence based on semi-structured	Traffic noise (road, rail and air combined, possibly as arithmetic average)	Noise modelled by the Netherlands Environmental Assessment Agency, available at the six-digit postal	-	Prevalence of depression (%)<br>*NEMESIS-2*: 6.4<br>*HELIUS*: 7.3<br>*NTR*: 6.3<br>*NESDA*: 52<br>*HOORN*: 5.1<br>*LASA*: 5.0<br>*NL-SH*: 5.8	study quality:<br>-<br>Cross-sectional analysis<br>conflict of interest: stated<br>funding: stated

		Population	Outcome	Exposure				
**Reference (First author, publication year, S/N) Meta-analysis**	**Study design**	**Study region<br>Sample population<br>Sample size (M, F, M+F):<br>Age (mean, range)<br>No. of cases / no. of controls or exposed/unexposed<br>Time of recruitment / follow-up (mean, range)<br>Response (%)<br>(baseline minus loss to follow-up)**	**Disease (ICD-10)<br>Prescription<br>Questionnaire**	**exposure source**	**exposure assessment**	**exposure levels**	**Results**	**Comments<br>(study quality [overall assessment according to SIGN/CASP], conflict of interest [stated vs. not stated], funding [financed from public funds vs. financed from industry], confounding, strengths / weaknesses [potential bias, over- or underestimation of potential effects])**
	population-based or case-control cohort studies (NEMESIS-2, HELIUS, NTR, HOO	*NESDA*: n = 2,472<br>*HOORN*: n = 2,667<br>*LASA*: n = 1,893<br>*NL-SH*: n = 1,575<br>*Generations^{2}*: n = 1,477<br><br>Sample population:<br>Netherlands Mental Health Survey & Incidence Study-2<br>*NEMESIS-2*: (cohort) general population aged 18-64 years nationwide<br><br>Healthy Life in an Urban Setting Study<br>*HELIUS*: (cohort) different	Composite International Diagnostic Interview (CIDI); people with anxiety disorders excluded from control group<br><br>*HELIUS*: 9-item Patient Health Questionnaire (PHQ-9 ≥10)<br><br>*NTR*: Hospital Anxiety and	Noise data from 2007-2008	code level and matched to addresses.<br><br>L_{DEN}		*Generations^{2}*: 4.0<br><br>Traffic noise (no info on increase per dB):<br>Pooled analysis<br>OR = 1.05 (95% CI 0.96-1.15)<br><br>*NEMESIS-2*: OR = 1.02 (95% CI 0.91-1.15)<br>*HELIUS*: OR = 0.95 (95% CI 0.84-1.09)<br>*NTR*: OR = 1.01 (95% CI 0.93-1.10)<br>*NESDA*: OR = 1.17 (95% CI 1.03-1.32)<br>*HOORN*: OR = 1.19 (95% CI 1.01-1.39)<br>*LASA*: OR = 1.21 (95% CI 0.97-1.50)<br>*NL-SH*: OR = 0.88 (95% CI 0.71-1.07)<br>*Generations^{2}*: OR = 1.04 (95% CI	confounding (controlled for): sex, age, years of education and income.<br>strengths, weaknesses:<br>-Cross-sectional analysis within longitudinal studies,<br>- Violation of chronology between exposition and outcome for 4 of 8 studies -> exposition assessed after

Reference (First author, publication year, S/N) Meta-analysis	Study design	Population<br>Study region<br>Sample population<br>Sample size (M, F, M+F):<br>Age (mean, range)<br>No. of cases / no. of controls or exposed/unexposed<br>Time of recruitment / follow-up (mean, range)<br>Response (%)<br>(baseline minus loss to follow-up)	Outcome<br>Disease (ICD-10)<br>Prescription<br>Questionnaire	Exposure<br>exposure source	exposure assessment	exposure levels	Results	Comments (study quality [overall assessment according to SIGN/CASP], conflict of interest [stated vs. not stated], funding [financed from public funds vs. financed from industry], confounding, strengths / weaknesses [potential bias, over- or underestimation of potential effects])
	RN, LASA; NL-SH, Generations [2], NESDA)	ethnic groups in Amsterdam aged 18-70 years<br><br>Netherlands Twin Register *NTR*: (cohort): focus on gene and environment on development. Includes 11,388 individuals selected (siblings, twins, multiples, parents, spouses)<br><br>Netherlands Study of Depression and Anxiety *NESDA*: from urban and rural areas from Amsterdam, Leiden, Groningen (18-65	Depression Scale (HADS-D ≥8)<br><br>*NESDA*: CIDI<br><br>*HOORN*: Center for Epidemiologic Studies Depression Scale (CES-D ≥ 23)<br><br>*LASA*: CES-D ≥23<br><br>*NL-SH*: Four-Dimensiona				0.77-1.40)	outcome<br>- assessment of noise (mean but no energetic summation) missing<br>-heterogeneous study population<br>- Low response rate in some cohorts<br>- air particulate level estimated but not considered as a confounder<br><br>+ large sample size<br>+ adequate (but

Reference (First author, publication year, S/N) Meta-analysis	Study design	Population<br>Study region<br>Sample population<br>Sample size (M, F, M+F):<br>Age (mean, range)<br>No. of cases / no. of controls or exposed/unexposed<br>Time of recruitment / follow-up (mean, range)<br>Response (%)<br>(baseline minus loss to follow-up)	Outcome<br>Disease (ICD-10)<br>Prescription<br>Questionnaire	Exposure<br>exposure source	exposure assessment	exposure levels	Results	Comments (study quality [overall assessment according to SIGN/CASP], conflict of interest [stated vs. not stated], funding [financed from public funds vs. financed from industry], confounding, strengths / weaknesses [potential bias, over- or underestimation of potential effects])
		years)<br><br>New Hoorn Study<br>*HOORN*: cohort study with focus on diabetes (n=2,807, aged 40-65 years at baseline)<br><br>Longitudinal Ageing Study Amsterdam<br>*LASA*: Focusses on older individuals around Amsterdam, Oss and Zwolle (n = 3,107 aged 55-85 years at baseline in 1992/1993)<br><br>Netherlands Longitudinal	l Symptom Questionnaire (4DSQ ≥ 6)<br><br>*Generations*[2]: Beck Depression Inventory-II (BDI-II ≥ 20)					heterogeneous) definition and assessment of outcome<br>+adequate control for confounders

Reference (First author, publication year, S/N) Meta-analysis	Study design	Population: Study region Sample population Sample size (M, F, M+F): Age (mean, range) No. of cases / no. of controls or exposed/unexposed Time of recruitment / follow-up (mean, range) Response (%) (baseline minus loss to follow-up)	Outcome: Disease (ICD-10) Prescription Questionnaire	Exposure: exposure source	Exposure: exposure assessment	Exposure: exposure levels	Results	Comments (study quality [overall assessment according to SIGN/CASP], conflict of interest [stated vs. not stated], funding [financed from public funds vs. financed from industry], confounding, strengths / weaknesses [potential bias, over- or underestimation of potential effects])
		Study on Hearing<br>*NL-SH*: focus on hearing impairment (n=1,575, aged 18-64 years)<br>*Generations*[2]: Follows first-time pregnant women (n=1,477)<br><br>Age, mean years (SD):<br>*NEMESIS-2*: 44 (13)<br>*HELIUS*: 46 (14)<br>*NTR*: 47 (13)<br>*NESDA*: 42 (13)<br>*HOORN*: 53 (7)<br>*LASA*: 71 (9)<br>*NL-SH*: 46 (12)						

Reference (First author, publication year, S/N) Meta-analysis	Study design	Population: Study region Sample population Sample size (M, F, M+F): Age (mean, range) No. of cases / no. of controls or exposed/unexposed Time of recruitment / follow-up (mean, range) Response (%) (baseline minus loss to follow-up)	Outcome: Disease (ICD-10) Prescription Questionnaire	Exposure: exposure source	exposure assessment	exposure levels	Results	Comments (study quality [overall assessment according to SIGN/CASP], conflict of interest [stated vs. not stated], funding [financed from public funds vs. financed from industry], confounding, strengths / weaknesses [potential bias, over- or underestimation of potential effects])
		Generations[2]: 35 (47)<br><br>Sex (women in %) :<br>*NEMESIS-2*: 55<br>*HELIUS*: 54<br>*NTR*: 62<br>*NESDA*: 66<br>*HOORN*: 53<br>*LASA*: 55<br>*NL-SH*: 64<br>*Generations*[2]: 100<br><br>No. of cases (%)<br>See results<br><br>Time of recruitment						

Reference (First author, publication year, S/N) Meta-analysis	Study design	Population<br>Study region<br>Sample population<br>Sample size (M, F, M+F):<br>Age (mean, range)<br>No. of cases / no. of controls or exposed/unexposed<br>Time of recruitment / follow-up (mean, range)<br>Response (%)<br>(baseline minus loss to follow-up)	Outcome<br>Disease (ICD-10)<br>Prescription<br>Questionnaire	Exposure<br>exposure source	exposure assessment	exposure levels	Results	Comments (study quality [overall assessment according to SIGN/CASP], conflict of interest [stated vs. not stated], funding [financed from public funds vs. financed from industry], confounding, strengths / weaknesses [potential bias, over- or underestimation of potential effects])
		NEMESIS-2: 2007-2009<br>*HELIUS*: 2011-2015<br>*NTR*: 2009-2010<br>*NESDA*: 2004-2007<br>*HOORN*: 2006-2007<br>*LASA*: 2005-2006<br>*NL-SH*: 2006-2008<br>*Generations*[2]: 2009-2015<br><br>Response<br>*NEMESIS-2*: 58% (de Graaf et al. 2010)<br>*HELIUS*: 28% (Snijder et al. 2017)<br>*NTR*: 52% (Willemsen et al. 2013)						

Reference (First author, publication year, S/N) Meta-analysis	Study design	Population<br>Study region<br>Sample population<br>Sample size (M, F, M+F):<br>Age (mean, range)<br>No. of cases / no. of controls or exposed/unexposed<br>Time of recruitment / follow-up (mean, range)<br>Response (%)<br>(baseline minus loss to follow-up)	Outcome<br>Disease (ICD-10)<br>Prescription<br>Questionnaire	Exposure<br>exposure source	exposure assessment	exposure levels	Results	Comments (study quality [overall assessment according to SIGN/CASP], conflict of interest [stated vs. not stated], funding [financed from public funds vs. financed from industry], confounding, strengths / weaknesses [potential bias, over- or underestimation of potential effects])
		NESDA: 30% (Pennix et al. 2011, van Graaf et al. 2010)<br>*HOORN*: 43% (Bouwman et al. 2011)<br>*LASA*: 60% initial response (Hoogendijk et al. 2016)<br>*NL-SH*: 35% (Goderie et al. 2019)<br>*Generations*[2]: not calculated due to recruitment methods (see Wernand et al. 2014)						
Greiser, E, 2010, H-2889<br>No (used	Case-control study	Study region:<br>Region around Cologne-Bonn Airport, Germany (Cologne, Rhein-Sieg area, Rheinisch-Bergischer area)	Secondary data from eight insurance companies (ICD-9/10)	Aircraft noise	L_N (22-06h)<br><br>L_D (06-22h)<br><br>L_{eq} (23-01h)	L_{eq}<br>≥ 35 dB<br><br>Other exposure	Anxiety and phobia:<br>- no elevated disease risk<br><br>Depression<br>- elevated risk for women to get a	study quality:<br>-<br><br>conflict of interest:<br>not stated

Reference (First author, publication year, S/N) Meta-analysis	Study design	Population: Study region Sample population Sample size (M, F, M+F): Age (mean, range) No. of cases / no. of controls or exposed/unexposed Time of recruitment / follow-up (mean, range) Response (%) (baseline minus loss to follow-up)	Outcome: Disease (ICD-10) Prescription Questionnaire	Exposure: exposure source	Exposure: exposure assessment	Exposure: exposure levels	Results	Comments (study quality [overall assessment according to SIGN/CASP], conflict of interest [stated vs. not stated], funding [financed from public funds vs. financed from industry], confounding, strengths / weaknesses [potential bias, over- or underestimation of potential effects])
two interaction terms in model, difficult to interpret)		Sample population: Residents of the study region with data from 8 insurance companies (55% of the whole study population)<br><br>Sample size: M+F=511,742 M=223,559, F=288,183<br><br>Age: > 39 yrs.<br><br>No. of cases/ no. of controls: cases:	Anxiety/ Phobia: ICD-9: 300 (anxiety, dissociative & somatoform disorders) / ICD-10: F40 (Phobic anxiety disorders), F41 (Other anxiety disorders)<br><br>Depression: ICD-9: 311 (Depressive		L_{eq} (03-05h)<br><br>L_{eq}<br><br>2004 (basis are 6 noisiest months of the year) Noise modeling	assesments ≥ 40 dB	stationary treatment for depression - no risk for men<br><br>Psychosis: - inconsistent results - elevated risk for women only for the timeframe "night: 22-6h" and between "23-1h"<br><br>Figures 14-16 show graphics, but interaction terms (Age*Noise and Socialsupport*Noise) included in the model and not reported	funding: financed from public funds (German Federal Institute for Environmental Research)<br><br>confounding (adjusted for): Age Considered: road and rail noise, social welfare in region, density of retirement homes

Reference (First author, publication year, S/N) Meta-analysis	Study design	Population<br>Study region<br>Sample population<br>Sample size (M, F, M+F):<br>Age (mean, range)<br>No. of cases / no. of controls or exposed/unexposed<br>Time of recruitment / follow-up (mean, range)<br>Response (%)<br>(baseline minus loss to follow-up)	Outcome<br>Disease (ICD-10)<br>Prescription<br>Questionnaire	Exposure<br>exposure source	exposure assessment	exposure levels	Results	Comments<br>(study quality [overall assessment according to SIGN/CASP], conflict of interest [stated vs. not stated], funding [financed from public funds vs. financed from industry], confounding, strengths / weaknesses [potential bias, over- or underestimation of potential effects])
		Number of cases:<br>Anxiety and phobia:<br>M+F: 2,344<br>M: 709<br>F: 1,635<br>Depression:<br>M+F: 3,136<br>M: 981<br>F: 2,155<br>Psychoses:<br>M+F: 105,22<br>M: 3,599<br>F: 6,923<br><br>Time of recruitment/ follow-up: Variation of	disorder, not elsewhere classified)/<br>ICD-10: F33 (Major depressive disorder recurrent), F34 (Persistent mood disorders)<br><br>Psychoses:<br>ICD-9: 290 (Dementias), 291 (Alcohol induced mental disorder), 292 (drug					strengths/ weaknesses:<br>+ objective and reliable outcome measurement: analysis of ICD9/10-coded insurance data<br>+ exposure measurement<br>+ consideration of other sources of noise (railway/road traffic noise)<br>- no consideration of

Reference (First author, publication year, S/N) Meta-analysis	Study design	**Population**<br>Study region<br>Sample population<br>Sample size (M, F, M+F):<br>Age (mean, range)<br>No. of cases / no. of controls or exposed/unexposed<br>Time of recruitment / follow-up (mean, range)<br>Response (%)<br>(baseline minus loss to follow-up)	**Outcome**<br>Disease (ICD-10)<br>Prescription<br>Questionnaire	**Exposure**<br>exposure source	exposure assessment	exposure levels	Results	Comments (study quality [overall assessment according to SIGN/CASP], conflict of interest [stated vs. not stated], funding [financed from public funds vs. financed from industry], confounding, strengths / weaknesses [potential bias, over- or underestimation of potential effects])
		observation between 2 and 6 years<br><br>Response:<br>55.4%	induced mental disorder), 294 (Persistent mental disorders due to conditions classified elsewhere), 295 (Schizophrenic disorders), 296 (Episodic mood disorders), 297 (Delusional disorders), 298 (Other non-organic psychoses), 299					outcome-specific confounders (e.g. lifestyle confounder)<br>- adjustment for interaction term age*aircraft noise makes interpretation of results complicated (no significant coefficient)<br>- Some ecological measurements for confounders used (prevalence of local social welfare; density of nursing home beds)

Reference (First author, publication year, S/N) Meta-analysis	Study design	Population: Study region, Sample population, Sample size (M, F, M+F): Age (mean, range), No. of cases / no. of controls or exposed/unexposed, Time of recruitment / follow-up (mean, range), Response (%) (baseline minus loss to follow-up)	Outcome: Disease (ICD-10) Prescription Questionnaire	Exposure: exposure source	Exposure: exposure assessment	Exposure: exposure levels	Results	Comments (study quality [overall assessment according to SIGN/CASP], conflict of interest [stated vs. not stated], funding [financed from public funds vs. financed from industry], confounding, strengths / weaknesses [potential bias, over- or underestimation of potential effects])
			(Pervasive developmental disorders)/ ICD-10: F02 (Dementia in other diseases classified elsewhere), F03 (Unspecific dementia), F04 (Amnestic disorder due to known physiological conditions), F05 (Delirium due to					- chronology of exposure and outcome not clear<br>- only respondents over 39 yrs. analyzed<br>- air particulate level not considered

Reference (First author, publication year, S/N) Meta-analysis	Study design	Population: Study region Sample population Sample size (M, F, M+F): Age (mean, range) No. of cases / no. of controls or exposed/unexposed Time of recruitment / follow-up (mean, range) Response (%) (baseline minus loss to follow-up)	Outcome: Disease (ICD-10) Prescription Questionnaire	Exposure: exposure source	Exposure: exposure assessment	Exposure: exposure levels	Results	Comments (study quality [overall assessment according to SIGN/CASP], conflict of interest [stated vs. not stated], funding [financed from public funds vs. financed from industry], confounding, strengths / weaknesses [potential bias, over- or underestimation of potential effects])
			known physiol. cond.), F06 (Other mental disorders due to known physiol. cond.), F09 (Unspecific mental disorders due to known physiol. cond.)					
Halonen, Jaana I. 2014, 959, Finland Yes	Cross-sectional study	Study region: Turku, Helsinki and Vantaa, Finland<br><br>Study population: Public sector employees	Psychotropic medication use:<br><br>Anxiolytics (N05B)<br>Hypnotics (N05C)	Residential road traffic	L_{DEN} (Modelled)	Five categorization levels:<br><br>≤45dB (Ref.),	Associations of road traffic noise and psychotropic medication use among men (from Table 3)<br>Noise level (dB) OR (95%) n	Study quality: -<br>Cross-sectional study design<br><br>Conflict of interests: None stated

		Population	Outcome	Exposure				
**Reference (First author, publication year, S/N) Meta-analysis**	**Study design**	**Study region<br>Sample population<br>Sample size (M, F, M+F):<br>Age (mean, range)<br>No. of cases / no. of controls or exposed/unexposed<br>Time of recruitment / follow-up (mean, range)<br>Response (%)<br>(baseline minus loss to follow-up)**	**Disease (ICD-10)<br>Prescription<br>Questionnaire**	**exposure source**	**exposure assessment**	**exposure levels**	**Results**	**Comments<br>(study quality [overall assessment according to SIGN/CASP], conflict of interest [stated vs. not stated], funding [financed from public funds vs. financed from industry], confounding, strengths / weaknesses [potential bias, over- or underestimation of potential effects])**
		Sample size:<br>M+F=15,611<br>M= 3086, F=12,525<br><br>Age:<br>Mean: 50.3 years<br>Range: 21-76 years<br><br>Exposed/unexposed:<br>See results<br><br>Time of recruitment/follow-up:<br>2000-2010<br><br>Response:	Antidepressants (N06A) during the year of survey obtained from National Prescription Register			45.1-50 dB,<br>50.1-55 dB,<br>55.1-60 dB,<br>>60 dB<br><br>mean level of RTN at participant home addresses: 52 dB (SD: 8.1,	≤45 1.00 402<br>45.1-50 0.90 (0.60–1.35) 615<br>50.1-55 1.25 (0.84–1.87) 520<br>55.1-60 dB 0.87 (0.54–1.41) 327<br>>60 dB 0.79 (0.50–1.25) 391<br><br>Associations of road traffic noise and	Funding:<br>EU ERA-AGE2 program funded by the Academy of Finland and the Ministry of Social Affairs and Health. Kivimäki supported by the Medical Research Council and the Finnish Work Environment Fund and a professorial fellowship from the UK Economic and

Reference (First author, publication year, S/N) Meta-analysis	Study design	Population: Study region, Sample population, Sample size (M, F, M+F): Age (mean, range), No. of cases / no. of controls or exposed/unexposed, Time of recruitment / follow-up (mean, range), Response (%) (baseline minus loss to follow-up)	Outcome: Disease (ICD-10) Prescription Questionnaire	Exposure: exposure source	Exposure: exposure assessment	Exposure: exposure levels	Results	Comments (study quality [overall assessment according to SIGN/CASP], conflict of interest [stated vs. not stated], funding [financed from public funds vs. financed from industry], confounding, strengths / weaknesses [potential bias, over- or underestimation of potential effects])
		Unclear<br>69% for those who had left the participating organization, but unclear for others				range 18-79 dB)	psychotropic medication use among women (from Table 4)<br>Adjusted model<br>Noise level (dB) / OR (95%) / N<br>≤45 / 1.00 / 1610<br>45.1-50 / 0.93 (0.78–1.11) / 2398<br>50.1-55 / 0.86 (0.72–1.03) / 2059<br>55.1-60 / 1.00 (0.82– / 1404	Social Research Council.<br><br>Confounding (controlled for): Age, occupational status, level of education, size of residence, marital status, job strain, chronic disease, area-level socioeconomic status, population density<br><br>Strength/weaknesses:

Reference (First author, publication year, S/N) Meta-analysis	Study design	Population: Study region Sample population Sample size (M, F, M+F): Age (mean, range) No. of cases / no. of controls or exposed/unexposed Time of recruitment / follow-up (mean, range) Response (%) (baseline minus loss to follow-up)	Outcome: Disease (ICD-10) Prescription Questionnaire	Exposure: exposure source	Exposure: exposure assessment	Exposure: exposure levels	Results	Comments (study quality [overall assessment according to SIGN/CASP], conflict of interest [stated vs. not stated], funding [financed from public funds vs. financed from industry], confounding, strengths / weaknesses [potential bias, over- or underestimation of potential effects])
							1.21)<br>>60 0.96 (0.80–1.17) 1478<br><br>Data not shown:<br>Stratification by trait anxiety score found no associations for traffic noise and psychotropic medication use among men or for traffic noise and the two outcomes among women.	+ Appropriate measurement of traffic noise levels, although the modelling was done before the survey for some and after for others<br>+ objective source for covariate information (age, sex, SES, addresses) information obtained through employers registers or Population Register

Reference (First author, publication year, S/N) Meta-analysis	Study design	Population: Study region Sample population Sample size (M, F, M+F): Age (mean, range) No. of cases / no. of controls or exposed/unexposed Time of recruitment / follow-up (mean, range) Response (%) (baseline minus loss to follow-up)	Outcome: Disease (ICD-10) Prescription Questionnaire	Exposure: exposure source	Exposure: exposure assessment	Exposure: exposure levels	Results	Comments (study quality [overall assessment according to SIGN/CASP], conflict of interest [stated vs. not stated], funding [financed from public funds vs. financed from industry], confounding, strengths / weaknesses [potential bias, over- or underestimation of potential effects])
								Center +good objective outcome measurement (National Prescription Register) - cross-sectional design - limited data on area-level confounders (air quality, green space, unsafe neighborhood) - other noise exposure not considered - May not be

Reference (First author, publication year, S/N) Meta-analysis	Study design	Population: Study region Sample population Sample size (M, F, M+F): Age (mean, range) No. of cases / no. of controls or exposed/unexposed Time of recruitment / follow-up (mean, range) Response (%) (baseline minus loss to follow-up)	Outcome: Disease (ICD-10) Prescription Questionnaire	Exposure: exposure source	Exposure: exposure assessment	Exposure: exposure levels	Results	Comments (study quality [overall assessment according to SIGN/CASP], conflict of interest [stated vs. not stated], funding [financed from public funds vs. financed from industry], confounding, strengths / weaknesses [potential bias, over- or underestimation of potential effects])
								generalized to other countries due to window construction of Finnish houses (less noise due to double windows)<br>- unclear response rate (selection bias)<br>- anxiolytics may be indicative of sleep disorders (not differential)<br>- high percentage of women (80%) (generalizability)<br>- healthy worker bias

Reference (First author, publication year, S/N) Meta-analysis	Study design	Population: Study region Sample population Sample size (M, F, M+F): Age (mean, range) No. of cases / no. of controls or exposed/unexposed Time of recruitment / follow-up (mean, range) Response (%) (baseline minus loss to follow-up)	Outcome: Disease (ICD-10) Prescription Questionnaire	Exposure: exposure source	Exposure: exposure assessment	Exposure: exposure levels	Results	Comments (study quality [overall assessment according to SIGN/CASP], conflict of interest [stated vs. not stated], funding [financed from public funds vs. financed from industry], confounding, strengths / weaknesses [potential bias, over- or underestimation of potential effects])
Halonen, J.I., 2013, H-2890, Finland<br><br>(Evaluated together with Halonen et al. 2014)	Cross-sectional study	see Halonen et al. 2014	see Halonen et al. 2014	see Halonen et al. 2014	see Halonen et al. 2014	see Halonen et al. 2014	Odds ratios and 95% confidence intervals for medication use by road traffic noise (L_{DEN}), from Table 2<br><br>Noise level (dB) / Antidepressant OR (95% CI)<br>≤45 dB: 1.00<br>45.1-50: 0.99 (0.85-1.24)<br>50.1-55: 1.03 (0.80-1.20)<br>55.1-60: 0.98 (0.83-1.29)	see Halonen et al. 2014

Reference (First author, publication year, S/N) Meta-analysis	Study design	Population: Study region Sample population Sample size (M, F, M+F): Age (mean, range) No. of cases / no. of controls or exposed/unexposed Time of recruitment / follow-up (mean, range) Response (%) (baseline minus loss to follow-up)	Outcome: Disease (ICD-10) Prescription Questionnaire	Exposure: exposure source	Exposure: exposure assessment	Exposure: exposure levels	Results		Comments (study quality [overall assessment according to SIGN/CASP], conflict of interest [stated vs. not stated], funding [financed from public funds vs. financed from industry], confounding, strengths / weaknesses [potential bias, over- or underestimation of potential effects])
							>60	0.97 (0.78-1.20)	
							Noise level (dB)	Anxiolytics OR (95% CI)	
							≤45	1.00	
							45.1-50 dB	0.88 (0.58-1.10)	
							50.1-55 dB	0.99 (0.73-1.36)	
							55.1-60 dB	0.98 (0.69-1.38)	
							>60 dB	0.92	

Reference (First author, publication year, S/N) Meta-analysis	Study design	Population: Study region Sample population Sample size (M, F, M+F): Age (mean, range) No. of cases / no. of controls or exposed/unexposed Time of recruitment / follow-up (mean, range) Response (%) (baseline minus loss to follow-up)	Outcome: Disease (ICD-10) Prescription Questionnaire	Exposure: exposure source	Exposure: exposure assessment	Exposure: exposure levels	Results		Comments (study quality [overall assessment according to SIGN/CASP], conflict of interest [stated vs. not stated], funding [financed from public funds vs. financed from industry], confounding, strengths / weaknesses [potential bias, over- or underestimation of potential effects])
								(0.65-1.28)	
							Noise level (dB)	Hypnotic use OR (95% CI)	
							≤45	1.00	
							45.1-50	0.84 (0.66-1.07)	
							50.1-55	0.87 (0.69-1.11)	
							55.1-60	0.86 (0.66-1.12)	
							>60 dB	0.83 (0.64-1.08)	

Reference (First author, publication year, S/N) Meta-analysis	Study design	Population: Study region Sample population Sample size (M, F, M+F): Age (mean, range) No. of cases / no. of controls or exposed/unexposed Time of recruitment / follow-up (mean, range) Response (%) (baseline minus loss to follow-up)	Outcome: Disease (ICD-10) Prescription Questionnaire	Exposure: exposure source	Exposure: exposure assessment	Exposure: exposure levels	Results		Comments (study quality [overall assessment according to SIGN/CASP], conflict of interest [stated vs. not stated], funding [financed from public funds vs. financed from industry], confounding, strengths / weaknesses [potential bias, over- or underestimation of potential effects])
							Noise level (dB)	Any medication use OR (95% CI)	
							≤45	1.00	
							45.1-50	0.93 (0.79-1.09)	
							50.1-55	0.92 (0.78-1.08)	
							55.1-60	0.99 (0.82-1.18)	
							>60	0.93 (0.78-1.11)	

Reference (First author, publication year, S/N) Meta-analysis	Study design	Population: Study region, Sample population, Sample size (M, F, M+F): Age (mean, range), No. of cases / no. of controls or exposed/unexposed, Time of recruitment / follow-up (mean, range), Response (%) (baseline minus loss to follow-up)	Outcome: Disease (ICD-10), Prescription, Questionnaire	Exposure: exposure source	Exposure: exposure assessment	Exposure: exposure levels	Results	Comments (study quality [overall assessment according to SIGN/CASP], conflict of interest [stated vs. not stated], funding [financed from public funds vs. financed from industry], confounding, strengths / weaknesses [potential bias, over- or underestimation of potential effects])
							Adjusted for age, sex, occupational status, level of education, size of residence, marital status, job strain, area-level socioeconomic status, and population density	
He, 2019, 8831	cohort	Study region: Canada (Montreal)<br><br>Sample size: F = 140,456<br><br>Sample population: Hospital records of women with one pregnancy during the study period; exclusion of women with	Depression<br><br>ICD-9 296.2, 296.3, 300.4, 309.28, 311; ICD-10 F32-F34.1, F41.2	Total outdoor noise (land-use regression model): mostly road traffic noise, vicinity to	$L_{Aeq, 24h}$<br><br>L_{DEN}<br><br>L_{NIGHT}<br><br>Noise matched to 6-digit postal code	< 55 dB<br>55.0-59.9 dB<br>60.0-64.9 dB<br>≥ 65 dB	Hazard ratios (adjusted)<br><br>$L_{Aeq, 24h}$<br><br>< 55 dB: reference<br>55.0-59.9 dB: HR=0.99 (0.83-1.19)<br>60.0-64.9 dB: HR=1.06 (95%CI 0.87-1.29)<br>≥ 65 dB: HR=0.92 (95% CI 0.65-1.32)<br><br>L_{DEN}	study quality: -<br>conflict of interest: stated<br>funding: stated<br>confounding (controlled for): maternal age, parity, multiple pregnancy, stillbirth,

Reference (First author, publication year, S/N) Meta-analysis	Study design	Population: Study region Sample population Sample size (M, F, M+F): Age (mean, range) No. of cases / no. of controls or exposed/unexposed Time of recruitment / follow-up (mean, range) Response (%) (baseline minus loss to follow-up)	Outcome: Disease (ICD-10) Prescription Questionnaire	Exposure: exposure source	Exposure: exposure assessment	Exposure: exposure levels	Results	Comments (study quality [overall assessment according to SIGN/CASP], conflict of interest [stated vs. not stated], funding [financed from public funds vs. financed from industry], confounding, strengths / weaknesses [potential bias, over- or underestimation of potential effects])
		mental disorders before or during pregnancy)<br><br>Age at baseline:<br>*With Depression*<br>< 25 years: n=183<br>25-29 years n=285<br>30-34 years: n=271<br>≥ 35 years: n=220<br><br>*No mental disorder*<br>< 25 years: n=18,709<br>25-29 years n=35,644<br>30-34 years: n=46,175<br>≥ 35 years: n=37,034		aircraft and railway traffic additional predictors of noise)<br><br>Model bases on outdoor samples collected in in 2010 (2 weeks in			< 55 dB: reference<br>55.0-59.9 dB: HR=0.80 (0.50-1.27)<br>60.0-64.9 dB: HR=0.75 (95%CI 0.47-1.19)<br>≥ 65 dB: HR=0.77 (95% CI 0.48-1.23)<br><br>L_{DEN}<br><br>< 55 dB: reference<br>55.0-59.9 dB: HR=1.01 (0.87-1.16)<br>60.0-64.9 dB: HR=1.09 (95%CI 0.90-1.32)<br>≥ 65 dB: HR=0.92 (95% CI 0.56-1.53)	comorbidity, socioeconomic deprivation, neighbourhood walkability, and time period<br><br>strengths, weaknesses:<br>-chronology between exposition and outcome not given<br>- noise summarized: road traffic main predictor, vicinity to aircraft and railway

Reference (First author, publication year, S/N) Meta-analysis	Study design	Population: Study region Sample population Sample size (M, F, M+F): Age (mean, range) No. of cases / no. of controls or exposed/unexposed Time of recruitment / follow-up (mean, range) Response (%) (baseline minus loss to follow-up)	Outcome: Disease (ICD-10) Prescription Questionnaire	Exposure: exposure source	Exposure: exposure assessment	Exposure: exposure levels	Results	Comments (study quality [overall assessment according to SIGN/CASP], conflict of interest [stated vs. not stated], funding [financed from public funds vs. financed from industry], confounding, strengths / weaknesses [potential bias, over- or underestimation of potential effects])
		No. of cases/ controls<br>Depression: n=959<br>No mental disorder: n=137,562<br><br>Time of recruitment<br>Baseline: 2000-2016<br>Follow-up: Delivery – 2017<br><br>Response: not applicable routine data-		summer) and in 2014 (5 weeks in spring)				traffic additional predictors<br>- noise and social economic information only available at neighborhood level<br>-info on moving missing, linkage of addresses<br><br>+ adequate definition and assessment of outcome<br>+adequate control for confounders (air particulates were

Reference (First author, publication year, S/N) Meta-analysis	Study design	Population: Study region Sample population Sample size (M, F, M+F): Age (mean, range) No. of cases / no. of controls or exposed/unexposed Time of recruitment / follow-up (mean, range) Response (%) (baseline minus loss to follow-up)	Outcome: Disease (ICD-10) Prescription Questionnaire	Exposure: exposure source	Exposure: exposure assessment	Exposure: exposure levels	Results	Comments (study quality [overall assessment according to SIGN/CASP], conflict of interest [stated vs. not stated], funding [financed from public funds vs. financed from industry], confounding, strengths / weaknesses [potential bias, over- or underestimation of potential effects])
								considered in a sensitivity analysis)<br>+ adequate exposure assessment<br>+ routine data, should lead to reduced selection bias
Jonah, Brian A., 1981, 1182<br>Canada<br>No (continuous	Cross-sectional study	Study region:<br>southern Ontario, Canada<br><br>Sample size:<br>M+F=1150<br><br>Age:<br>Not reported	Anxiety by Spielberger's measure of trait anxiety as secondary outcome	Road traffic noise	$L_{eq,24h}$ calculated	Continuous analysis<br><br>45-75 dB	"The relationship between traffic noise and anxiety was weak, $r = .06$, $p < .05$" (Table 1)	Study quality<br><br>- (to - -)<br><br>Conflict of interest<br>None disclosed<br><br>Funding<br>Not mentioned

Reference (First author, publication year, S/N) Meta-analysis	Study design	Population: Study region<br>Sample population<br>Sample size (M, F, M+F):<br>Age (mean, range)<br>No. of cases / no. of controls or exposed/unexposed<br>Time of recruitment / follow-up (mean, range)<br>Response (%)<br>(baseline minus loss to follow-up)	Outcome: Disease (ICD-10)<br>Prescription<br>Questionnaire	Exposure: exposure source	Exposure: exposure assessment	Exposure: exposure levels	Results	Comments (study quality [overall assessment according to SIGN/CASP], conflict of interest [stated vs. not stated], funding [financed from public funds vs. financed from industry], confounding, strengths / weaknesses [potential bias, over- or underestimation of potential effects])
variables, outcome not OR/RR)		Exposed/unexposed:<br>NA<br><br>Time of recruitment/Follow-up:<br>14-month period, but not specified when<br><br>Response:<br>Not reported						Confounding<br>Not adjusted<br><br>Strengths/ weaknesses:<br>+ Good noise assessment<br>+ outcome measurement of good internal consistency, validity and reliability<br>- No response %<br>- No adjustment for confounders between road traffic and

Reference (First author, publication year, S/N) Meta-analysis	Study design	Population<br>Study region<br>Sample population<br>Sample size (M, F, M+F):<br>Age (mean, range)<br>No. of cases / no. of controls or exposed/unexposed<br>Time of recruitment / follow-up (mean, range)<br>Response (%)<br>(baseline minus loss to follow-up)	Outcome<br>Disease (ICD-10)<br>Prescription<br>Questionnaire	Exposure<br>exposure source	exposure assessment	exposure levels	Results	Comments (study quality [overall assessment according to SIGN/CASP], conflict of interest [stated vs. not stated], funding [financed from public funds vs. financed from industry], confounding, strengths / weaknesses [potential bias, over- or underestimation of potential effects])
								anxiety<br>- cross-sectional study<br>- Sampling method not given in detail<br>- characteristics of respondents not given<br>- No other sources of noise considered, although "traffic noise only type of noise to which residents were exposed"<br>- Selection bias possible: survey sites represented maximum range of

Reference (First author, publication year, S/N) Meta-analysis	Study design	Population<br>Study region<br>Sample population<br>Sample size (M, F, M+F):<br>Age (mean, range)<br>No. of cases / no. of controls or exposed/unexposed<br>Time of recruitment / follow-up (mean, range)<br>Response (%)<br>(baseline minus loss to follow-up)	Outcome<br>Disease (ICD-10)<br>Prescription<br>Questionnaire	Exposure<br>exposure source	exposure assessment	exposure levels	Results	Comments (study quality [overall assessment according to SIGN/CASP], conflict of interest [stated vs. not stated], funding [financed from public funds vs. financed from industry], confounding, strengths / weaknesses [potential bias, over- or underestimation of potential effects])
								traffic noise level that could be found (45-75 dBA)<br>- air particulate level not considered
Klompmaker, 2019, 8746	Cross-sectional<br>National	Study region: Netherlands<br><br>Sample size: M+F=354,827<br>M= 161,045, F=193,782	Kessler psychological distress scale (K10) using ≥30 to define psych. distress in the	Road 2011<br><br>Railway 2011	Modelled -> Standard Model Instrumentation for Noise Assessments	-	Prevalence<br>*Self-reported psychological distress* (severe): n = 15,656 (4.7%)<br>*Prescriptions*<br>Anxiolytics (N05B): n = 7165 (2.0%)<br>Hypnotics and sedatives (N505C): n =	study quality: +<br>Cross-sectional analysis<br>conflict of interest: stated

Reference (First author, publication year, S/N) Meta-analysis	Study design	Population: Study region, Sample population, Sample size (M, F, M+F): Age (mean, range), No. of cases / no. of controls or exposed/unexposed, Time of recruitment / follow-up (mean, range), Response (%) (baseline minus loss to follow-up)	Outcome: Disease (ICD-10), Prescription, Questionnaire	Exposure: exposure source	Exposure: exposure assessment	Exposure: exposure levels	Results	Comments (study quality [overall assessment according to SIGN/CASP], conflict of interest [stated vs. not stated], funding [financed from public funds vs. financed from industry], confounding, strengths / weaknesses [potential bias, over- or underestimation of potential effects])
	Health Survey with secondary data on prescription medications	Sample population:<br>Sample based on data from national health survey (Public Health Monitor)<br><br>Age:<br>19-39 years: n = 68,940<br>40-64 years: n = 134,161<br>≥65 years: n = 151,726 (≥65 oversampled as part of study design)<br><br>No. of cases<br>See results	past 30 days<br><br>Prescriptions in 2012 (ATC code)<br>Anxiolytics (N05B)<br>Hypnotics & sedatives (N05C)<br>Antidepressants (N06A)		(STAMINA)<br>spatial resolution varied between 10x10m (close to source) to 80x80m<br><br>L_{DEN}		4346 (1.2%)<br>Antidepressants: n = 25,748 (7.3%)<br><br>Road traffic noise per 7.5 dB (IQR):<br>Psychological distress: OR = 1.00 (95% CI 0.98-1.03)<br>Anxiolytics: OR = 1.07 (95% CI 1.03-1.11)<br>Hypnotics and sedatives: OR = 1.01 (95% CI 0.97-1.06)<br>Antidepressants: OR = 0.99 (95% CI 0.97-1.01)<br><br>*Models adjusted for air pollution*<br>NO_2 -> lower risk estimates (all non-significant)	funding: stated<br>confounding (controlled for): sex, age, marital status, region of origin, education, paid occupation, household income, neighborhood SES, smoking status, alcohol use and degree of urbanization<br>strengths, weaknesses:

Reference (First author, publication year, S/N) Meta-analysis	Study design	Population: Study region Sample population Sample size (M, F, M+F): Age (mean, range) No. of cases / no. of controls or exposed/unexposed Time of recruitment / follow-up (mean, range) Response (%) (baseline minus loss to follow-up)	Outcome: Disease (ICD-10) Prescription Questionnaire	Exposure: exposure source	Exposure: exposure assessment	Exposure: exposure levels	Results	Comments (study quality [overall assessment according to SIGN/CASP], conflict of interest [stated vs. not stated], funding [financed from public funds vs. financed from industry], confounding, strengths / weaknesses [potential bias, over- or underestimation of potential effects])
		Time of recruitment 2012<br><br>Response: 47%					$PM_{2.5}$ -> similar risk estimates (nearly all significant)<br><br>Railway noise per 8.9 dB (IQR):<br>Psychological distress: OR = 1.04 (95% CI 1.02-1.06)<br>Anxiolytics: OR = 1.01 (95% CI 0.98-1.04)<br>Hypnotics and sedatives: OR = 0.99 (95% CI 0.95-1.03)<br>Antidepressants: OR = 0.99 (95% CI 0.97-1.00)<br><br>*Models adjusted for air pollution*<br>NO_2 -> similar risk estimates (psychological distress)	-Cross-sectional analysis within longitudinal study, with basic information about chronology between exposition and outcome<br><br>+ adequate definition and assessment of outcome<br>+adequate control for confounders (including consideration of air

Reference (First author, publication year, S/N) Meta-analysis	Study design	Population: Study region Sample population Sample size (M, F, M+F): Age (mean, range) No. of cases / no. of controls or exposed/unexposed Time of recruitment / follow-up (mean, range) Response (%) (baseline minus loss to follow-up)	Outcome: Disease (ICD-10) Prescription Questionnaire	Exposure: exposure source	Exposure: exposure assessment	Exposure: exposure levels	Results	Comments (study quality [overall assessment according to SIGN/CASP], conflict of interest [stated vs. not stated], funding [financed from public funds vs. financed from industry], confounding, strengths / weaknesses [potential bias, over- or underestimation of potential effects])
							$PM_{2.5}$ -> higher risk estimates (all significant)	particulate levels)<br>+ adequate exposure assessment
Leijssen, 2019, 8650	Cross-sectional<br><br>HELIUS study (Healthy Life in an	Study region: Amsterdam (Netherlands)<br><br>Sample size: M+F= 23,293 M= 9,920, F= 13,373<br><br>Sample population: Random sample drawn from municipal population register	Outcome: depressed mood<br><br>Patient Health Questionnaire (PHQ-9)	road traffic noise<br><br>data from 2011	$L_{eq,24h}$	45-54 dB<br>55-59 dB<br>60-64 dB<br>65-69 dB<br>≥ 70 dB	Table 3: OR noise and depressed mood (Model C)<br>dB / % / OR / 95% CI:<br>45-54 / 30.9 / 1 /<br>55-59 / 46.6 / 0.94 / 0.84-1.06<br>60-64 / 16.4 / 0.82 / 0.70-0.97<br>65-69 / 4.7 / 1.07 / 0.85-1.36<br>≥ 70 / 1.2 / 1.65 / 1.10-2.48	study quality: -(to +)<br>cross-sectional analysis<br>conflict of interest: stated (none)<br>funding: stated (by Academic Medical Center Amsterdam, Public Health Service of

Reference (First author, publication year, S/N) Meta-analysis	Study design	Population: Study region Sample population Sample size (M, F, M+F): Age (mean, range) No. of cases / no. of controls or exposed/unexposed Time of recruitment / follow-up (mean, range) Response (%) (baseline minus loss to follow-up)	Outcome: Disease (ICD-10) Prescription Questionnaire	Exposure: exposure source	Exposure: exposure assessment	Exposure: exposure levels	Results	Comments (study quality [overall assessment according to SIGN/CASP], conflict of interest [stated vs. not stated], funding [financed from public funds vs. financed from industry], confounding, strengths / weaknesses [potential bias, over- or underestimation of potential effects])
	Urban Setting)	Age:<br>Range: 18-70 years<br>Mean: 44 years<br>18-29 years: n=4,425<br>30-39 years: n=4,215<br>40-49 years: n=5,676<br>50-59 years: n=6,018<br>≥ 60 years: n=2,959<br><br>Time of recruitment:<br>2011-2015<br><br>Response:<br>N=49,952 invited -> n=24,789 (participated) -> 49.6%<br>n=23,293 (full data) -> 46.6%					Different sensitivity analyses performed for noise exposure ≥ 65 dB and depressive mood for: ethnicity, educational status, occupational status, age and sex.<br>(results not extracted)	Amsterdam, Dutch Hearth Foundation, Netherlands Organization for Health Research and Development, European Union, European Fund for the Integration of non-EU immigrants)<br>confounding (controlled for):<br>age, sex, ethnic origin, educational level, occupational status, marital status,

Reference (First author, publication year, S/N) Meta-analysis	Study design	Population: Study region Sample population Sample size (M, F, M+F): Age (mean, range) No. of cases / no. of controls or exposed/unexposed Time of recruitment / follow-up (mean, range) Response (%) (baseline minus loss to follow-up)	Outcome: Disease (ICD-10) Prescription Questionnaire	Exposure: exposure source	Exposure: exposure assessment	Exposure: exposure levels	Results	Comments (study quality [overall assessment according to SIGN/CASP], conflict of interest [stated vs. not stated], funding [financed from public funds vs. financed from industry], confounding, strengths / weaknesses [potential bias, over- or underestimation of potential effects])
								household composition, neuroticism, stressful life events, socioeconomic status, blue/ green space and liveability strengths, weaknesses: -Cross-sectional study, with basic information about chronology between exposition and outcome + random sample

Reference (First author, publication year, S/N) Meta-analysis	Study design	Population: Study region, Sample population, Sample size (M, F, M+F): Age (mean, range), No. of cases / no. of controls or exposed/unexposed, Time of recruitment / follow-up (mean, range), Response (%) (baseline minus loss to follow-up)	Outcome: Disease (ICD-10), Prescription, Questionnaire	Exposure: exposure source	Exposure: exposure assessment	Exposure: exposure levels	Results	Comments (study quality [overall assessment according to SIGN/CASP], conflict of interest [stated vs. not stated], funding [financed from public funds vs. financed from industry], confounding, strengths / weaknesses [potential bias, over- or underestimation of potential effects])
								+ adequate definition and assessment of outcome<br>+adequate control for confounders<br>+ adequate exposure assessment<br>+ high statistical power<br>+ ethics approval (by Institutional Review Board of the Academic Medical Center, University of Amsterdam)
Lercher	Cross-	Study region:	Tranquilizers	Road	L_{eq}	<55 dB	Table 1: Noise level and prescriptions	study quality:

Reference (First author, publication year, S/N) Meta-analysis	Study design	Population: Study region, Sample population, Sample size (M, F, M+F): Age (mean, range), No. of cases / no. of controls or exposed/unexposed, Time of recruitment / follow-up (mean, range), Response (%) (baseline minus loss to follow-up)	Outcome: Disease (ICD-10), Prescription, Questionnaire	Exposure: exposure source	Exposure: exposure assessment	Exposure: exposure levels	Results	Comments (study quality [overall assessment according to SIGN/CASP], conflict of interest [stated vs. not stated], funding [financed from public funds vs. financed from industry], confounding, strengths / weaknesses [potential bias, over- or underestimation of potential effects])
, P. 1996, H-2891, Austria<br>-	sectional study	5 rural communities along 2 major traffic routes in the Austrian part of the Alps<br><br>Sample population:<br>No information about sampling<br><br>Sample size:<br>M+F=1989<br><br>Age:<br>25-65 years<br><br>Exposed/unexposed:<br>NA	Self-reported prescriptions (questionnaire)<br><br>Five-grade frequency response scale: <once/month, once/month, once/fortnight, once/week, daily	traffic	(long-term and short-term measurements)	(Reference),<br>>55 dB	(OR 95% CI)<br>Tranquilizer: 1.13 (0.60-2.13)	- (to --)<br>Cross-sectional study design<br><br>conflict of interest:<br>not stated<br><br>funding:<br>not stated<br><br>confounding:<br>adjusted for age, sex and education<br><br>strengths, weaknesses:

Reference (First author, publication year, S/N) Meta-analysis	Study design	Population: Study region Sample population Sample size (M, F, M+F): Age (mean, range) No. of cases / no. of controls or exposed/unexposed Time of recruitment / follow-up (mean, range) Response (%) (baseline minus loss to follow-up)	Outcome: Disease (ICD-10) Prescription Questionnaire	Exposure: exposure source	Exposure: exposure assessment	Exposure: exposure levels	Results	Comments (study quality [overall assessment according to SIGN/CASP], conflict of interest [stated vs. not stated], funding [financed from public funds vs. financed from industry], confounding, strengths / weaknesses [potential bias, over- or underestimation of potential effects])
		Time of recruitment/ follow-up: not reported<br><br>Response: 62%						+even response across all noise classes, participants in noise classes comparable in many aspect (except participants in classes >55 dB more likely to have rented home and be younger (1.2 years)); exposed did not differ in potential confounders<br>- unclear exposure measurement, or which times it covered

Reference (First author, publication year, S/N) Meta-analysis	Study design	Population: Study region Sample population Sample size (M, F, M+F): Age (mean, range) No. of cases / no. of controls or exposed/unexposed Time of recruitment / follow-up (mean, range) Response (%) (baseline minus loss to follow-up)	Outcome: Disease (ICD-10) Prescription Questionnaire	Exposure: exposure source	Exposure: exposure assessment	Exposure: exposure levels	Results	Comments (study quality [overall assessment according to SIGN/CASP], conflict of interest [stated vs. not stated], funding [financed from public funds vs. financed from industry], confounding, strengths / weaknesses [potential bias, over- or underestimation of potential effects])
								- no information on sampling<br>-cross-sectional<br>-missing info about participant numbers in general categories and noise classes<br>-self-reported drug prescriptions as outcome<br>- no consideration of other important confounders (i.e. air pollution)<br>- no other noise exposure considered

Reference (First author, publication year, S/N) Meta-analysis	Study design	Population: Study region, Sample population, Sample size (M, F, M+F): Age (mean, range), No. of cases / no. of controls or exposed/unexposed, Time of recruitment / follow-up (mean, range), Response (%) (baseline minus loss to follow-up)	Outcome: Disease (ICD-10), Prescription, Questionnaire	Exposure: exposure source	Exposure: exposure assessment	Exposure: exposure levels	Results	Comments (study quality [overall assessment according to SIGN/CASP], conflict of interest [stated vs. not stated], funding [financed from public funds vs. financed from industry], confounding, strengths / weaknesses [potential bias, over- or underestimation of potential effects])
Linares, C., 2017, 1515, Spain<br>-	Ecological time-series study	Study region:<br>Madrid<br><br>Sample population:<br>Population of Madrid<br><br>Sample size:<br>M+F=3,116,897 (Madrid population)<br>M+F= 754,005 (≥ 60 years)<br><br>Age:<br>No information	ICD-9: hospital admissions:<br>290: Dementias (290.0-290.2, 290.4-290.9)<br><br>294.1-294: Persistent mental disorders due to conditions classified elsewhere	Road traffic	L_D (08-22h)<br>L_N (22-08h)<br><br>Collected from 27 urban stations spread across Madrid<br><br>Year 2009?<br><br>($PM_{2.5}$, PM_{10}, O_3, NO_2 assessed)	Continuous<br><br>$L_{eq,D}$:<br>Range: 59.4 (Min)-69 (Max)<br><br>L_N:<br>55.0-67.2 dB	Linear relationship between $L_{eq,D}$ and number of daily dementia-related emergency (DDE): R = 0.918, p < 0.001<br><br>Calculation of RR for 1dB increase in L_D<br>RR for 1 dB(A) increase in L_D (from Table 2)<br><br>RR (95% CI)<br>Daily Dementia-re … Tcal (lag 1): 1.19 (1.09-1.30)	study quality:<br>- (to --)<br><br>conflict of interest:<br>not stated<br><br>funding:<br>Miguel Servet type 1 grant, FIS Project ENPY<br><br>confounding (controlled):<br>time trend,

		Population	Outcome	Exposure					
**Reference (First author, publication year, S/N) Meta-analysis**	**Study design**	**Study region Sample population Sample size (M, F, M+F): Age (mean, range) No. of cases / no. of controls or exposed/unexposed Time of recruitment / follow-up (mean, range) Response (%) (baseline minus loss to follow-up)**	**Disease (ICD-10) Prescription Questionnaire**	**exposure source**	**exposure assessment**	**exposure levels**	**Results**		**Comments (study quality [overall assessment according to SIGN/CASP], conflict of interest [stated vs. not stated], funding [financed from public funds vs. financed from industry], confounding, strengths / weaknesses [potential bias, over- or underestimation of potential effects])**
		No of cases/ no of controls: n = 3,287 (daily dementia-related hospital admissions, Table 1)<br>n= 1,175 dementia admissions (Abstract/ Result text)<br><br>Time of recruitment/ follow-up: January 2001 to December 31, 2009<br><br>Response: No information					lated hospital admissions	Teqd (lag 0): 1.15 (1.11-1.20)<br>O3a (lag 5): 1.09 (1.04-1.15)	seasonality, autoregression, day of the week<br><br>strengths, weaknesses:<br>-ecological study<br>- very rough estimation of exposure and very little variation in mean noise exposure levels<br>-Missing information on population and characteristics

Reference (First author, publication year, S/N) Meta-analysis	Study design	Population: Study region Sample population Sample size (M, F, M+F): Age (mean, range) No. of cases / no. of controls or exposed/unexposed Time of recruitment / follow-up (mean, range) Response (%) (baseline minus loss to follow-up)	Outcome: Disease (ICD-10) Prescription Questionnaire	Exposure: exposure source	Exposure: exposure assessment	Exposure: exposure levels	Results	Comments (study quality [overall assessment according to SIGN/CASP], conflict of interest [stated vs. not stated], funding [financed from public funds vs. financed from industry], confounding, strengths / weaknesses [potential bias, over- or underestimation of potential effects])
								- Dementia-related hospital admissions -> does not reflect the prevalence of dementia in Madrid<br>- linkage between noise and hospital admissions<br>- possible under-recording of emergencies<br>- no other noise exposure considered
Meecham &	Cross-sectio	Study region:<br>Vicinity of Los Angeles	Mental hospital admissions	Aircraft noise	MNA (maximum		Mental health admissions for eight months in 1971	Study quality<br>- (to --)

Reference (First author, publication year, S/N) Meta-analysis	Study design	Population: Study region; Sample population; Sample size (M, F, M+F); Age (mean, range); No. of cases / no. of controls or exposed/unexposed; Time of recruitment / follow-up (mean, range); Response (%) (baseline minus loss to follow-up)	Outcome: Disease (ICD-10) Prescription Questionnaire	Exposure: exposure source	Exposure: exposure assessment	Exposure: exposure levels	Results	Comments (study quality [overall assessment according to SIGN/CASP], conflict of interest [stated vs. not stated], funding [financed from public funds vs. financed from industry], confounding, strengths / weaknesses [potential bias, over- or underestimation of potential effects])
Smith, 1977, 1679<br>USA<br>-	nal study	Airport<br><br>Study population<br>Population in MNA compared to population in control area (nearby city of El Segundo with noise levels below 90dBA levels of MNA)<br><br>Sample size:<br>M+F= 137,331<br><br>Age<br>Median age unexposed area= 32 years<br>Median age MNA area=	in South Bay Mental Health Service		noise area) 90 dB and higher<br><br>Determined with census tract map		MNA / Control<br>Total number: 76 / 32<br>Number/ 100,000-yr: 128 / 99<br>p-value chi-sq test=0.10<br><br>29% increase in admissions in MNA area	Conflict of interest<br>Not stated<br><br>Funding<br>Not stated<br><br>Confounding<br>Apparently no adjustment for confounders<br><br>Strengths/ weaknesses:<br>+ large sample size<br>- No adjustment for

Reference (First author, publication year, S/N) Meta-analysis	Study design	Population<br>Study region<br>Sample population<br>Sample size (M, F, M+F):<br>Age (mean, range)<br>No. of cases / no. of controls or exposed/unexposed<br>Time of recruitment / follow-up (mean, range)<br>Response (%)<br>(baseline minus loss to follow-up)	Outcome<br>Disease (ICD-10)<br>Prescription<br>Questionnaire	Exposure<br>exposure source	exposure assessment	exposure levels	Results	Comments<br>(study quality [overall assessment according to SIGN/CASP], conflict of interest [stated vs. not stated], funding [financed from public funds vs. financed from industry], confounding, strengths / weaknesses [potential bias, over- or underestimation of potential effects])
		27 years<br><br>Exposed/Unexposed<br>In control area<br>M+F=48,330<br>In exposed area<br>M+F=89,001<br><br>Time of recruitment:<br>8 months in 1971<br><br>Response (%)<br>NA						confounders, except indirectly for SES (two areas were chosen for equal SES)<br>- all covariates, including noise exposure, ecological measurements<br>- Temporal relationship cannot be established<br>- low income area (generalizability)<br>- potential underestimation of the effect: "In cases

Reference (First author, publication year, S/N) Meta-analysis	Study design	Population: Study region Sample population Sample size (M, F, M+F): Age (mean, range) No. of cases / no. of controls or exposed/unexposed Time of recruitment / follow-up (mean, range) Response (%) (baseline minus loss to follow-up)	Outcome: Disease (ICD-10) Prescription Questionnaire	Exposure: exposure source	Exposure: exposure assessment	Exposure: exposure levels	Results	Comments (study quality [overall assessment according to SIGN/CASP], conflict of interest [stated vs. not stated], funding [financed from public funds vs. financed from industry], confounding, strengths / weaknesses [potential bias, over- or underestimation of potential effects])
								where the contour did not fully enclose the tract, we nevertheless used the population of the entire tract,…"] - no other noise exposure considered - air particulate level not considered
Miyakawa, M. 2007, H-2884, Japan No (no	Cross-sectional study	Study region: Narita International airport, Japan<br><br>Sample population: all residents using a leave and	Psychiatric disorder measured by GHQ-28 Japanese version, cut off 6 = having	Aircraft	L_{DEN} Exposure measurement from 2001	Exposed: range: 55-65 dB,<br><br>2 subgrou	ORs for psychiatric disorders for L_{DEN} (reference category: control group) (personal communication) 55-59 dB(A): OR: 1.79 (95 % CI: 0.837-3.85) 59-65 dB(A): OR: 2.22 (95 % CI:	study quality: - Cross-sectional study design<br><br>conflict of interest:

Reference (First author, publication year, S/N) Meta-analysis	Study design	Population<br>Study region<br>Sample population<br>Sample size (M, F, M+F):<br>Age (mean, range)<br>No. of cases / no. of controls or exposed/unexposed<br>Time of recruitment / follow-up (mean, range)<br>Response (%)<br>(baseline minus loss to follow-up)	Outcome<br>Disease (ICD-10)<br>Prescription<br>Questionnaire	Exposure<br>exposure source	exposure assessment	exposure levels	Results	Comments (study quality [overall assessment according to SIGN/CASP], conflict of interest [stated vs. not stated], funding [financed from public funds vs. financed from industry], confounding, strengths / weaknesses [potential bias, over- or underestimation of potential effects])
exposure for control group)		pick up method<br><br>Sample size:<br>M+F= 188<br>M= 101, F= 87<br><br>Age:<br>20-39 years: 35<br>40-59 years: 102<br>60-79 years: 51<br><br>No. of cases/ no. of controls:<br>Exposed:<br>Subsample M+F= 113<br>(M=61, F= 52)<br>two groups of the subsample:	a psychiatric disorder			ps:<br>55-59 dB,<br>59-65 dB	0.989-4.97)	not stated<br><br>funding:<br>stated (Grand-in Aid)→ public funding<br><br>confounding (adjusted for):<br>sex, age, occupation of householder, interaction between sex and age<br><br>strengths, weaknesses:<br>+ multiple logistic

Referen ce (First author, publica tion year, S/N) Meta-a nalysis	Study desig n	Population<br>Study region<br>Sample population<br>Sample size (M, F, M+F):<br>Age (mean, range)<br>No. of cases / no. of controls or exposed/unexposed<br>Time of recruitment / follow-up (mean, range)<br>Response (%)<br>(baseline minus loss to follow-up)	Outcome<br>Disease (ICD-10)<br>Prescription<br>Questionnaire	Exposure<br>exposure source	exposure assessment	exposure levels	Results	Comments<br>(study quality [overall assessment according to SIGN/CASP], conflict of interest [stated vs. not stated], funding [financed from public funds vs. financed from industry], confounding, strengths / weaknesses [potential bias, over- or underestimation of potential effects])
		55-59 dB: M+F=62 (M=37; F=25)<br>59-65 dB: M+F=51 (M: 24; F: 27)<br>Not exposed:<br>M+F= 75 (M= 40; F: 35)<br><br>Time of recruitment/ follow-up:<br>2003<br><br>Response:<br>58.2 %<br>Valid response. 37.6% (N=188/500)						regression models<br>+ exposure measurement instrument<br>+ exposure measurement before survey → chronology<br>+ valid outcome measurement<br>- cross-sectional design<br>- only occupation of householder not socioeconomic status mentioned<br>- no noise value for

Reference (First author, publication year, S/N) Meta-analysis	Study design	Population: Study region, Sample population, Sample size (M, F, M+F): Age (mean, range), No. of cases / no. of controls or exposed/unexposed, Time of recruitment / follow-up (mean, range), Response (%) (baseline minus loss to follow-up)	Outcome: Disease (ICD-10) Prescription Questionnaire	Exposure: exposure source	Exposure: exposure assessment	Exposure: exposure levels	Results	Comments (study quality [overall assessment according to SIGN/CASP], conflict of interest [stated vs. not stated], funding [financed from public funds vs. financed from industry], confounding, strengths / weaknesses [potential bias, over- or underestimation of potential effects])
								control group given<br>- no other noise exposure considered<br>- air particulate level not considered
Nivison, 1993, 1837, Norway<br>No (only correlation	Cross sectional study	Study region:<br>Norway, city/area not specified<br><br>Sample size:<br>(94 → 12 excluded due to hearing impairments)<br>M+F= 82<br>M= 35, F= 47	Anxiety by Spielberger's Trait Anxiety Inventory	Road traffic noise	$L_{eq, 24h}$<br>L_{max}<br><br>3rd variable formed by combining L_{eq} with number of hours each person reported	Continuous<br>$L_{eq, 24h}$: mean 69 dB, range: 63-72 dB<br>L_{max}: mean	Table 1 shows nervous symptoms and $L_{eq, 24h}$, L_{max}, Level x exposure, but only variables with significant F values for R2 change shown.<br>Not significant crude association between nervous symptoms and all three noise exposures (for men and women)	Study quality<br>- (to - -)<br><br>Conflict of interest<br>Not stated<br><br>Funding<br>Public<br>Norwegian National

Reference (First author, publication year, S/N) Meta-analysis	Study design	Population: Study region, Sample population, Sample size (M, F, M+F): Age (mean, range), No. of cases / no. of controls or exposed/unexposed, Time of recruitment / follow-up (mean, range), Response (%) (baseline minus loss to follow-up)	Outcome: Disease (ICD-10), Prescription, Questionnaire	Exposure: exposure source	Exposure: exposure assessment	Exposure: exposure levels	Results	Comments (study quality [overall assessment according to SIGN/CASP], conflict of interest [stated vs. not stated], funding [financed from public funds vs. financed from industry], confounding, strengths / weaknesses [potential bias, over- or underestimation of potential effects])
coefficients reported, only significant terms reported)		Age<br>Mean:<br>men: 36.1 years<br>women: 44.1 years<br>Range: 19-78 years<br><br>Exposed/unexposed<br>N/A<br><br>Time of recruitment:<br>Not mentioned<br><br>Response (%)<br>56%			spending at home during week (noise exposure/person)	87.5 dB, range: 80-91 dB	Table 2 shows anxiety and L_{eq}, L_{max}, Level x exposure, but only significant results shown.<br>Anxiety to Level x exposure correlation coeff: 0.32 p≤0.05 for men (not significant for women)<br>Anxiety to L_{eq} and L_{max} not significant (for men and women)<br><br>Note: The primary outcome was not anxiety, and so the relationship between anxiety and noise exposure was not explored further.	Pollution Agency, the Nordic Noise Group, and the Norwegian Research Council for Science and Humanities<br><br>Confounding<br>Some controlled for age<br><br>Strengths/weaknesses:<br>+ good exposure assessment<br>- sampling/recruiting

Reference (First author, publication year, S/N) Meta-analysis	Study design	Population: Study region Sample population Sample size (M, F, M+F): Age (mean, range) No. of cases / no. of controls or exposed/unexposed Time of recruitment / follow-up (mean, range) Response (%) (baseline minus loss to follow-up)	Outcome: Disease (ICD-10) Prescription Questionnaire	Exposure: exposure source	Exposure: exposure assessment	Exposure: exposure levels	Results	Comments (study quality [overall assessment according to SIGN/CASP], conflict of interest [stated vs. not stated], funding [financed from public funds vs. financed from industry], confounding, strengths / weaknesses [potential bias, over- or underestimation of potential effects])
								not described- potential selection bias<br>- population characteristics not described<br>- only significant values shown for some tables<br>- No power calculation<br>- cross-sectional design<br>- older persons more often declined to take part in the study than

Reference (First author, publication year, S/N) Meta-analysis	Study design	Population: Study region Sample population Sample size (M, F, M+F): Age (mean, range) No. of cases / no. of controls or exposed/unexposed Time of recruitment / follow-up (mean, range) Response (%) (baseline minus loss to follow-up)	Outcome: Disease (ICD-10) Prescription Questionnaire	Exposure: exposure source	Exposure: exposure assessment	Exposure: exposure levels	Results	Comments (study quality [overall assessment according to SIGN/CASP], conflict of interest [stated vs. not stated], funding [financed from public funds vs. financed from industry], confounding, strengths / weaknesses [potential bias, over- or underestimation of potential effects])
								younger persons (selection bias) Underestimation of potential effects: younger population with better health - no other noise exposure considered - air particulate level not considered
Okonen, 2018, 8461	Cross-sectional study (Helsi	Study region: Helsinki, Espoo, Vantaa (Finland)<br><br>Sample size:	„survey questionnaire“ (no literature) 1 question about intake of sleeping	Road traffic noise<br><br>Data from	L_{DEN}	≤45 dB, 45.1–50 dB, 50.1–55 dB, 55.1–60	Table 2: OR modelled noise and sleep medication (adjusted)<br>dB / n / OR / 95% CI<br>≤45 dB / 119 / 1 /	study quality: - Cross-sectional analysis conflict of interest:

Reference (First author, publication year, S/N) Meta-analysis	Study design	Population: Study region<br>Sample population<br>Sample size (M, F, M+F):<br>Age (mean, range)<br>No. of cases / no. of controls or exposed/unexposed<br>Time of recruitment / follow-up (mean, range)<br>Response (%)<br>(baseline minus loss to follow-up)	Outcome: Disease (ICD-10)<br>Prescription<br>Questionnaire	Exposure: exposure source	Exposure: exposure assessment	Exposure: exposure levels	Results	Comments (study quality [overall assessment according to SIGN/CASP], conflict of interest [stated vs. not stated], funding [financed from public funds vs. financed from industry], confounding, strengths / weaknesses [potential bias, over- or underestimation of potential effects])
	nki Capital Region Environmental Health Survey)	M+F= 7,321 (valid records)<br>M+F: 5,860 (included -> no missing data)<br>M= 2,497, F= 3,363<br><br>Sample population:<br>Random sample of population registry of Finland, adults>24 years<br><br>Age (mean)<br>Overall: 55.0 years<br>≤45 dB: 55.0 years (± 15.4)<br>45.1–50 dB: 54.5 years (± 15.6)<br>50.1–55 dB: 54.5 years (± 15.9)<br>55.1–60 dB: 54.4 years (± 16.5)	pills (proxy for sleep disorders), tranquilizers (proxy for anxiety disorders) and antidepressants (proxy for depression)<br>(past week, 1-4 weeks ago, 1-12 months ago, over a year ago, never)	2011		dB ≥60 dB<br><br>(sensitivity analysis: 60.1–65 dB and>65 dB)	45.1–50 | 194 | 1.17 | 0.87–1.45<br>50.1–55 | 182 | 0.99 | 0.77–1.28<br>55.1–60 | 168 | 1.06 | 0.81–1.37<br>≥60 | 174 | 0.97 | 0.75–1.26<br><br>Table 2: OR modelled noise and anxiolytics (adjusted)<br>dB | n | OR | 95% CI<br>≤45 dB | 48 | 1 | 45.1–50 | 82 | 1.12 | (0.77–1.63)	stated<br>funding:<br>not stated<br>confounding (controlled for):<br>sex, age, marital status, employment status, household income, alcohol intake, current smoking status, level of physical activity and pet ownership<br>"almost all respondents lived in urban areas, there was

Reference (First author, publication year, S/N) Meta-analysis	Study design	Population: Study region Sample population Sample size (M, F, M+F): Age (mean, range) No. of cases / no. of controls or exposed/unexposed Time of recruitment / follow-up (mean, range) Response (%) (baseline minus loss to follow-up)	Outcome: Disease (ICD-10) Prescription Questionnaire	Exposure: exposure source	Exposure: exposure assessment	Exposure: exposure levels	Results	Comments (study quality [overall assessment according to SIGN/CASP], conflict of interest [stated vs. not stated], funding [financed from public funds vs. financed from industry], confounding, strengths / weaknesses [potential bias, over- or underestimation of potential effects])
		≥60 dB: 53.5 years (± 16.6)<br><br>Time of recruitment 2015-2016<br><br>Response: N=16,000 47% in 2015 and 45% in 2016					(see tables below)	no justification to control for urbanity" Sensitivity analysis (test for effect modification) . noise annoyance, noise sensitivity, sleep disturbance from road-traffic noise, bedroom window orientation, BMI and presence of chronic disease strengths, weaknesses: -Cross-sectional

50.1–55	82	1.09	(0.75–1.58)
55.1–60	79	1.24	(0.85–1.82)
≥60	101	1.34	(0.93–1.93)

Table 2: OR modelled noise and antidepressants (adjusted)

dB	n	OR	95% CI
≤45 dB	49	1	
45.1–50	86	1.20	(0.83–1.73)
50.1–55	84	1.13	(0.78–1.64)

<table>
<tr><th rowspan="2">Referen ce (First author, publica tion year, S/N) Meta-a nalysis</th><th rowspan="2">Study desig n</th><th>Population</th><th>Outcome</th><th colspan="3">Exposure</th><th rowspan="2">Results</th><th rowspan="2">Comments (study quality [overall assessment according to SIGN/CASP], conflict of interest [stated vs. not stated], funding [financed from public funds vs. financed from industry], confounding, strengths / weaknesses [potential bias, over- or underestimation of potential effects])</th></tr>
<tr><th>Study region
Sample population
Sample size (M, F, M+F):
Age (mean, range)
No. of cases / no. of controls or exposed/unexposed
Time of recruitment / follow-up (mean, range)
Response (%)
(baseline minus loss to follow-up)</th><th>Disease (ICD-10)
Prescription
Questionnaire</th><th>exposure source</th><th>exposure assessment</th><th>exposure levels</th></tr>
<tr><td></td><td></td><td></td><td></td><td></td><td></td><td></td><td><table><tr><td>55.1–60</td><td>66</td><td>1.04</td><td>(0.70–1.53)</td></tr><tr><td>≥60</td><td>97</td><td>1.32</td><td>(0.91–1.90)</td></tr></table>
Sensitivity analysis: similar results (not extracted)</td><td>study, with basic information about chronology between exposition and outcome
- Funding not stated
- Anxiolytics may be indicative of sleep disorders (not differential to anxiety disorder)
- outcome evaluated with a single question about medication intake → as a proxy for the outcome (but</td></tr>
</table>

Reference (First author, publication year, S/N) Meta-analysis	Study design	Population: Study region Sample population Sample size (M, F, M+F): Age (mean, range) No. of cases / no. of controls or exposed/unexposed Time of recruitment / follow-up (mean, range) Response (%) (baseline minus loss to follow-up)	Outcome: Disease (ICD-10) Prescription Questionnaire	Exposure: exposure source	Exposure: exposure assessment	Exposure: exposure levels	Results	Comments (study quality [overall assessment according to SIGN/CASP], conflict of interest [stated vs. not stated], funding [financed from public funds vs. financed from industry], confounding, strengths / weaknesses [potential bias, over- or underestimation of potential effects])
								psychotropic medication is prescription-based in Finland, thus outcome represents a diagnosed condition requiring treatment)<br>- No information on questionnaire (, self-assessed, not validated?)<br>- Air pollution not considered as confounder<br>+ random selection of participants

Reference (First author, publication year, S/N) Meta-analysis	Study design	Population: Study region<br>Sample population<br>Sample size (M, F, M+F):<br>Age (mean, range)<br>No. of cases / no. of controls or exposed/unexposed<br>Time of recruitment / follow-up (mean, range)<br>Response (%)<br>(baseline minus loss to follow-up)	Outcome: Disease (ICD-10)<br>Prescription<br>Questionnaire	Exposure: exposure source	Exposure: exposure assessment	Exposure: exposure levels	Results	Comments (study quality [overall assessment according to SIGN/CASP], conflict of interest [stated vs. not stated], funding [financed from public funds vs. financed from industry], confounding, strengths / weaknesses [potential bias, over- or underestimation of potential effects])
								+adequate control for confounders<br>+ adequate exposure assessment<br>+ good statistical power
Orban, 2016, 1890<br>Yes	Prospective cohort study	Study region:<br>Bochum, Essen and Mülheim/Ruhr, Germany<br><br>Population sample:<br>Residents aged 45-75 years, randomly selected from population registries<br>(Heinz-Nixdorf Recall study)	Depression symptoms during the previous week<br>15 item CES-D<br><br>Antidepressive medication taken in previous 7 days	Road traffic noise	L_{DEN}<br>L_{N}<br><br>Noise exposure data was assigned to the geographic residence location of the study	High noise exposure L_{DEN}: >55 dB<br>vs<br>low noise exposure L_{DEN}: ≤55dB	Relative risks of high depressive symptoms at follow-up in association with exposure to different categories of 24 hour noise compared with the lowest noise category (Figure 2)<br><br>Noise dB / RR (95% CI) / n<br>≤55 (Ref) / 1.00 / 1,986	Study quality<br>+<br><br>Conflict of interest<br>No actual or potential competing financial interests declared<br><br>Funding<br>Public

Reference (First author, publication year, S/N) Meta-analysis	Study design	Population<br>Study region<br>Sample population<br>Sample size (M, F, M+F):<br>Age (mean, range)<br>No. of cases / no. of controls or exposed/unexposed<br>Time of recruitment / follow-up (mean, range)<br>Response (%)<br>(baseline minus loss to follow-up)	Outcome<br>Disease (ICD-10)<br>Prescription<br>Questionnaire	Exposure<br>exposure source	exposure assessment	exposure levels	Results	Comments<br>(study quality [overall assessment according to SIGN/CASP], conflict of interest [stated vs. not stated], funding [financed from public funds vs. financed from industry], confounding, strengths / weaknesses [potential bias, over- or underestimation of potential effects])
		Sample size:<br>M+F= 3300<br>M= 1715<br>F= 1585<br><br>Age:<br>L_{DEN}>55dB: 59.1±7.7 years<br>L_{DEN} ≤55dB: 59.3±7.6 years<br><br>Exposed/unexposed<br>See results,<br>At follow-up<br>35.7% (n=1,179) exposed to high 24h traffic (L_{DEN} >55dB)<br>25.8% (n=850) exposed to high nighttime noise levels	High depressive symptoms:<br>Intake of antidepressant medication classified in ATC groups N06 or N06CA and/or CES-D score ≥17<br>In the past week		participant at baseline using geo info system	(Reference)<br><br>L_{DEN} categories<br>≤55dB (Ref),<br>>55 to ≤60dB,<br>>60 to ≤65 dB,<br>>65 dB<br><br>High nighttim	>55 to ≤60 — 1.19 (0.86, 1.65) — 394<br>>60 to ≤65 — 1.52 (1.11, 2.07) — 353<br>>65 — 1.19 (0.85, 1.68) — 365<br><br>Relative risk of high depressive symptoms at follow-up in study participants exposed to residential traffic noise L_{DEN} >55dB and L_{DEN} ≤55dB (Table 2)<br>Model — RR — Cases — n_{tot}<br>Model 1<br>Total — 1.29 — 279 — 3,0[illegible]	Heinz Nixdorf Foundation, and Deutsche Forschungsgemeinschaft (DFG) and Kulturstiftung Essen, Germany<br><br>Confounding<br>Model 1: age, sex, education, income, economic activity, neighborhood level SES, and traffic proximity<br>Model 2: in addition,

Reference (First author, publication year, S/N) Meta-analysis	Study design	Population: Study region, Sample population, Sample size (M, F, M+F): Age (mean, range), No. of cases / no. of controls or exposed/unexposed, Time of recruitment / follow-up (mean, range), Response (%) (baseline minus loss to follow-up)	Outcome: Disease (ICD-10), Prescription, Questionnaire	Exposure: exposure source	Exposure: exposure assessment	Exposure: exposure levels	Results				Comments (study quality [overall assessment according to SIGN/CASP], conflict of interest [stated vs. not stated], funding [financed from public funds vs. financed from industry], confounding, strengths / weaknesses [potential bias, over- or underestimation of potential effects])
						e noise levels L_N: >50 dB		(1.03, 1.62)			BMI and smoking
		Time of recruitment/follow-up: Baseline: 2000-2003					Men	1.29 (0.87, 1.92)	98	1,650	Model 3: in addition, comorbidities and insomnia
		Follow-up: 2005-2008 Mean follow-up: 5.1 years					Women	1.30 (0.98, 1.72)	181	1,448	Strengths/weaknesses: + participants recruited from
		Response (%)					Model 2				
		In baseline survey: 55.8%					Total	1.28 (1.02, 1.61)	278	3,089	population registries + Good noise exposure assessment
		Loss to follow-up 12.9%					Men	1.28 (0.85, 1.94)	98	1,650	+ Longitudinal study, those with prevalent depressive symptoms
							Women	1.28	180	1,445	at baseline excluded

Reference (First author, publication year, S/N) Meta-analysis	Study design	Population: Study region, Sample population, Sample size (M, F, M+F): Age (mean, range), No. of cases / no. of controls or exposed/unexposed, Time of recruitment / follow-up (mean, range), Response (%) (baseline minus loss to follow-up)	Outcome: Disease (ICD-10), Prescription, Questionnaire	Exposure: exposure source	Exposure: exposure assessment	Exposure: exposure levels	Results	Comments (study quality [overall assessment according to SIGN/CASP], conflict of interest [stated vs. not stated], funding [financed from public funds vs. financed from industry], confounding, strengths / weaknesses [potential bias, over- or underestimation of potential effects])
							(0.97, 1.69)	+ high baseline response and low loss to follow-up
							Model 3	
							Total 1.26 (1.00, 1.58) 276 3,075	+ Confounder list pretty inclusive<br>- Self-reported
							Men 1.21 (0.81, 1.82) 97 1,637	antidepressant medication (participants were
							Women 1.28 (0.97, 1.70) 179 1,438	asked to bring medication)<br>- not adjusted for air particulate level
							Model 1 adjusted for age, sex, education, income, economic activity, neighborhood-level SES, traffic	- other sources of noise not considered<br>- Noise modeled for

Reference (First author, publication year, S/N) Meta-analysis	Study design	Population: Study region, Sample population, Sample size (M, F, M+F): Age (mean, range), No. of cases / no. of controls or exposed/unexposed, Time of recruitment / follow-up (mean, range), Response (%) (baseline minus loss to follow-up)	Outcome: Disease (ICD-10), Prescription, Questionnaire	Exposure: exposure source	Exposure: exposure assessment	Exposure: exposure levels	Results	Comments (study quality [overall assessment according to SIGN/CASP], conflict of interest [stated vs. not stated], funding [financed from public funds vs. financed from industry], confounding, strengths / weaknesses [potential bias, over- or underestimation of potential effects])
							proximity<br>Model 2 additionally adjusted for BMI, smoking<br>Model 3 additionally adjusted for comorbidities, insomnia<br><br>Relative risk of high depressive symptoms at follow-up in study participants exposed to residential night traffic noise L_N >50dB and L_N ≤50dB (Supplemental Material)<br>Model RR<br>Model 1<br>Total 1.29 (1.0 1, 1.64)<br>Men 1.19 (0.77, 1.82)	the year 2006, after baseline survey was done, and assumption of unchanged noise exposure during the study period may not hold<br>- no other noise exposure considered<br>- air particulate level not considered

Reference (First author, publication year, S/N) Meta-analysis	Study design	Population: Study region Sample population Sample size (M, F, M+F): Age (mean, range) No. of cases / no. of controls or exposed/unexposed Time of recruitment / follow-up (mean, range) Response (%) (baseline minus loss to follow-up)	Outcome: Disease (ICD-10) Prescription Questionnaire	Exposure: exposure source	Exposure: exposure assessment	Exposure: exposure levels	Results	Comments (study quality [overall assessment according to SIGN/CASP], conflict of interest [stated vs. not stated], funding [financed from public funds vs. financed from industry], confounding, strengths / weaknesses [potential bias, over- or underestimation of potential effects])
							Women 1.36 (1.01, 1.82)<br>Model 2<br>Total 1.30 (1.0 2, 1.65)<br>Men 1.19 (0.76, 1.86)<br>Women 1.37 (1.02, 1.83)<br>Model 3<br>Total 1.29 (1.01, 1.64)<br>Men 1.14 (0.74, 1.76)<br>Women 1.39 (1.03, 1.86)<br><br>Using Model 1, for L_{DEN}, <55dB=Ref (Fig. 2)<br>Noise level RR<br>>55 to ≤60 1.19 (0.86, 1.65)	

Reference (First author, publication year, S/N) Meta-analysis	Study design	Population: Study region Sample population Sample size (M, F, M+F): Age (mean, range) No. of cases / no. of controls or exposed/unexposed Time of recruitment / follow-up (mean, range) Response (%) (baseline minus loss to follow-up)	Outcome: Disease (ICD-10) Prescription Questionnaire	Exposure: exposure source	Exposure: exposure assessment	Exposure: exposure levels	Results	Comments (study quality [overall assessment according to SIGN/CASP], conflict of interest [stated vs. not stated], funding [financed from public funds vs. financed from industry], confounding, strengths / weaknesses [potential bias, over- or underestimation of potential effects])
							>60 to ≤65 1.52 (1.11, 2.07)<br>>65 1.19 (0.85, 1.68)<br><br>Using Model 1, for L_N, <55dB= Ref (Supplemental material)<br>Noise level RR<br>>50 to ≤55 1.41 (1.04, 1.91)<br>>55 to ≤60 1.14 (0.78, 1.65)<br>>60 1.30 (0.80, 2.09)<br><br>Sensitivity analysis show relative risks of high depressive symptoms at follow-up per education and insomnia at follow-up	
Schreck	Cross-	Study region:	Medications of:	Aircraft	$L_{eq,16h}$ (06-22h)	Categori	Results from Appendix A5:	study quality:

Reference (First author, publication year, S/N) Meta-analysis	Study design	Population: Study region Sample population Sample size (M, F, M+F): Age (mean, range) No. of cases / no. of controls or exposed/unexposed Time of recruitment / follow-up (mean, range) Response (%) (baseline minus loss to follow-up)	Outcome: Disease (ICD-10) Prescription Questionnaire	Exposure: exposure source	Exposure: exposure assessment	Exposure: exposure levels	Results	Comments (study quality [overall assessment according to SIGN/CASP], conflict of interest [stated vs. not stated], funding [financed from public funds vs. financed from industry], confounding, strengths / weaknesses [potential bias, over- or underestimation of potential effects])
enberg, D. 2009, H-2883, Germany (RDF-Studie (Regionaler Dialogforum Flughafen Frankfurt)) Yes	sectional study	Frankfurt International airport, Germany<br><br>Sample population: randomly selected residents:<br><br>Sample size: M+F=3795 participants in face-to-face interviews: M+F=2312 (including 1 person with unlikable address) statistical analysis: M+F= 2311 M= 1034, F= 1276, 1 sex change	- Mood stabilizers<br>- sedatives<br>in the past 12 month (Bundesgesundheitsurvey (BGS) 1998)<br>→ Scale: never, seldomy, 1-3x per month, less than one time a week, 1-2x a week, several times a week, daily		L_N (22-06h)<br><br>NAT_{55} (Count of aircraft over 55 dB) (06-22h) (22-06h)<br><br>L_{MAX55} (06-22h) (22-06h)<br><br>Exposure from 2003 in 66 residential	zed: 40-45dB 45-50dB 50-55dB 55-60dB 60-65dB<br><br>Reference categories: For $L_{eq,16h}$: 40-45dB L_N: <40dB	Tab. A-17:ORs (95 % CI) for $L_{eq,16h}$ in dB and mood stabilizers and sedatives (reference category: 40-45):<br>Mood stabilizers<br>45-50: OR: 1.9 (CI: 0.50-7.11)<br>50-55: OR: 1.5 (CI: 0.38-6.15)<br>55-60: OR:1.4 (CI: 0.38-5.28)<br>60-65:-<br>Sedatives:<br>45-50: OR: 1.0 (CI: 0.32-3.12)<br>50-55: OR: 0.9 (CI: 0.29-3.00)<br>55-60: OR: 0.9 (CI: 0.32-2.72)<br>60-65: OR: 0.5 (CI: 0.10-2.91)<br><br>Tab. A-18:ORs (95 % CI) for L_N in dB and mood stabilizers and sedatives	-<br>Cross-sectional study design<br><br>conflict of interest: none declared<br><br>funding: Regionales Dialogforum Flughafen Frankfurt<br><br>confounding (adjusted for): noise sensitivity, home ownership,

Reference (First author, publication year, S/N) Meta-analysis	Study design	Population: Study region Sample population Sample size (M, F, M+F): Age (mean, range) No. of cases / no. of controls or exposed/unexposed Time of recruitment / follow-up (mean, range) Response (%) (baseline minus loss to follow-up)	Outcome: Disease (ICD-10) Prescription Questionnaire	Exposure: exposure source	Exposure: exposure assessment	Exposure: exposure levels	Results	Comments (study quality [overall assessment according to SIGN/CASP], conflict of interest [stated vs. not stated], funding [financed from public funds vs. financed from industry], confounding, strengths / weaknesses [potential bias, over- or underestimation of potential effects])
		Age:<br>not reported: 28<br>< 18 years: 17<br>18-19 years: 52<br>20-29 years: 240<br>30-39 years: 293<br>40-49 years: 420<br>50-59 years: 344<br>60-69 years: 440<br>70-79 years: 322<br>≥ 80 years:155<br><br>Exposed/unexposed:<br>NA			areas (calculation from the addresses) within 40 km distance from the airport	$L_{eq,16h}$<br>- mean: 51.9 dB<br>- SD 6.2<br>L_N<br>- mean: 45.9 dB<br>- SD 6.6	(reference category: <40):<br>Mood stabilizers<br>40-45: OR: 0.64 (CI: 0.22-1.85)<br>45-50: OR: 0.44 (CI: 0.12-1.61)<br>50-55: OR: 0.82 (CI: 0.28-2.40)<br>55-60: OR: 0.22 (CI: 0.03-1.86)<br>Sedatives:<br>40-45: OR: 0.51 (CI: 0.19-1.42)<br>45-50: OR: 0.61 (CI: 0.20-1.81)<br>50-55: OR: 0.65 (CI: 0.23-1.83)<br>55-60: OR: 0.51 (CI: 0.13-2.05)<br><br>Above models adjusted for noise sensitivity, home ownership, residential satisfaction, windows closed at night, age, gender, time	residential satisfaction, windows closed at night, age, gender, time outside home, social status<br><br>strengths/ weaknesses:<br>+ multiple noise parameters<br>+ multiple regression analysis<br>+ exposure measurement<br>+ randomized selection of residents

Reference (First author, publication year, S/N) Meta-analysis	Study design	Population: Study region, Sample population, Sample size (M, F, M+F): Age (mean, range), No. of cases / no. of controls or exposed/unexposed, Time of recruitment / follow-up (mean, range), Response (%) (baseline minus loss to follow-up)	Outcome: Disease (ICD-10), Prescription, Questionnaire	Exposure: exposure source	Exposure: exposure assessment	Exposure: exposure levels	Results	Comments (study quality [overall assessment according to SIGN/CASP], conflict of interest [stated vs. not stated], funding [financed from public funds vs. financed from industry], confounding, strengths / weaknesses [potential bias, over- or underestimation of potential effects])
		Time of recruitment/ follow-up: time frame of the whole study 2004-2006 interviews from Apr.-Dec. 2005 noise calculations for 2005<br><br>Response: 61%					outside home, social status	(but method not reported)<br>- cross-sectional design<br>- self-reported measurement, but validated<br>- no information on age (mean, range)<br>- no other noise exposure considered<br>- air particulate level not considered
Seidler, 2017,	Case-control	Study region: Area Frankfurt International	Secondary data from three health	Aircraft Railway	Aircraft: $L_{eq, 24h}$	<40dB, max <50	Aircraft: $L_{eq, 24h}$<br>Noise OR Case Contro	study quality: + (to ++)

Reference (First author, publication year, S/N) Meta-analysis	Study design	Population<br>Study region<br>Sample population<br>Sample size (M, F, M+F):<br>Age (mean, range)<br>No. of cases / no. of controls or exposed/unexposed<br>Time of recruitment / follow-up (mean, range)<br>Response (%)<br>(baseline minus loss to follow-up)	Outcome<br>Disease (ICD-10)<br>Prescription<br>Questionnaire	Exposure<br>exposure source	 exposure assessment	 exposure levels	Results				Comments (study quality [overall assessment according to SIGN/CASP], conflict of interest [stated vs. not stated], funding [financed from public funds vs. financed from industry], confounding, strengths / weaknesses [potential bias, over- or underestimation of potential effects])
2322, Germany<br>+<br>Seidler A., Wagner M., Schubert M., Dröge P., Hegewald J., 2016a. Sekund	study	Airport<br><br>Sample population:<br>NORAH (all individuals living in the study area aged 40 years or older in 2010 an insured by one of three large statutory health insurance funds between 2005 and 2010)<br><br>Sample size:<br>N= 1,026,670<br>For analysis:<br>M+F= 655,541<br>M=317,153 F=338,388	insurance funds:<br><br>Disease<br>Depressive episodes (F32,-), recurrent depressive disorder (F33,-), dysthymia (F34.1), mixed anxiety and depression disorder (F41.2)<br><br>Newly diagnosed	Road traffic<br><br>measured in 2005 (partly 1996-2005)	L_N (22-06h) and other night segments<br><br>historical radar data from the German flight safety operator (DFS), analysis according to guidelines for calculations of noise abatement zones (AzB)	dB = reference category<br><br><40 dB, max ≥50 dB;<br>≥40<45dB<br>≥45<50dB<br>≥50<55dB<br>≥55<60dB<br>≥60<65dB<br>≥65<70dB<br><br>Start point for	level (dB)	(95% CI)	(n)	l (n)	conflict of interest: stated<br>funding: Gemeinnützige Umwelthaus GmbH<br><br>confounding (adjusted for): age, sex, urban living environment, local proportion of people receiving unemployment benefits as indicator of SES and if available
							<40, max <50	1.00	28,687	233,178	
							<40, max ≥50	1.01 (0.98-1.04)	4,647	37,668	
							≥40<45	1.13 (1.10-1.15)	24,081	170,171	
							≥45<50	1.18 (1.16-1.21)	13,231	90,227	
							≥50<55	1.23 (1.19-1.28	5,243	35,784	

Reference (First author, publication year, S/N) Meta-analysis	Study design	Population<br>Study region<br>Sample population<br>Sample size (M, F, M+F):<br>Age (mean, range)<br>No. of cases / no. of controls or exposed/unexposed<br>Time of recruitment / follow-up (mean, range)<br>Response (%)<br>(baseline minus loss to follow-up)	Outcome<br>Disease (ICD-10)<br>Prescription<br>Questionnaire	Exposure<br>exposure source	exposure assessment	exposure levels	Results	Comments (study quality [overall assessment according to SIGN/CASP], conflict of interest [stated vs. not stated], funding [financed from public funds vs. financed from industry], confounding, strengths / weaknesses [potential bias, over- or underestimation of potential effects])
ärdaten basierte Fallkontrollstudie mit vertiefender Befragung. NORAH (Noise-related annoyance, cognitio		Age:<br>≥ 40 years<br><br>No. of cases / no. of controls:<br>N_{cases}= 77,295<br>$N_{controls}$= 578,246<br><br>Time of recruitment / follow-up:<br>2006-2010<br><br>Response rate (%):<br>90.5% of cases and 90.7% of controls<br>(= data linkage diagnoses-noise)	cases 2006-2010		Railway and road traffic<br>$L_{eq,\ 24h}$<br>L_N (22-06h) and other night segments<br>Sound levels calculated by using estimates of traffic exposure and estimating sound reductions between source of sound and	continuous analysis: 35 dB (all values below 40dB set to 35dB)	)<br>≥55<60 | 1.09 (1.02-1.16) | 1,395 | 11,043<br>≥60<65 | 0.71 (0.38-1.31) | 11 | 155<br>≥65<70 | - | 0 | 0<br><br>Aircraft: L_N<br>Noise level (dB) | OR (95% CI) | Case (n) | Control (n)<br><40, | 1.00 | 33,82 | 268,29	education, job title<br><br>strengths/ weaknesses:<br>+ adequate definition of cases<br>+ adequate exposure assessment<br>+ consideration of maximum nightly aircraft levels (NAT6)<br>+ adequate definition of outcome disease (ICD classification)<br>+ high number of cases and controls

Reference (First author, publication year, S/N) Meta-analysis	Study design	Population: Study region Sample population Sample size (M, F, M+F): Age (mean, range) No. of cases / no. of controls or exposed/unexposed Time of recruitment / follow-up (mean, range) Response (%) (baseline minus loss to follow-up)	Outcome: Disease (ICD-10) Prescription Questionnaire	Exposure: exposure source	Exposure: exposure assessment	Exposure: exposure levels	Results				Comments (study quality [overall assessment according to SIGN/CASP], conflict of interest [stated vs. not stated], funding [financed from public funds vs. financed from industry], confounding, strengths / weaknesses [potential bias, over- or underestimation of potential effects])
n and health): Verkehrslärmwirkungen im Flughafenumfeld. Endbericht, Band 6, 2nd ed. Yes					and the immission sites according to the methods for calculation (VBUS, BUSCH) used for noise mapping + NAT-6		max <50		8	0	
							<40, max ≥50	1.07 (1.05-1.09)	20,990	152,047	- limited differentiation between incident and prevalent cases, therefore temporality cannot be totally established
							≥40<45	1.16 (1.13-1.18)	13,819	94,846	
							≥45<50	1.16 (1.13-1.20)	6,358	44,856	- lack of adjustment for air pollutants (particularly relevant for aircraft noise)
							≥50<55	1.06 (1.01-1.12)	2,234	17,352	
							≥55<60	0.72 (0.56-0.93	66	855	

Reference (First author, publication year, S/N) Meta-analysis	Study design	Population: Study region Sample population Sample size (M, F, M+F): Age (mean, range) No. of cases / no. of controls or exposed/unexposed Time of recruitment / follow-up (mean, range) Response (%) (baseline minus loss to follow-up)	Outcome: Disease (ICD-10) Prescription Questionnaire	Exposure: exposure source	Exposure: exposure assessment	Exposure: exposure levels	Results				Comments (study quality [overall assessment according to SIGN/CASP], conflict of interest [stated vs. not stated], funding [financed from public funds vs. financed from industry], confounding, strengths / weaknesses [potential bias, over- or underestimation of potential effects])
								)			
							≥60	-	0	0	
								Road traffic: L_{eq}			
							Noise level (dB)	OR (95% CI)	Case (n)	Control (n)	
							<40, max <50	1.00	7,728	62,733	
							≥40<45	1.02 (1.00-1.06)	15,885	124,699	
							≥45<50	1.06 (1.03-1.09	18,694	138,625	

		Population	Outcome	Exposure							
**Referen ce (First author, publica tion year, S/N) Meta-a nalysis**	**Study desig n**	**Study region<br>Sample population<br>Sample size (M, F, M+F):<br>Age (mean, range)<br>No. of cases / no. of controls or exposed/unexposed<br>Time of recruitment / follow-up (mean, range)<br>Response (%)<br>(baseline minus loss to follow-up)**	**Disease (ICD-10)<br>Prescription<br>Questionnaire**	**exposure source**	**exposure assessment**	**exposure levels**	**Results**				**Comments (study quality [overall assessment according to SIGN/CASP], conflict of interest [stated vs. not stated], funding [financed from public funds vs. financed from industry], confounding, strengths / weaknesses [potential bias, over- or underestimation of potential effects])**
								)			
							≥50<55	1.09 (1.06-1.12)	14,10 3	101,54 9	
							≥55<60	1.05 (1.01-1.08)	8,359	62,994	
							≥60<65	1.12 (1.08-1.16)	6,648	46,826	
							≥65<70	1.12 (1.08-1.17)	4,540	31,955	
							≥70	1.17 (1.10-1.25	1,338	8,865	

Reference (First author, publication year, S/N) Meta-analysis	Study design	Population: Study region Sample population Sample size (M, F, M+F): Age (mean, range) No. of cases / no. of controls or exposed/unexposed Time of recruitment / follow-up (mean, range) Response (%) (baseline minus loss to follow-up)	Outcome: Disease (ICD-10) Prescription Questionnaire	Exposure: exposure source	Exposure: exposure assessment	Exposure: exposure levels	Results	Comments (study quality [overall assessment according to SIGN/CASP], conflict of interest [stated vs. not stated], funding [financed from public funds vs. financed from industry], confounding, strengths / weaknesses [potential bias, over- or underestimation of potential effects])
							)<br><br>Continuous: 1.037 95% CI 1.028-1.046), p< 0.001<br><br>Road traffic: L_N<br>Noise level (dB) / OR (95% CI) / Case (n) / Control (n)<br><40, max <50 / 1.00 / 30,420 / 236,396<br>≥40<45 / 1.03 (1.01-1.05) / 15,822 / 117,229<br>≥45<50 / 1.04 / 12,40 / 90,574	

Reference (First author, publication year, S/N) Meta-analysis	Study design	Population: Study region<br>Sample population<br>Sample size (M, F, M+F):<br>Age (mean, range)<br>No. of cases / no. of controls or exposed/unexposed<br>Time of recruitment / follow-up (mean, range)<br>Response (%)<br>(baseline minus loss to follow-up)	Outcome: Disease (ICD-10)<br>Prescription<br>Questionnaire	Exposure: exposure source	Exposure: exposure assessment	Exposure: exposure levels	Results	Comments (study quality [overall assessment according to SIGN/CASP], conflict of interest [stated vs. not stated], funding [financed from public funds vs. financed from industry], confounding, strengths / weaknesses [potential bias, over- or underestimation of potential effects])
							(1.01-1.06) 7	
							≥50<55 1.03 (1.01-1.06) 8,912 65,645	
							≥55<60 1.07 (1.04-1.10) 6,445 45,856	
							≥60 1.11 (1.06-1.15) 3,289 22,546	
							Railway: L_{eq}	
							Noise level (dB) OR (95% CI) Case (n) Control (n)	

Reference (First author, publication year, S/N) Meta-analysis	Study design	Population: Study region Sample population Sample size (M, F, M+F): Age (mean, range) No. of cases / no. of controls or exposed/unexposed Time of recruitment / follow-up (mean, range) Response (%) (baseline minus loss to follow-up)	Outcome: Disease (ICD-10) Prescription Questionnaire	Exposure: exposure source	Exposure: exposure assessment	Exposure: exposure levels	Results				Comments (study quality [overall assessment according to SIGN/CASP], conflict of interest [stated vs. not stated], funding [financed from public funds vs. financed from industry], confounding, strengths / weaknesses [potential bias, over- or underestimation of potential effects])
							<40, max <50	1.00	40,213	314,545	
							≥40<45	1.03 (1.00-1.05)	9,652	71,811	
							≥45<50	1.11 (1.09-1.14)	12,929	89,372	
							≥50<55	1.13 (1.10-1.15)	8,925	61,695	
							≥55<60	1.06 (1.02-1.10)	3,362	24,862	

Reference (First author, publication year, S/N) Meta-analysis	Study design	Population: Study region Sample population Sample size (M, F, M+F): Age (mean, range) No. of cases / no. of controls or exposed/unexposed Time of recruitment / follow-up (mean, range) Response (%) (baseline minus loss to follow-up)	Outcome: Disease (ICD-10) Prescription Questionnaire	Exposure: exposure source	Exposure: exposure assessment	Exposure: exposure levels	Results				Comments (study quality [overall assessment according to SIGN/CASP], conflict of interest [stated vs. not stated], funding [financed from public funds vs. financed from industry], confounding, strengths / weaknesses [potential bias, over- or underestimation of potential effects])
							≥60<65	1.15 (1.08-1.22)	1,371	9,371	
							≥65<70	1.07 (0.98-1.17)	556	4,129	
							≥70	0.93 (0.82-1.06)	287	2,461	
							Railway: L_N				
							Noise level (dB)	OR (95% CI)	Case (n)	Control (n)	
							<40, max	1.00	39,834	312,270	

		Population	Outcome	Exposure							
**Reference (First author, publication year, S/N) Meta-analysis**	**Study design**	**Study region Sample population Sample size (M, F, M+F): Age (mean, range) No. of cases / no. of controls or exposed/unexposed Time of recruitment / follow-up (mean, range) Response (%) (baseline minus loss to follow-up)**	**Disease (ICD-10) Prescription Questionnaire**	**exposure source**	**exposure assessment**	**exposure levels**	**Results**				**Comments (study quality [overall assessment according to SIGN/CASP], conflict of interest [stated vs. not stated], funding [financed from public funds vs. financed from industry], confounding, strengths / weaknesses [potential bias, over- or underestimation of potential effects])**
							<50				
							≥40<45	1.03 (1.00-1.05)	9,565	70,989	
							≥45<50	1.12 (1.09-1.14)	12,582	85,965	
							≥50<55	1.12 (1.09-1.15)	9,101	63,277	
							≥55<60	1.06 (1.02-1.10)	3,840	28,287	
							≥60	1.07 (1.02-1.17)	2,373	17,458	

Reference (First author, publication year, S/N) Meta-analysis	Study design	Population: Study region Sample population Sample size (M, F, M+F): Age (mean, range) No. of cases / no. of controls or exposed/unexposed Time of recruitment / follow-up (mean, range) Response (%) (baseline minus loss to follow-up)	Outcome: Disease (ICD-10) Prescription Questionnaire	Exposure: exposure source	Exposure: exposure assessment	Exposure: exposure levels	Results	Comments (study quality [overall assessment according to SIGN/CASP], conflict of interest [stated vs. not stated], funding [financed from public funds vs. financed from industry], confounding, strengths / weaknesses [potential bias, over- or underestimation of potential effects])
							Subanalysis: Including only subjects with known socio-economic state: *Aircraft*: reversed U-shape disappeared for 24 h sound levels, but remained – albeit less distinct – in the analysis of the night time periods. Higher ORs (50 to < 55 dB noise category: 1.35 (95% CI= 1.28-1.43) and above 60 dB: 1.37 (95% CI 0.65–2.91)). *Road traffic:* Higher ORs (65 and < 70 dB:1.25 (95% CI=1.17–1.33) and 5.2% increase per 10 dB for continuous noise) *Railway:* comparable to main analysis	

Reference (First author, publication year, S/N) Meta-analysis	Study design	Population: Study region Sample population Sample size (M, F, M+F): Age (mean, range) No. of cases / no. of controls or exposed/unexposed Time of recruitment / follow-up (mean, range) Response (%) (baseline minus loss to follow-up)	Outcome: Disease (ICD-10) Prescription Questionnaire	Exposure: exposure source	Exposure: exposure assessment	Exposure: exposure levels	Results	Comments (study quality [overall assessment according to SIGN/CASP], conflict of interest [stated vs. not stated], funding [financed from public funds vs. financed from industry], confounding, strengths / weaknesses [potential bias, over- or underestimation of potential effects])
							Combined exposure lower ORs and similar exposure-risk relations. + a reference group without any type of traffic noise exposure of 40 dB or more (and excluding maximum nightly aircraft noise of 50 dB or more): Risk estimates higher than for any of the separate traffic noise exposure categories: Combined exposure to all three types of traffic noise resulted in an OR of 1.42 (95% CI=1.33–1.52).	
Stansfeld, S.	Longitudinal	Study region: Caerphilly	Psychiatric caseness	Road traffic	$L_{eq,16h}$ (06-22h)	51-55 dB, 56-60 dB,	Association between road traffic noise level and psychiatric disorder	study quality: + (for cross-sectional

Reference (First author, publication year, S/N) Meta-analysis	Study design	Population: Study region Sample population Sample size (M, F, M+F): Age (mean, range) No. of cases / no. of controls or exposed/unexposed Time of recruitment / follow-up (mean, range) Response (%) (baseline minus loss to follow-up)	Outcome: Disease (ICD-10) Prescription Questionnaire	Exposure: exposure source	Exposure: exposure assessment	Exposure: exposure levels	Results	Comments (study quality [overall assessment according to SIGN/CASP], conflict of interest [stated vs. not stated], funding [financed from public funds vs. financed from industry], confounding, strengths / weaknesses [potential bias, over- or underestimation of potential effects])
1996, 2441, UK<br>Yes sum score/ No subscales (only means given)<br><br>Evaluated togethe	study	Sample population:<br>From Caerphilly collaborative heart study, men aged 50-64 years living in Caerphilly and its environs<br><br>Sample size:<br>(only male)<br>N=2,398<br>5-year follow-up:<br>N=1,725<br><br>Age:<br>50-64 years<br><br>No of cases/ no of controls: /	GHQ 30<br>- Sum score<br>Psychiatric caseness:<br>threshold 4/5<br>-Anxiety subscale<br>-Depression subscale<br>(validated against psychiatric interview in a subsample)	noise		61-65 dB, 66-70 dB<br><br>No noise value under 51 dB	(from Table 1)<br><br>GHQ adjusted for age, social class, employment status, marital status, physical ill health, and baseline GHQ score (n = 1,590). Values are mean (SE) score on GHQ<br>51-55 dB: 2.57 (0.21), n = 1,218<br>56-60 dB: 3.37 (0.39), n = 153<br>61-65 dB: 2.65 (0.34), n = 233<br>66-70 dB: 2.96 (0.46), n = 104<br>(p-value for test of heterogeneity: 0.29)<br><br>Psychiatric caseness (% scoring ≥ 5 on GHQ):	analysis: -)<br><br>conflict of interest: none<br><br>funding:<br>Medical Research Council<br><br>confounding (controlled for):<br>age, social class, employment status, noise sensitivity (anxiety/depression at baseline) and marital

Reference (First author, publication year, S/N) Meta-analysis	Study design	Population: Study region Sample population Sample size (M, F, M+F): Age (mean, range) No. of cases / no. of controls or exposed/unexposed Time of recruitment / follow-up (mean, range) Response (%) (baseline minus loss to follow-up)	Outcome: Disease (ICD-10) Prescription Questionnaire	Exposure: exposure source	Exposure: exposure assessment	Exposure: exposure levels	Results	Comments (study quality [overall assessment according to SIGN/CASP], conflict of interest [stated vs. not stated], funding [financed from public funds vs. financed from industry], confounding, strengths / weaknesses [potential bias, over- or underestimation of potential effects])
r with Stansfeld 1993 (2453)		Time of recruitment/ follow-up:<br>Time of recruitment missing*<br>follow-up 5 years later<br><br>Response:<br>response at baseline= 89%<br>no information about baseline population (also not in original paper: Caerphilly and Speedwell collaborative heart disease studies. 1984)<br><br>Loss to follow-up= 23%					51-55 dB: 22.5, n = 1,218<br>56-60 dB: 32.0, n = 153<br>61-65 dB: 24.9, n = 233<br>66-70 dB: 25.0, n = 104<br>(p-value for test of heterogeneity: 0.29)<br><br>Mean (SE) anxiety score adjusted for age, social class, and noise sensitivity and anxiety at baseline (n = 1583)<br>51-55 dB: 4.70 (0.07), n = 1,218<br>56-60 dB: 5.20 (0.18), n = 153<br>61-65 dB: 4.89 (0.15), n = 233<br>66-70 dB: 5.02 (0.21), n = 104<br>(p-value for test of heterogeneity:	status and physical ill health for GHQ<br><br>strengths, weaknesses:<br>+longitudinal study (follow-up 5 years)<br>+good response, low loss to follow up<br>+ power calculation<br>+non-responder analysis<br>- age restriction 50-64, (Caerphilly sample 30-69 years): anxiety disorders "start in

Reference (First author, publication year, S/N) Meta-analysis	Study design	Population: Study region Sample population Sample size (M, F, M+F): Age (mean, range) No. of cases / no. of controls or exposed/unexposed Time of recruitment / follow-up (mean, range) Response (%) (baseline minus loss to follow-up)	Outcome: Disease (ICD-10) Prescription Questionnaire	Exposure: exposure source	exposure assessment	exposure levels	Results	Comments (study quality [overall assessment according to SIGN/CASP], conflict of interest [stated vs. not stated], funding [financed from public funds vs. financed from industry], confounding, strengths / weaknesses [potential bias, over- or underestimation of potential effects])
							0.03)<br><br>Mean (SE) depression score, adjusted for age, social class, and noise sensitivity and depression at baseline (n = 1587)<br>51-55 dB: 1.19 (0.05), n = 1,218<br>56-60 dB: 1.39 (0.13), n = 153<br>61-65 dB: 1.32 (0.11), n = 233<br>66-70 dB: 1.21 (0.16), n = 104<br>(p-value for test of heterogeneity: 0.34)	childhood, adolescence, or early adulthood until they reach a peak in middle age, then tending to decrease again with older age"<br>- generalizability (only men and only Leq)<br>- no other noise exposure considered<br>- air particulate level not considered

Reference (First author, publication year, S/N) Meta-analysis	Study design	Population: Study region, Sample population, Sample size (M, F, M+F): Age (mean, range), No. of cases / no. of controls or exposed/unexposed, Time of recruitment / follow-up (mean, range), Response (%) (baseline minus loss to follow-up)	Outcome: Disease (ICD-10), Prescription, Questionnaire	Exposure: exposure source	Exposure: exposure assessment	Exposure: exposure levels	Results	Comments (study quality [overall assessment according to SIGN/CASP], conflict of interest [stated vs. not stated], funding [financed from public funds vs. financed from industry], confounding, strengths / weaknesses [potential bias, over- or underestimation of potential effects])
Stansfeld, S., 1993, 2453, UK	Cross-sectional study	Study region: Caerphilly<br><br>Study population: Caerphilly Collaborative Heart Disease Study<br><br>Sample size: only males N = 2,398<br><br>Age: 50-64 years<br><br>Exposed/Unexposed: see results	Psychiatric caseness GHQ-30 (threshold ≥ 5)	Road traffic	$L_{eq,16h}$ (06-22h)	51-55 dB, 56-60 dB, 61-65 dB, 66-70 dB	Traffic noise exposure level and percentage of psychiatric caseness (from Table 4)<br><br>Noise (dB) / Not a case (GHQ 0-4) / Prob. Case (GHQ 5+) / Total (n)<br>51-55 / 78.7 (1,233) / 21.3 (333) / 1,566<br>56-60 / 73.5 (144) / 26.5 (52) / 196<br>61-65 / 76.8 (208) / 23.3 (63) / 271<br>66-70 / 75.2 (103) / 24.8 (34) / 137	

Reference (First author, publication year, S/N) Meta-analysis	Study design	Population: Study region Sample population Sample size (M, F, M+F): Age (mean, range) No. of cases / no. of controls or exposed/unexposed Time of recruitment / follow-up (mean, range) Response (%) (baseline minus loss to follow-up)	Outcome: Disease (ICD-10) Prescription Questionnaire	Exposure: exposure source	Exposure: exposure assessment	Exposure: exposure levels	Results	Comments (study quality [overall assessment according to SIGN/CASP], conflict of interest [stated vs. not stated], funding [financed from public funds vs. financed from industry], confounding, strengths / weaknesses [potential bias, over- or underestimation of potential effects])
		Time of recruitment/ follow-up: "Established in the early 1980's" first follow-up 5 years later<br><br>Response rate: no information<br><br>(Longitudinal study, but baseline was not analyzed) + "At first follow-up the cohort was reconstructed with men new to the area)					Total 1,688 482 2,170<br>Mantel Haenszel x2 trend = 1.87 (p=0.17)<br><br>Stratification by sensitivity: Traffic noise and percentage of psychiatric caseness stratified by tertiles of Weinstein Noise Sensitivity (from Table 6)<br><br>Noise sensitivity | 51-55 | 56-60 | 61-65 | 66-70 | Total	

Reference (First author, publication year, S/N) Meta-analysis	Study design	Population: Study region Sample population Sample size (M, F, M+F): Age (mean, range) No. of cases / no. of controls or exposed/unexposed Time of recruitment / follow-up (mean, range) Response (%) (baseline minus loss to follow-up)	Outcome: Disease (ICD-10) Prescription Questionnaire	Exposure: exposure source	Exposure: exposure assessment	Exposure: exposure levels	Results						Comments (study quality [overall assessment according to SIGN/CASP], conflict of interest [stated vs. not stated], funding [financed from public funds vs. financed from industry], confounding, strengths / weaknesses [potential bias, over- or underestimation of potential effects])
							1st tertile (low sens.)	10.2 (53/522)	15.8 (12/76)	18.9 (20/106)	18.4 (9/49)	12.5 (94/753)*	
							2nd tertile	19.8 (103/521)	20.9 (14/67)	22.6 (19/84)	29.6 (13/44)	20.8 (149/716)	
							3rd tertile (high	33.9 (171/505)	49.0 (14/49)	28.8 (23/80)	27.9 (12/43)	34.0 (230/677)	

Reference (First author, publication year, S/N) Meta-analysis	Study design	Population: Study region; Sample population; Sample size (M, F, M+F): Age (mean, range); No. of cases / no. of controls or exposed/unexposed; Time of recruitment / follow-up (mean, range); Response (%) (baseline minus loss to follow-up)	Outcome: Disease (ICD-10) Prescription Questionnaire	Exposure: exposure source	Exposure: exposure assessment	Exposure: exposure levels	Results	Comments (study quality [overall assessment according to SIGN/CASP], conflict of interest [stated vs. not stated], funding [financed from public funds vs. financed from industry], confounding, strengths / weaknesses [potential bias, over- or underestimation of potential effects])
							sens .)<br><br>*p< 0.05	
Stansfeld, S. 2009, 2442, UK No (noise measurements in 3 hours	Longitudinal before and after study	Study region: Three towns in Clwyd, North Wales<br><br>Sample population: Adults over 16 years living in households, originally recruited for the Respiratory Health Study<br><br>Sample size:	Common Mental Disorder CIS-R (Revised Clinical Interview Schedule)<br><br>Mental Health -GHQ 28 (total)	Road traffic	-$L_{eq,10\text{-}17h}$<br>-L_{10} (noise level exceeded for 10% of time of measurement duration)<br><br>(over 3 hours and 15 minutes)<br><br>Baseline	Congested (high noise area)/ non-congested streets (low noise area)	Prevalence of common mental disorder (CIS-R):<br>Baseline:<br>control area: 22.9% (95% CI 12-41)<br>exposed area: 14.9% (95%CI 8-28)<br><br>After bypass opened:<br>control area: 13.6% (95% CI 6-30)<br>exposed area: 10.0% (95% CI 4-26)	study quality: -(to --)<br><br>conflict of interest: stated<br><br>funding: Nil<br><br>confounding (controlled for): no adjustment

		Population	Outcome	Exposure				
**Reference (First author, publication year, S/N) Meta-analysis**	**Study design**	**Study region<br>Sample population<br>Sample size (M, F, M+F):<br>Age (mean, range)<br>No. of cases / no. of controls or exposed/unexposed<br>Time of recruitment / follow-up (mean, range)<br>Response (%)<br>(baseline minus loss to follow-up)**	**Disease (ICD-10)<br>Prescription<br>Questionnaire**	**exposure source**	**exposure assessment**	**exposure levels**	**Results**	**Comments<br>(study quality [overall assessment according to SIGN/CASP], conflict of interest [stated vs. not stated], funding [financed from public funds vs. financed from industry], confounding, strengths / weaknesses [potential bias, over- or underestimation of potential effects])**
15 minutes (not standard)		At baseline:<br>M+F= 337<br>At follow-up:<br>M+F= 228<br><br>Targeted household sample= 387 households<br><br>Age:<br>16 -90 years<br><br>Exposed/unexposed:<br>exposed (facing main streets):<br>at baseline, M+F= 98<br>at follow-up, M+F= 67			December 1997;<br>Follow-up<br>December 1998		Mean health outcome scores at baseline unadjusted and adjusted for deprivation in the high noise and control samples (from Table 4)<br>Outcome / High noise (n=98) / Control sample (n=239) / p<br>GHQ adjusted for deprivation / 3.2 / 3.8 / 0.38	strengths, weaknesses:<br>- no statistical analysis<br>- no adjustment for confounders<br>- no information on participant characteristics<br>-.9% versus 14.9%)<br>- not clear as to which areas are exposed an unexposed<br>- no other noise exposure considered<br>- air particulate level

Reference (First author, publication year, S/N) Meta-analysis	Study design	Population<br>Study region<br>Sample population<br>Sample size (M, F, M+F):<br>Age (mean, range)<br>No. of cases / no. of controls or exposed/unexposed<br>Time of recruitment / follow-up (mean, range)<br>Response (%)<br>(baseline minus loss to follow-up)	Outcome<br>Disease (ICD-10)<br>Prescription<br>Questionnaire	Exposure<br>exposure source	exposure assessment	exposure levels	Results	Comments (study quality [overall assessment according to SIGN/CASP], conflict of interest [stated vs. not stated], funding [financed from public funds vs. financed from industry], confounding, strengths / weaknesses [potential bias, over- or underestimation of potential effects])
		unexposed (uncongested streets):<br>at baseline, M+F=239<br>at follow-up, M+F=161<br><br>Time of recruitment/ follow-up:<br>October/November 1997/ October –November 1998<br>(Bypass opened in March 1998)<br><br>Response:<br>70% at baseline (63% in high noise, 76% in low noise).<br>Loss to follow-up:						not considered

Reference (First author, publication year, S/N) Meta-analysis	Study design	Population: Study region Sample population Sample size (M, F, M+F): Age (mean, range) No. of cases / no. of controls or exposed/unexposed Time of recruitment / follow-up (mean, range) Response (%) (baseline minus loss to follow-up)	Outcome: Disease (ICD-10) Prescription Questionnaire	Exposure: exposure source	Exposure: exposure assessment	Exposure: exposure levels	Results	Comments (study quality [overall assessment according to SIGN/CASP], conflict of interest [stated vs. not stated], funding [financed from public funds vs. financed from industry], confounding, strengths / weaknesses [potential bias, over- or underestimation of potential effects])
		26% (low noise: 22%, high noise 31%)<br><br>(Sample originally recruited from the Respiratory Health Study: Burr ML, Karani G, Davies B, Holmes BA, Williams KL. Effects on respiratory health of a reduction in air pollution from vehicle exhaust emissions. Occup Environ Med 2004; 61:212-8.)						
Stansfeld, 2009, 2447.	Cross-sectional	Study region: Area around Heathrow airport (London), Schiphol	Psychological morbidity: Strength and	Aircraft and road traffic	Noise exposure assessments at schools	30-77dB for aircraft	Exposure to aircraft noise traffic in dB and mental health outcomes (Table 2) [illegible] p-val	Study quality -(to +)

Reference (First author, publication year, S/N) Meta-analysis	Study design	Population: Study region<br>Sample population<br>Sample size (M, F, M+F):<br>Age (mean, range)<br>No. of cases / no. of controls or exposed/unexposed<br>Time of recruitment / follow-up (mean, range)<br>Response (%)<br>(baseline minus loss to follow-up)	Outcome: Disease (ICD-10)<br>Prescription<br>Questionnaire	Exposure: exposure source	Exposure: exposure assessment	Exposure: exposure levels	Results			Comments (study quality [overall assessment according to SIGN/CASP], conflict of interest [stated vs. not stated], funding [financed from public funds vs. financed from industry], confounding, strengths / weaknesses [potential bias, over- or underestimation of potential effects])
No (continuous outcome)	study	Airport (Amsterdam), and Barajas airport (Madrid)<br><br>Study population:<br>RANCH Study<br>Pupils from 89 schools<br>Schools excluded if highly sound insulated or exposed to a different dominant noise source than to aircraft or road traffic noise.<br>Schools classified on a 4x4 grid ranging from low to high for aircraft noise and low to high for road traffic noise.<br>Two schools/country in each	Difficulties Questionnaire (SDQ)<br><br>Hyperactivity/ inattention<br>Conduct Problems<br>Emotional symptoms<br>Peer problems<br>Prosocial behavior<br>Total score (hyperactivity, emotional,	noise	Aircraft noise: $L_{eq,16h}$ (07-23h) noise contours<br><br>Road traffic noise:<br><br>In UK and Spain estimates of road traffic noise based on simplified CRTN noise prediction	noise<br><br>32-71dB for road traffic noise<br><br>Continuous analysis	Model 2<br>Model 2<br>Model 2<br>Model 2<br>Model 2	(95%CI)<br>Overall difficulties<br>0.013<br>(-0.023, 0.010)<br>Hyperactivity<br>0.013<br>(0.001, 0.024)<br>Conduct disorder<br>-0.005<br>(-0.013, 0.003)<br>Peer Problems<br>0.004<br>(-0.004, 0.012)<br>Prosocial behavior<br>0.002<br>(-0.007, 0.010)	0.471<br>0.032<br>0.220<br>0.296<br>0.720	Conflict of interest<br>Sponsors had no role in study design, data collection, analysis, interpretation or writing the report<br><br>Funding<br>Public<br>RANCH Study founded by European Community<br>UK co-founding by Department of Environment, Food and Rural Affairs.

Reference (First author, publication year, S/N) Meta-analysis	Study design	Population<br>Study region<br>Sample population<br>Sample size (M, F, M+F):<br>Age (mean, range)<br>No. of cases / no. of controls or exposed/unexposed<br>Time of recruitment / follow-up (mean, range)<br>Response (%)<br>(baseline minus loss to follow-up)	Outcome<br>Disease (ICD-10)<br>Prescription<br>Questionnaire	Exposure<br>exposure source	exposure assessment	exposure levels	Results	Comments<br>(study quality [overall assessment according to SIGN/CASP], conflict of interest [stated vs. not stated], funding [financed from public funds vs. financed from industry], confounding, strengths / weaknesses [potential bias, over- or underestimation of potential effects])
		noise exposure grid cell<br><br>Sample size:<br>M+F= 2014<br>Not separated by gender<br><br>Age:<br>9-10 years<br><br>Exposed/unexposed:<br>NA<br><br>Time of recruitment/follow-up:<br>Not specified	conduct and peer problems)<br><br>Parental version		method using a combination of proximity to roads. Measurements confirmed these estimates. For Netherlands aircraft and outdoor road traffic noise measurements provided by modelled data lined to school locations by		Emotional Problems<br>Model 2 0.001 (-0.009, 0.011) 0.785<br>Model 2: adjusted for age, gender, country, mother's education, employment status, crowding, homeownership, long-standing illness, main language spoken at home, parental support, classroom glazing and other noise exposure<br><br>Exposure to road noise traffic in dB and mental health outcomes<br>[illegible] (95%CI) p-val<br>Overall difficulties	Netherlands co-founding by the Dutch Ministry of Spatial Planning, Housing and Environment and the Dutch Ministry of Transport, Public Works and Water Management<br><br>Confounding<br>Age, gender, country, mothers education, employment status,

Reference (First author, publication year, S/N) Meta-analysis	Study design	Population<br>Study region<br>Sample population<br>Sample size (M, F, M+F):<br>Age (mean, range)<br>No. of cases / no. of controls or exposed/unexposed<br>Time of recruitment / follow-up (mean, range)<br>Response (%)<br>(baseline minus loss to follow-up)	Outcome<br>Disease (ICD-10)<br>Prescription<br>Questionnaire	Exposure<br>exposure source	exposure assessment	exposure levels	Results	Comments (study quality [overall assessment according to SIGN/CASP], conflict of interest [stated vs. not stated], funding [financed from public funds vs. financed from industry], confounding, strengths / weaknesses [potential bias, over- or underestimation of potential effects])
		Response rate (%)<br>Overall child response rate: 80%<br>Parental response rate: 80%			geographical information systems.		Model 2 -0.018 (-0.049, 0.013) 0.275<br>Hyperactivity<br>Model 2 0.0002 (-0.014, 0.014) 0.982<br>Conduct disorder<br>Model 2 -0.010 (-0.020, -0.001) 0.033<br>Peer Problems<br>Model 2 -0.009 (-0.019, 0.001) 0.072<br>Prosocial behavior<br>Model 2 -0.004 (-0.014, 0.007) 0.490<br>Emotional Problems<br>Model 0.001 0.828	crowding, homeownership, illness, main language spoken at home, parental support, classroom glazing<br><br>Strengths/weaknesses:<br>+ Studied both aircraft and road traffic noise<br>+ Good noise exposure assessment<br>+ Confounder list pretty inclusive, but missing air particulate

Reference (First author, publication year, S/N) Meta-analysis	Study design	Population: Study region Sample population Sample size (M, F, M+F): Age (mean, range) No. of cases / no. of controls or exposed/unexposed Time of recruitment / follow-up (mean, range) Response (%) (baseline minus loss to follow-up)	Outcome: Disease (ICD-10) Prescription Questionnaire	Exposure: exposure source	Exposure: exposure assessment	Exposure: exposure levels	Results	Comments (study quality [overall assessment according to SIGN/CASP], conflict of interest [stated vs. not stated], funding [financed from public funds vs. financed from industry], confounding, strengths / weaknesses [potential bias, over- or underestimation of potential effects])
							2 (-0.011, 0.014)<br><br>Model 2: adjusted for age, gender, country, mother's education, employment status, crowding, homeownership, long-standing illness, main language spoken at home, parental support, classroom glazing and other noise exposure	level<br>+ Good response rate<br>+ all schools matched according to socio-economic status and ethnicity within each country<br>+ study introduced as study on environment and health without explicit mention of noise<br>+ multi-country<br>- Only noise exposure at school considered<br>- cross-sectional study

Reference (First author, publication year, S/N) Meta-analysis	Study design	Population: Study region Sample population Sample size (M, F, M+F): Age (mean, range) No. of cases / no. of controls or exposed/unexposed Time of recruitment / follow-up (mean, range) Response (%) (baseline minus loss to follow-up)	Outcome: Disease (ICD-10) Prescription Questionnaire	Exposure: exposure source	exposure assessment	exposure levels	Results	Comments (study quality [overall assessment according to SIGN/CASP], conflict of interest [stated vs. not stated], funding [financed from public funds vs. financed from industry], confounding, strengths / weaknesses [potential bias, over- or underestimation of potential effects])
								- air particulate level not considered<br>- no information on the sampling method (selection)
Sygna, 2014, 2498 Yes	Cross-sectional study	Study region:<br>Oslo, Norway<br><br>Sample population:<br>Data on residential addresses obtained by Norwegian Public Roads Administration and City of Oslo. Using the Norwegian National	Psychological distress measured by Hopkins Symptoms Checklist-25 (HSCL-25)<br><br>Mean	Road traffic noise	L_{DEN}	Continuous variable<br><br>No start point specified (Figure shows	Association between road traffic noise exposure and Hopkins Symptom Checklist ≥1. (Per 10dB increase in noise, fromTable 5)<br>All: 1.05 (0.92, 1.21) (Adjusted model, OR (95%CI))	Study quality<br>-<br>Cross-sectional<br><br>Conflict of interest<br>No conflict of interest declared.<br><br>Funding

Reference (First author, publication year, S/N) Meta-analysis	Study design	Population<br>Study region<br>Sample population<br>Sample size (M, F, M+F):<br>Age (mean, range)<br>No. of cases / no. of controls or exposed/unexposed<br>Time of recruitment / follow-up (mean, range)<br>Response (%)<br>(baseline minus loss to follow-up)	Outcome<br>Disease (ICD-10)<br>Prescription<br>Questionnaire	Exposure<br>exposure source	exposure assessment	exposure levels	Results	Comments (study quality [overall assessment according to SIGN/CASP], conflict of interest [stated vs. not stated], funding [financed from public funds vs. financed from industry], confounding, strengths / weaknesses [potential bias, over- or underestimation of potential effects])
		Population Register, sampled from residential addresses, 51% males and 49% females from different age strata<br><br>Sample size:<br>M+F=2898<br>M= 1442<br>F= 1456<br><br>Age:<br>≤25 years: 210<br>26-35 years: 469<br>36-50 years: 924<br>51-65 years: 822<br>≥66 years: 472	psychological distress score used as a continuous variable and used as dichotomized variable for more severe levels of psychosocial stress (probable mental disorder cutoff ≥1.55 on HSCL)			modelled L_{DEN} values < 30dB)		Public<br>Norwegian Research Council and the Norwegian Public Roads Administration<br><br>Confounding<br>Adjusted for sex, age education, employment, noise sensitivity, and somatic diseases<br><br>Strengths/weaknesses:<br>+ good exposure

Reference (First author, publication year, S/N) Meta-analysis	Study design	Population: Study region Sample population Sample size (M, F, M+F): Age (mean, range) No. of cases / no. of controls or exposed/unexposed Time of recruitment / follow-up (mean, range) Response (%) (baseline minus loss to follow-up)	Outcome: Disease (ICD-10) Prescription Questionnaire	Exposure: exposure source	Exposure: exposure assessment	Exposure: exposure levels	Results	Comments (study quality [overall assessment according to SIGN/CASP], conflict of interest [stated vs. not stated], funding [financed from public funds vs. financed from industry], confounding, strengths / weaknesses [potential bias, over- or underestimation of potential effects])
		No. of cases<br>HSCL score<br>(probable mental disorder cutoff)<br><1.55: 2,124<br>≥1.55: 528<br><br>Time of recruitment/follow-up:<br>Autumn 2000<br><br>Response (%)<br>60.5%						assessment<br>+ appropriate outcome assessment<br>+ large sample size<br>+ consideration of important confounders<br>- air particulate level not considered<br>- No other noise exposure considered<br>- cross-sectional design<br>- large number of missing values, most linked to

**Reference (First author, publication year, S/N) Meta-analysis**	**Study design**	**Population**<br>**Study region**<br>**Sample population**<br>**Sample size (M, F, M+F):**<br>**Age (mean, range)**<br>**No. of cases / no. of controls or exposed/unexposed**<br>**Time of recruitment / follow-up (mean, range)**<br>**Response (%)**<br>**(baseline minus loss to follow-up)**	**Outcome**<br>**Disease (ICD-10)**<br>**Prescription**<br>**Questionnaire**	**Exposure**<br>**exposure source**	**exposure assessment**	**exposure levels**	**Results**	**Comments**<br>**(study quality [overall assessment according to SIGN/CASP], conflict of interest [stated vs. not stated], funding [financed from public funds vs. financed from industry], confounding, strengths / weaknesses [potential bias, over- or underestimation of potential effects])**
								psychological distress → participants who had not answered the HSCL items satisfactorily were excluded
Tzivian, M. 2016, 2586, Germany<br>-	Cross-sectional analysis of cohort study	Study region:<br>Ruhr area (Bochum, Essen, and Mülheim/Ruhr)<br><br>Sample population:<br>Heinz Nixdorf Recall Study (population-based cohort in the German Ruhr area)	Mild cognitive impairment (MCI)<br><br>(Diagnoses according to Petersen, 2004 "Mild cognitive	Road traffic noise	L_{DEN}<br>L_{N} (22-06 h)<br><br>Traffic load at major roads (vehicles × m/day) per 100,000	Dichotomized with cutpoint at 60 dB (L_{DEN}) and 55 dB (L_{N})	Associations of noise with MCI, OR (95% CI) per 10dB, from Tables 3 and S2<br><br>| | Overall MCI | Amnestic MCI | Non-amnestic MCI | | L_{DEN} | 1.40 | 1.53 | 1.26 |	study quality:<br>+ (to -)<br><br>conflict of interest:<br>none<br><br>funding:<br>German Ministry of

Reference (First author, publication year, S/N) Meta-analysis	Study design	Population<br>Study region<br>Sample population<br>Sample size (M, F, M+F):<br>Age (mean, range)<br>No. of cases / no. of controls or exposed/unexposed<br>Time of recruitment / follow-up (mean, range)<br>Response (%)<br>(baseline minus loss to follow-up)	Outcome<br>Disease (ICD-10)<br>Prescription<br>Questionnaire	Exposure<br>exposure source	exposure assessment	exposure levels	Results				Comments (study quality [overall assessment according to SIGN/CASP], conflict of interest [stated vs. not stated], funding [financed from public funds vs. financed from industry], confounding, strengths / weaknesses [potential bias, over- or underestimation of potential effects])
		Sample size:<br>At follow-up:<br>M+F=4157<br>Cognitive status available:<br>M+F= 4086<br>Statistical analysis:<br>M+F= 2050<br><br>Age:<br>50-80 years<br><br>Exposed/unexposed<br>NA<br><br>Time of recruitment/	impairment as a diagnostic entity". J Intern Med 256:183–194)<br><br>People with dementia excluded		Modelled in2007	Sensitivity analysis:<br><br>a) dichotomized at 65 dB (L_{DEN}) and 50 dB (L_N)<br><br>b) conti-nuous	(from 60 dB)<br>L_{DEN} (from 65 dB)*<br>L_N (from 55 dB)<br>L_N (from 50 dB)*	(1.03, 1.91)<br>1.89 (1.10, 3.25)<br>1.80 (1.07, 3.04)<br>1.35 (1.00, 1.82)	(1.05, 2.24)<br>2.39 (1.28, 4.45)<br>2.25 (1.23, 4.12)<br>1.47 (1.02, 2.12)	(0.82, 1.93)<br>1.34 (0.60, 2.98)<br>1.31 (0.60, 2.85)<br>1.22 (0.85, 1.85)	Education and Science and by the German Research Council<br><br>confounding (controlled for):<br>Main model: age, sex, SES (education), alcohol consumption, smoking status, self-reported passive exposure to tobacco smoke, regular physical activity,
							*From sensitivity analysis				

Reference (First author, publication year, S/N) Meta-analysis	Study design	Population: Study region Sample population Sample size (M, F, M+F): Age (mean, range) No. of cases / no. of controls or exposed/unexposed Time of recruitment / follow-up (mean, range) Response (%) (baseline minus loss to follow-up)	Outcome: Disease (ICD-10) Prescription Questionnaire	Exposure: exposure source	Exposure: exposure assessment	Exposure: exposure levels	Results	Comments (study quality [overall assessment according to SIGN/CASP], conflict of interest [stated vs. not stated], funding [financed from public funds vs. financed from industry], confounding, strengths / weaknesses [potential bias, over- or underestimation of potential effects])
		follow-up: 2006-2008<br><br>Baseline response: 53% (Stang et al. 2005)<br><br>Loss-to-follow up: about 15%				(startpoint apparently 0 dB)<br><br>c) In 10 dB categories<br>≥ 45 - < 55 dB;<br>≥ 55 - < 65 dB;<br>≥ 65 - < 75 dB;<br>≥ 75 dB	Adjusted for age, sex, SES, alcohol consumption, smoking, self-reported environmental tobacco smoke, regular physical activity, BMI<br><br>Effect modifications:<br>L_{DEN} with MCI: higher susceptibility in carriers of the APOE risk allele [OR = 1.99 (95% CI: 1.11, 3.56)] compared with others [OR = 1.21 (95% CI: 0.83, 1.78); pinter = 0.17]<br>and in participants with high $PM_{2.5}$ exposure [OR = 1.53 (95% CI: 1.17, 2.00)] compared with those exposed to low	and BMI<br>Extended analysis: CHD diagnosis, LDL cholesterol level, statin medications, anti-hypertensive medication, city of residence<br>Additional adjustments of main model with APOE ε4 and degree of depressive symptoms.<br>Two exposure models for association of noise and air

Reference (First author, publication year, S/N) Meta-analysis	Study design	Population<br>Study region<br>Sample population<br>Sample size (M, F, M+F):<br>Age (mean, range)<br>No. of cases / no. of controls or exposed/unexposed<br>Time of recruitment / follow-up (mean, range)<br>Response (%)<br>(baseline minus loss to follow-up)	Outcome<br>Disease (ICD-10)<br>Prescription<br>Questionnaire	Exposure<br>exposure source	exposure assessment	exposure levels	Results	Comments (study quality [overall assessment according to SIGN/CASP], conflict of interest [stated vs. not stated], funding [financed from public funds vs. financed from industry], confounding, strengths / weaknesses [potential bias, over- or underestimation of potential effects])
							PM2.5 [OR = 1.08 (95% CI: 0.73, 1.62); pinter = 0.17]<br>No effect modification by age (Figure S1b)<br><br>Sensitivity analysis:<br>-only non-movers: did not change the effect<br>-only objective impairment: slightly lower association<br>-Continuous noise variables (from Table S2):<br>L_{DEN} (per IQR=14.2 dB) LN (per IQR=13.6 dB)	pollution<br><br>strengths, weaknesses:<br>+ adequate definition of cases and outcome<br>+ adequate exposure measurement<br>+adequate control for confounders including air pollutants (sensitivity analyses adjusting for $PM_{2.5}$, PM_{10}, NO_2, NOx<br>-Cross-sectional study

Reference (First author, publication year, S/N) Meta-analysis	Study design	Population: Study region, Sample population, Sample size (M, F, M+F): Age (mean, range), No. of cases / no. of controls or exposed/unexposed, Time of recruitment / follow-up (mean, range), Response (%) (baseline minus loss to follow-up)	Outcome: Disease (ICD-10), Prescription, Questionnaire	Exposure: exposure source	Exposure: exposure assessment	Exposure: exposure levels	Results			Comments (study quality [overall assessment according to SIGN/CASP], conflict of interest [stated vs. not stated], funding [financed from public funds vs. financed from industry], confounding, strengths / weaknesses [potential bias, over- or underestimation of potential effects])
							Overall MCI	1.10 (0.94, 1.28)	1.09 (0.94,1.27)	with basic information about the chronology between exposition and outcome, but prevalent outcomes and prevalent covariates
							Amnestic MCI	1.15 (0.94, 1.40)	1.13 (0.93, 1.38)	
							Non-amnestic MCI	1.06 (0.87, 1.30)	1.06 (0.87, 1.30)	
							Categorical analysis of noise variables (from Table S3)			- No other noise exposure considered
							L_{DEN} (dB)	OR (95% CI)		
							<45	Reference		
							45-55	0.98 (0.73, 1.32)		
							55-65	1.08 (0.78, 1.49)		
							65-75	1.21 (0.84, 1.74)		
							> 75	1.77 (0.61, 5.07)		

Reference (First author, publication year, S/N) Meta-analysis	Study design	Population: Study region Sample population Sample size (M, F, M+F): Age (mean, range) No. of cases / no. of controls or exposed/unexposed Time of recruitment / follow-up (mean, range) Response (%) (baseline minus loss to follow-up)	Outcome: Disease (ICD-10) Prescription Questionnaire	Exposure: exposure source	Exposure: exposure assessment	Exposure: exposure levels	Results	Comments (study quality [overall assessment according to SIGN/CASP], conflict of interest [stated vs. not stated], funding [financed from public funds vs. financed from industry], confounding, strengths / weaknesses [potential bias, over- or underestimation of potential effects])
Tzivian, L. 2018, 8533<br><br>Heinz Nixdorf Recall study<br><br>!UPDATE!	Cross-sectional analysis is within cohort study		Cognitive function with Global cognitive score (GCS)<br><br>Depressive symptoms with Center for Epidemiologic Studies Depression scale (CES-D); cut-off ≥17		L_{DEN}<br>L_{DEN_IN} -> corrected for window type, bedroom location and ventilation		Fully adjusted model:<br>GCS: 10 dB noise increase<br>L_{DEN}: β=-0.34 (-0.67 - -0.01)<br>L_{DEN_IN}: β=-0.18 (-0.25 - -0.10)<br>(Adjusted for CES-D: L_{DEN}: β=-0.33 (-0.66 - -0.01); L_{DEN_IN}: β=-0.18 (-0.25 - -0.10))<br><br>Adjustment of noise for CES-D score:<br><br>L_{DEN}: OR=1.22 (0.92; 1.62)<br>L_{DEN_IN}: OR=1.04 (0.95; 1.14)	

Reference (First author, publication year, S/N) Meta-analysis	Study design	Population: Study region, Sample population, Sample size (M, F, M+F): Age (mean, range), No. of cases / no. of controls or exposed/unexposed, Time of recruitment / follow-up (mean, range), Response (%) (baseline minus loss to follow-up)	Outcome: Disease (ICD-10), Prescription, Questionnaire	Exposure: exposure source	Exposure: exposure assessment	Exposure: exposure levels	Results	Comments (study quality [overall assessment according to SIGN/CASP], conflict of interest [stated vs. not stated], funding [financed from public funds vs. financed from industry], confounding, strengths / weaknesses [potential bias, over- or underestimation of potential effects])
Van Kamp, I. 2007, H-2885, Netherlands<br>Yes	Longitudinal / cross-sectional study + panel study	Study region: Amsterdam Schiphol International airport, The Netherlands<br><br>Sample population: Residents living on a radius of 25km around airport plus northern area, sampling not specified.<br><br>Sample size: Baseline 2002: M+F = 5873 participants	Physiological well being Measured by GHQ-12 (Dutch version)<br><br>Intake of prescribed sleeping pills or sedatives in the past 2 weeks<br><br>Intake of anti-depressants in the past 2	Aircraft	L_{DEN}<br>L_{N}<br><br>6 months average for 2002-2005 National Aerospace Laboratory (NLR) for an area of 55 by 71 km	NA<br><br>No starting point specified	Cross-sectional surveys:<br><br>Self-reported mental health complaint and aircraft noise (from Table 2)<br>L_{DEN} + L_{N}: expressed as OR per 3 dB<br><br>Intake of prescribed sleeping medication or sedatives in the past 2 weeks:<br>Exposure / 2002 / 2005<br>L_{DEN} / 0.91 (0.75-1.09) / 1.06 (0.89-1.26)	study quality:<br>-<br>(Longitudinal study), Cross-sectional and panel analysis<br><br>conflict of interest: not stated<br><br>funding: not stated<br><br>confounding (adjusted for): gender, age, ethnicity,

Reference (First author, publication year, S/N) Meta-analysis	Study design	Population<br>Study region<br>Sample population<br>Sample size (M, F, M+F):<br>Age (mean, range)<br>No. of cases / no. of controls or exposed/unexposed<br>Time of recruitment / follow-up (mean, range)<br>Response (%)<br>(baseline minus loss to follow-up)	Outcome<br>Disease (ICD-10)<br>Prescription<br>Questionnaire	Exposure<br>exposure source	exposure assessment	exposure levels	Results	Comments (study quality [overall assessment according to SIGN/CASP], conflict of interest [stated vs. not stated], funding [financed from public funds vs. financed from industry], confounding, strengths / weaknesses [potential bias, over- or underestimation of potential effects])
		2005:<br>Survey (from 2002 and new participants 2005)<br>M+F= 6091<br>Longitudinal study:<br>M+F about 2,700<br><br>Additional panel study:<br>M+F = 640 participants<br><br>Age:<br>≥ 18 years<br><br>Exposed/unexposed:<br>Surveys:<br>NA	weekds				L_N 0.94 (0.82-1.08) 1.08 (0.91-1.27)<br>Intake of prescribed anti-depressives in the past 2 weeks:<br>Exposure 2002 2005<br>L_{DEN} 1.00 (0.84-1.20) -<br>L_N 1.04 (0.86-1.26) -<br>*-: no results available, statistical models do not converge*<br><br>GHQ 12 (2 or more complaints):<br>Exposure 2002 2005<br>L_{DEN} 1.03 (0.94-1.12) 0.94 (0.84-1.05)	social economic status, smoking, alcohol, BMI, household size, level of urbanization<br><br>strengths/ weaknesses:<br>+ logistic regression model, stratification<br>+ panel study<br>+ included non-response<br>- no information on sampling<br>- no information on

Reference (First author, publication year, S/N) Meta-analysis	Study design	Population: Study region Sample population Sample size (M, F, M+F): Age (mean, range) No. of cases / no. of controls or exposed/unexposed Time of recruitment / follow-up (mean, range) Response (%) (baseline minus loss to follow-up)	Outcome: Disease (ICD-10) Prescription Questionnaire	Exposure: exposure source	Exposure: exposure assessment	Exposure: exposure levels	Results			Comments (study quality [overall assessment according to SIGN/CASP], conflict of interest [stated vs. not stated], funding [financed from public funds vs. financed from industry], confounding, strengths / weaknesses [potential bias, over- or underestimation of potential effects])
		Panel Study:<br>Control group (less than 1.5 dB increase or decrease expected)<br>Exposure group I: increase in L_{DEN} ≥1.5 dB<br>Exposure group II: decrease in L_{DEN} ≥1.5 dB<br><br>Time of recruitment/ follow-up: Baseline survey: 2002 (baseline) and 2005 (follow-up)<br><br>Opening of a new fifth					L_N	1.02 (0.94-1.10)	0.98 (0.89-1.09)	the distribution of gender<br>- no information on age<br>- mix of old and new respondents 2005<br>- air particulate level not considered<br>- no information of noise exposure (mean, range)<br>- No other noise exposure considered
							Longitudinal study:<br>Change in prevalence of mental health complaint to the change in noise levels from 2002 till 2005 (N≈2700)<br>→ change in L_{DEN} and L_N over 3 years did not statistically significant affect the prevalence of any of the mental health complaints<br>→ all OR were close to 1, varying between 0.95 and 1.03 per 3dB noise level change (OR adjusted for potential confounding and current			

Reference (First author, publication year, S/N) Meta-analysis	Study design	Population<br>Study region<br>Sample population<br>Sample size (M, F, M+F):<br>Age (mean, range)<br>No. of cases / no. of controls or exposed/unexposed<br>Time of recruitment / follow-up (mean, range)<br>Response (%)<br>(baseline minus loss to follow-up)	Outcome<br>Disease (ICD-10)<br>Prescription<br>Questionnaire	Exposure<br>exposure source	exposure assessment	exposure levels	Results	Comments (study quality [overall assessment according to SIGN/CASP], conflict of interest [stated vs. not stated], funding [financed from public funds vs. financed from industry], confounding, strengths / weaknesses [potential bias, over- or underestimation of potential effects])
		runway: 2003<br><br>Panel study:<br>2002-2005 (yearly basis)<br><br>Response rate:<br>Baseline survey:<br>2002: 46 %<br>Follow-up:<br>2005:<br>2671/4435=73% (of the re-invited) +<br>3420/8400=43 % (new subjects)<br>Total N 2005=6091<br>Panel study:<br>64 %					noise levels)<br><br>Panel Study:<br>- no relationship between noise and GHQ 12 scores<br>- intake of prescribed sleeping medications/ sedatives and anti-depressives had too little variations in time for a statistical analysis	

Reference (First author, publication year, S/N) Meta-analysis	Study design	Population: Study region Sample population Sample size (M, F, M+F): Age (mean, range) No. of cases / no. of controls or exposed/unexposed Time of recruitment / follow-up (mean, range) Response (%) (baseline minus loss to follow-up)	Outcome: Disease (ICD-10) Prescription Questionnaire	Exposure: exposure source	Exposure: exposure assessment	Exposure: exposure levels	Results	Comments (study quality [overall assessment according to SIGN/CASP], conflict of interest [stated vs. not stated], funding [financed from public funds vs. financed from industry], confounding, strengths / weaknesses [potential bias, over- or underestimation of potential effects])
Zock, 2018, 8499	Cross-sectional, (using prospective panel data on neighborhood social cohesion)	Study region: Netherlands (neighborhoods with the same five-digit postal code)<br><br>Sample size: M+F=4,450 M= 2,184, F=2,266<br><br>Sample population: Sample based on NIVEL primary care database (10% of GPs are registered). Included were all individuals who had been living in the area during all 12 months in 2013.	Disease in 2013 defined according to the ICPC code (International Classification of Primary Care)<br><br>Depression P03 = Feeling depressed P76 = Depressive disorder<br><br>Anxiety P01 = Feeling	Road 2008<br><br>Railway 2007	Modelled -> Standard Model Instrumentation for Noise Assessments (STAMINA) 10x10m spatial resolution (residence matched to nearest grid point)<br><br>L_{DEN}	-	Prevalence Depression: n = 202 (4.5%) Anxiety: n = 178 (4.0%)<br><br>Road: Per 10 dB Depression: OR = 1.17, 95% CI 0.72-1.91 Anxiety: OR = 0.94, 95% CI 0.59-1.52<br><br>Railway: Per 10 dB Depression: OR = 0.94, 95% CI 0.76-1.18 Anxiety: OR = 1.06, 95% CI 0.87-1.29	study quality: + (to -) Cross-sectional analysis conflict of interest: stated funding: stated confounding (controlled for): sex, age, household income and socio-economic status. strengths, weaknesses: -Cross-sectional

Reference (First author, publication year, S/N) Meta-analysis	Study design	Population<br>Study region<br>Sample population<br>Sample size (M, F, M+F):<br>Age (mean, range)<br>No. of cases / no. of controls or exposed/unexposed<br>Time of recruitment / follow-up (mean, range)<br>Response (%)<br>(baseline minus loss to follow-up)	Outcome<br>Disease (ICD-10)<br>Prescription<br>Questionnaire	Exposure<br>exposure source	exposure assessment	exposure levels	Results	Comments (study quality [overall assessment according to SIGN/CASP], conflict of interest [stated vs. not stated], funding [financed from public funds vs. financed from industry], confounding, strengths / weaknesses [potential bias, over- or underestimation of potential effects])
		Age: mean 40.5 years<br>0-4 years = 233<br>5-12 years = 461<br>13-18 years = 328<br>19-39 years = 1032<br>40-64 years = 1663<br>65 years and older = 733<br><br>No. of cases<br>See results<br><br>Time of recruitment<br>2013<br><br>Response:	anxious/ nervous/ tense<br>P74 = Anxiety disorder/ anxiety state<br><br>*Chronic disease in 2011 and 2012 "taken into account to minimize misclassification".*					analysis with longitudinal data on neighborhood social cohesion, and retrospective data on noise exposure providing basic information about chronology between exposition and outcome<br>-air pollution, green spaces and urbanity were estimated but not adjusted for in the model

Reference (First author, publication year, S/N) Meta-analysis	Study design	Population: Study region<br>Sample population<br>Sample size (M, F, M+F):<br>Age (mean, range)<br>No. of cases / no. of controls or exposed/unexposed<br>Time of recruitment / follow-up (mean, range)<br>Response (%)<br>(baseline minus loss to follow-up)	Outcome: Disease (ICD-10)<br>Prescription<br>Questionnaire	Exposure: exposure source	Exposure: exposure assessment	Exposure: exposure levels	Results	Comments (study quality [overall assessment according to SIGN/CASP], conflict of interest [stated vs. not stated], funding [financed from public funds vs. financed from industry], confounding, strengths / weaknesses [potential bias, over- or underestimation of potential effects])
		Secondary data						+ adequate definition and assessment of outcome<br>+adequate control for confounders<br>+ adequate exposure assessment<br>+ routine data, should lead to reduced selection bias

2. Studies excluded with reasons

Table 2. Studies excluded with reasons.

**Outcome not (clinical) mental disorder (n = 101)**
1. Al-Mutairi, N. Z., Al-Attar, M. A., & Al-Rukaibi, F. S. (2011). Traffic-generated noise pollution: exposure of road users and populations in Metropolitan Kuwait. *Environmental Monitoring & Assessment*, *183*(1-4), 65-75. doi: 10.1007/s10661-011-1906-0
2. Arbeitsgemeinschaft für sozio-psychologische Fluglärmuntersuchungen (1973): Untersuchungen über den Fluglärm und seine Wirkungen im Gebiet von drei Schweizer Zivilflughäfen 1971/72. Eidgenössisches Luftamt, Bundeshaus, Bern, Schweiz.
3. Babisch, W., Schulz, C., Seiwert, M., & Conrad, A. (2012). Noise annoyance as reported by 8-to 14-year-old children. *Environment &Behavior*, *44*(1), 68-86. doi: 10.1177/0013916510387400
4. Barceló, M. A., Varga, D., Tobias, A., Diaz, J., Linares, C., & Saez, M. (2016). Long term effects of traffic noise on mortality in the city of Barcelona, 2004–2007. *Environmental Research*, *147*, 193-206. doi: 10.1016/j.envres.2016.02.010
5. Basner, M., & Samel, A. (2004). Nocturnal aircraft noise effects. *Noise and Health*, *6*(22), 83.
6. Bättig, K. & Buzzi, R. (1979). Psychophysiological effects of aircraft noise. *Activitas Nervosa Superior*, 21(4), 257-258.
7. Bättig, K., Zeier, H., Müller, R., & Buzzi, R. (1980). A field study on vegetative effects of aircraft noise. *Archives of Environmental Health: An International Journal*, *35*(4), 228-235.
8. Bättig, K., & Buzzi, R. (1981). Psychophysiologische Effekte von Lärm und Beschäftigung in der Heimsituation. *Zeitschrift für Experimentelle und Angewandte Psychologie*, *28*, 1-14.
9. Bättig, K., & Buzzi, R. (1981). Psychophysiological effects of noise and activity in the home situation. *Zeitschrift für Experimentelle und Angewandte Psychologie*, *28*, 1-14.
10. Bättig, K., & Buzzi, R. (1982). Physiological responses to noise and to the type of activity under field conditions. *Activitas Nervosa Superior*, (Pt 1), 236-240.
11. Baumbach, W., Mörstedt, R., Schulze, B., Wölke, G., Ullmann, R., & Grossmann, G. (1990). New aspects of the traffic noise problem in the inner city area. *Zeitschrift für die gesamte Hygiene und ihre Grenzgebiete*, *36*(4), 204-206.
12. Beutel, M. E., Jünger, C., Klein, E. M., Wild, P., Lackner, K., Blettner, M., ... & Münzel, T. (2016). Noise annoyance is associated with depression and anxiety in the general population-the contribution of aircraft noise. *Plos one*, *11*(5), e0155357. doi: 10.1371/journal.pone.0155357
13. Birnie, S. E., Hall, F. L., & Taylor, S. M. (1980). Community response to noise from a general aviation airport. *Noise Control Eng.;(United States)*, *15*.
14. Björk, J., Ardö, J., Stroh, E., Lövkvist, H., Östergren, P. O., & Albin, M. (2006). Road traffic noise in southern Sweden and its relation to annoyance, disturbance of daily activities and health. *Scandinavian Journal of Work, Environment & Health*, 32(5), 392-401. doi:10.5271/sjweh.1035
15. Björk, J., Ardö, J., Stroh, E., Lövkvist, H., Östergren, P. O., & Albin, M. (2007). Erratum: Road traffic noise in southern Sweden and its relation to annoyance, disturbance of daily activities and health. *Scandinavian Journal of Work, Environment & Health*, 33(1), 392-401.
16. Black, D. A., Black, J. A., Issarayangyun, T., & Samuels, S. E. (2007). Aircraft noise exposure and resident's stress and hypertension: A public health perspective for airport environmental management. *Journal of Air Transport Management*, *13*(5), 264-276. doi:

10.1016/j.jairtraman.2007.04.003

17. Bodenheim, A. & Unger, M (1991). Psychosoziale Auswirkungen des Wohnens an hochfrequentierten Straßen : Eine Erkundungsstudie. *Unser Doppelleben. Neue Studien zur Umweltbetroffenheit*, 103-139.
18. Botteldooren, D., Dekoninck, L., & Gillis, D. (2011). The influence of traffic noise on appreciation of the living quality of a neighborhood. *International Journal of Environmental Research and Public Health*, *8*(3), 777-798. doi: 10.3390/ijerph8030777
19. Brink, M. (2011). Parameters of well-being and subjective health and their relationship with residential traffic noise exposure—A representative evaluation in Switzerland. *Environment International*, *37*(4), 723-733. doi: 10.1016/j.envint.2011.02.011.
20. Brown, A. L. (1984). Critique of subjective responses of Chinese to aircraft noise. *Applied Acoustics*, *17*(3), 223-232.
21. Bullinger, M., & Bahner, U. (1997). Erlebte Umwelt und subjektive Gesundheit. Eine Untersuchung an Müttern und Kindern aus unterschiedlichen lärmbelasteten Gebieten. *Zeitschrift fur Gesundheitwissenschaften*, *3*, 89-108.
22. Bullinger, M., Hygge, S., Evans, G. W., Meis, M., & Mackensen, S. V. (1999). The psychological cost of aircraft noise for children. *Zentralblatt für Hygiene und Umweltmedizin*, *202*(2-4), 127-138. doi: 10.1080/00207594.2013.804190
23. Bullinger, M. (2000). Wahrgenommene Umweltbelastung und gesundheitsbezogene Lebensqualität von Müttern und Kindern : Eine umweltepidemiologische Untersuchung an Familien aus unterschiedlich fluglärmbelasteten Gebieten in Bayern. *Lebensqualitätsforschung aus medizinpsychologischer und -soziologischer Perspektive*, 354-367.
24. Buzzi, R., & Bättig, K. (1984). Extraaurale beziehungsweise vegetative Effekte von Umweltlärm. *Beiträge zur Bedeutungslehre des Schalls. Peter Lang Verlag, Bern*, 243-251.
25. Carter, N. L. (1996). Transportation noise, sleep, and possible after-effects. *Environment International*, *22*(1), 105-116. doi: 10.1016/0160-4120(95)00108-5
26. Clark, C. & Sörqvist, P. (2012). The influence of noise on performance and behavior. *Noise Health*, 14, 292–296.
27. Conzelmann-Auer, C., Braun-Fahrländer, C., Ackermann-Liebrich, U., & Wanner, H. U. (1993). Perception of traffic noise emission in Basel city canton in comparison to actually measured noise levels. *Sozial-und Präventivmedizin*, *38*(4), 231-238. doi: 10.1007/BF01624541
28. De Jong, R. G. (1979). A Dutch study on railroad traffic noise. *Journal of Sound and Vibration*, *66*(3), 497-502. doi: 10.1016/0022-460X(79)90871-X
29. De Jong, R. G. (1981). Dutch study of noise disturbance due to airplanes. *Geluid Omgeving*, *4*(4), 228-232.
30. De Kluizenaar, Y. , Salomons, E. M., Janssen, S. A., van Lenthe, F. J., Vos, H., Zhou, H., ... & Mackenbach, J. P. (2011). Urban road traffic noise and annoyance: The effect of a quiet façade. *The Journal of the Acoustical Society of America*, *130*(4), 1936-1942. doi: 10.1121/1.3621180.
31. de Paiva Vianna, K. M., Cardoso, M. R. A., & Rodrigues, R. M. C. (2015). Noise pollution and annoyance: An urban soundscapes study. *Noise & Health*, *17*(76), 125. doi: 10.4103/ 1463-1741.155833
32. Delauzun, F. R., & Griffiths, I. D. (1978). The problem of individual differences in sensitivity to traffic noise and the establishment of standards. *International Review of Applied Psychology*. doi:

10.1111/j.1464-0597.1978.tb00354.x

33. Dratva, J., Zemp, E., Dietrich, D. F., Bridevaux, P. O., Rochat, T., Schindler, C., & Gerbase, M. W. (2010). Impact of road traffic noise annoyance on health-related quality of life: Results from a population-based study. *Quality of Life Research*, *19*(1), 37-46. doi: 10.1007/s11136-009-9571-2
34. Evans, G. W., Hygge, S., & Bullinger, M. (1995). Chronic noise and psychological stress. *Psychological Science*, *6*(6), 333-338. doi: 10.1111/j.1467-9280.1995.tb00522.x
35. Evans, G. W., Bullinger, M., & Hygge, S. (1998). Chronic noise exposure and physiological response: A prospective study of children living under environmental stress. *Psychological Science*, *9*(1), 75-77. doi: 10.1111/1467-9280.00014
36. Fiedler, F. E., & Fiedler, J. (1975). Port noise complaints: Verbal and behavioral reactions to airport-related noise. *Journal of Applied Psychology*, *60*(4), 498. Doi: 10.1037/h0076924
37. Findeis, H., & Peters, E. (2004). Disturbing effects of low frequency sound immissions and vibrations in residential buildings. *Noise and Health*, *6*(23), 29.
38. Fooladi, M. M. (2012). Involuntary and persistent environmental noise influences health and hearing in Beirut, Lebanon. *Journal of Environmental and Public Health*, 2012: 235618. doi: 10.1155/2012/235618
39. Franssen, E. A. M., Van Wiechen, C. M. A. G., Nagelkerke, N. J. D., & Lebret, E. (2004). Aircraft noise around a large international airport and its impact on general health and medication use. *Occupational and Environmental Medicine*, *61*(5), 405-413. doi: 10.1136/oem.2002.005488
40. Furlan, P. M., Bergamini, L. & Bergamasco, B. (1976). Street noise. A psychologic and neurophysiologic study. *Rassegna Di Studi Psichiatrici*, *65*: 519-530.
41. Gómez-Jacinto, L., & Moral-Toranzo, F. (1999). Urban traffic noise and self-reported health. *Psychological Reports*, *84*(3_suppl), 1105-1108. doi:10.2466/pr0.1999.84.3c.1105
42. Goswami, S., Nayak, S. K., Pradhan, A. C., & Dey, S. K. (2011). A study on traffic noise of two campuses of University, Balasore, India. *Journal of Environmental Biology*, *32*(1), 105-109.
43. Gozalo, G. R., & Barrigón Morillas, J. M. (2016). Analysis of sampling methodologies for noise pollution assessment and the impact on the population. *International Journal of Environmental Research and Public Health*, *13*(5), 490. doi: 10.3390/ijerph13050490
44. Graeven, D. B. (1974). The effects of airplane noise on health: An examination of three hypotheses. *Journal of Health and Social Behavior*, *15*(4), 336-343. doi: 10.2307/2137094
45. Greiser, E. (2014). Health risk railroad noise-prognosis of potential health risks subsequent to night-time exposure to railroad noise in the German part of the Transversal Rotterdam Genova. *Gesundheitswesen (Bundesverband der Ärzte des Öffentlichen Gesundheitsdienstes (Germany))*, *76*(12), 862-864. doi: 10.1055/s-0034-1395639
46. Gundersen, H., Magerøy, N., Moen, B. E., & Bråtveit, M. (2013). Traffic density in area of residence is associated with health-related quality of life in women, the community-based Hordaland Health Study. *Archives of Environmental & Occupational Health*, *68*(3), 153-160. doi: 10.1080/19338244.2012.676103
47. Haines, M. M., Brentnall, S. L., Stansfeld, S. A., & Klineberg, E. (2003). Qualitative responses of children to environmental noise. *Noise and Health*, *5*(19), 19.
48. Hammad, R. N. S., & Abdelazeez, M. K. (1987). Measurements and analysis of the traffic noise in Amman, Jordan and its effects. *Applied Acoustics*, *21*(4), 309-320. doi: 10.1016/0003-682X(87)90052-1

49. Hammersen, F., Niemann, H., & Hoebel, J. (2016). Environmental noise annoyance and mental health in adults: findings from the cross-sectional German Health Update (GEDA) Study 2012. *International Journal of Environmental Research and Public Health*, *13*(10), 954. doi:10.3390/ijerph13100954
50. Han, Z. X., Lei, Z. H., Zhang, C. L., Xiong, W., Gan, Z. L., Hu, P., & Zhang, Q. B. (2015). Noise monitoring and adverse health effects in residents in different functional areas of Luzhou, China. *Asia Pacific Journal of Public Health*, *27*(2_suppl), 93S-99S. doi: 10.1177/1010539514560056
51. Hawkins, M. M., & Large, J. B. (1983). Subjective response to noise in rural villages, particularly from road traffic. *Journal of Sound and Vibration*, *88*(3), 321-331. doi: 10.1016/0022-460X(83)90691-0
52. Héritier, H., Vienneau, D., Frei, P., Eze, I. C., Brink, M., Probst-Hensch, N., & Röösli, M. (2014). The association between road traffic noise exposure, annoyance and health-related quality of life (HRQOL). *International Journal of Environmental Research and Public Health*, *11*(12), 12652-12667. doi: 10.3390/ijerph111212652
53. Honold, J. & van der Meer, E. (2017). Residential exposure to multiple environmental burdens and health: A mixed-methods study**.** *Umweltpsychologie* 21(1), 119-138.
54. Jarup, L., Dudley, M. L., Babisch, W., Houthuijs, D., Swart, W., Pershagen, G., ... & Vigna-Taglianti, F. (2005). Hypertension and exposure to noise near airports (HYENA): study design and noise exposure assessment. *Environmental Health Perspectives*, *113*(11), 1473. doi:10.1289/ehp.8037
55. Jenkins, M. A., & Pahl, J. (1975). Measurement of freeway noise and community response. *The Journal of the Acoustical Society of America*, *58*(6), 1222-1231. doi: 10.1121/1.380804
56. Karsdorf, G., & Klappach, H. (1968). Einflüsse des Verkehrslärms auf Gesundheit und Leistung bei Oberschülern einer Großstadt. *Zeitschrift für die Gesamte Hygiene und ihre Grenzgebiete*, *14*, 52-54.
57. Kastka, J. (1984). Untersuchungen zur Belästigung durch Verkehrslärm: umweltpsychiologische Analysen zu einem umwelthygienischen Konzept. Dissertation Universität Düssldorf.
58. Klatte, M., Spilski, J., Mayerl, J., Möhler, U., Lachmann, T., & Bergström, K. (2017). Effects of aircraft noise on reading and quality of life in primary school children in Germany: Results from the NORAH study. *Environment and Behavior*, *49*(4), 390-424. doi: 10.1177/0013916516642580
59. Knipschild, P. (1977). V. Medical effects of aircraft noise: community cardiovascular survey. *International Archives of Occupational and Environmental Health*, *40*(3), 185-190. doi : 10.1007/BF01842081
60. Ko, J. H., Chang, S. I., Kim, M., Holt, J. B., & Seong, J. C. (2011). Transportation noise and exposed population of an urban area in the Republic of Korea. *Environment International*, *37*(2), 328-334. doi: 10.1016/j.envint.2010.10.001
61. Koszarny, Z. (2001). The estimate of well-being and self-assessed health status in urban population in various acoustic areas. *Roczniki Panstwowego Zakladu Higieny*, *52*(2), 165-178.
62. Koszarny, Z., Szata, W., & Goryński, P. (1979). Exposure to railway noise and its effect on the population. *Roczniki Panstwowego Zakladu Higieny*, *30*(4), 387-395.
63. Kryter, K. D. (2009). Acoustical model and theory for predicting effects of environmental noise

on people. *The Journal of the Acoustical Society of America*, *125*(6), 3707-3721. doi: 10.1121/1.3125320

64. Krert, E. (1986). Entstehung und Bewältigung von Lärmbelastungen - insbesondere Fluglärmbelastungen - bei Kindern. Dissertation Universität Graz.
65. La Torre, G., Moscato, U., La Torre, F., Ballini, P., Marchi, S., & Ricciardi, W. (2007). Environmental noise exposure and population health: a cross-sectional study in the Province of Rome. *Journal of Public Health*, *15*(5), 339-344. doi: 10.1007/s10389-007-0144-y
66. Lercher, P., & Kofler, W. W. (1996). Behavioral and health responses associated with road traffic noise exposure along alpine through-traffic routes. *Science of the Total Environment, 189-190*, 85-89 doi: 10.1016/0048-9697(96)05194-7
67. Lercher, P., Evans, G. W., Meis, M., & Kofler, W. W. (2002). Ambient neighbourhood noise and children's mental health. *Occupational and environmental medicine*, *59*(6), 380-386. doi: 10.1136/oem.59.6.380
68. Lefèvre, M., Carlier, M. C., Champelovier, P., Lambert, J., Laumon, B., & Evrard, A. S. (2017). Effects of aircraft noise exposure on saliva cortisol near airports in France. *Occupational Environmental Medicine*, oemed-2016. doi: 10.1136/oemed-2016-104208
69. Maschke, C. (2003). Epidemiological research on stress caused by traffic noise and its effects on high blood pressure and psychic disturbances. In *Proceedings of the 8th International Congress on Noise as a Public Health Problem (Jong RD, Houtgast T, Franssen EAM, Hofman W, eds). Shiedam, the Netherlands: Foundation ICBEN* (pp. 96-101).
70. Matheson, M. P., Stansfeld, S. A., & Haines, M. M. (2003). The effects of chronic aircraft noise exposure on children's cognition and health: 3 field studies. *Noise and Health*, *5*(19), 31.
71. Meister, E. A., & Donatelle, R. J. (2000). The impact of commercial-aircraft noise on human health: a neighborhood study in metropolitan Minnesota. *Journal of Environmental Health*, *63*(4), 9.
72. Mosskov, J. I., & Ettema, J. H. (1977). IV. Extra-auditory effects in long-term exposure to aircraft and traffic noise. *International Archives of Occupational and Environmental Health*, *40*(3), 177-184. doi: 10.1007/BF01842080
73. Müller, R. (1980). Does aircraft noise affect health? *Sozial-und Präventivmedizin*, *25*(3), 103-109. doi: 1007/BF02074972
74. Noack, R. H. (1995). *Lärm und Ruhe: Untersuchungen zur Belästigung durch Verkehrsgeräusche und Pegelschwankungen*. dis's' kurs.
75. Pedersen, E. (2015). City dweller responses to multiple stressors intruding into their homes: Noise, light, odour, and vibration. *International Journal of Environmental Research and Public Health*, *12*(3), 3246-3263. doi: 10.3390/ijerph120303246
76. Pulles, M. P. J., Biesiot, W., & Stewart, R. (1990). Adverse effects of environmental noise on health: an interdisciplinary approach. *Environment International*, *16*(4-6), 437-445. doi: 10.1016/0160-4120(90)90012-U
77. Relster, E. (1977). Influence of traffic noise *on* mental health. *Ugeskr Laeger*, 139 (9):549-552.
78. Rilfatti, M. (1982). Human reactions to community noise. I. Noise of urban street traffic in the city of Verona. *Nuovi Annali d'Igiene e Microbiologia*, *33*(4-6), 799-815.
79. Röösli, M., Vienneau, D., & Perez, L. (2015). Transportation noise:'neglected exposure'on a global scale. *Tropical Medicine & International Health*, *20*, 4.

80. Schreckenberg, D., & Meis, M. (2006). Effects of aircraft noise on noise annoyance and quality of life around Frankfurt Airport. Final abridged report
81. Schreckenberg, D., Griefahn, B., & Meis, M. (2010). The associations between noise sensitivity, reported physical and mental health, perceived environmental quality, and noise annoyance. *Noise and Health*, *12*(46), 7. doi: 10.4103/1463-1741.59995.
82. Schreckenberg, D., Meis, M., Kahl, C., Peschel, C., & Eikmann, T. (2010). Aircraft noise and quality of life around Frankfurt Airport. *International Journal of Environmental Research and Public Health*, *7*(9), 3382-3405. doi: 10.3390/ijerph7093382
83. Seabi, J. (2013). An epidemiological prospective study of children's health and annoyance reactions to aircraft noise exposure in South Africa. *International Journal of Environmental Research and Public Health*, *10*(7), 2760-2777. doi: 10.3390/ijerph10072760
84. Seidman, M. D., & Standring, R. T. (2010). Noise and quality of life. *International Journal of Environmental Research and Public Health*, *7*(10), 3730-3738. doi: 10.3390/ijerph7103730
85. Shepherd, D., Dirks, K., Welch, D., McBride, D., & Landon, J. (2016). The covariance between air pollution annoyance and noise annoyance, and its relationship with health-related quality of life. *International Journal of Environmental Research and Public Health*, *13*(8), 792. doi: 10.3390/ijerph13080792
86. Stansfeld, S., Hygge, S., Clark, C., & Alfred, T. (2010). Night time aircraft noise exposure and children's cognitive performance. *Noise and Health*, *12*(49), 255. doi: 10.4103/1463-1741.70504.
87. Stansfeld, S. A., & Shipley, M. (2015). Noise sensitivity and future risk of illness and mortality. *Science of the Total Environment*, *520*, 114-119. doi: 10.1016/j.scitotenv.2015.03.053.
88. Tzivian, L., Foraster, M., Hennig, F.,Fuks, K., Sugiri, D.,Erbel, R., Jockel, H. K., Moebus, S.& Hoffmann, B. (2016). Long-term traffic noise exposure corrected for indoors and its association with outdoor air pollution and annoyance in participants of the Heinz Nixdorf recall study. *European Journal of Epidemiology*, 31, S214-S215. doi: 10.1289/ehp.1509824
89. Urban, J., & Máca, V. (2013). Linking traffic noise, noise annoyance and life satisfaction: A case study. *International Journal of Environmental Research and Public Health*, *10*(5), 1895-1915. doi: 10.3390/ijerph10051895
90. van den Berg, F., Verhagen, C., & Uitenbroek, D. (2015). The relation between self-reported worry and annoyance from air and road traffic. *International Journal of Environmental Research and Public Health*, *12*(3), 2486-2500. doi: 10.3390/ijerph120302486
91. van Kempen, E., van Kamp, I., Nilsson, M., Lammers, J., Emmen, H., Clark, C., & Stansfeld, S. (2010). The role of annoyance in the relation between transportation noise and children's health and cognition. *The Journal of the Acoustical Society of America*, *128*(5), 2817-2828. doi: 10.1121/1.3483737
92. Van Wiechen, C. M., Franssen, E. A., de Jong, R. G., & Lebret, E. (2002). Aircraft noise exposure from Schiphol airport: a relation with complainants. *Noise and Health*, *5*(17), 23.
93. Wang, R., Helbich, M., Yao, Y., Zhang, J., Liu, P., Yuan, Y., & Liu, Y. (2019). Urban greenery and mental wellbeing in adults: Cross-sectional mediation analyses on multiple pathways across different greenery measures. *Environmental research*, *176*, 108535. 10.1016/j.envres.2019.108535
94. Ward, L. M., & Suedfeld, P. (1973). Human responses to highway noise. *Environmental Research*, *6*(3), 306-326. doi: 10.1016/0013-9351(73)90043-1

95. Waters, D. M. (1979). Overall railway noise impact in the UK. *Journal of Sound and Vibration*, *66*(3), 477-481 doi: 10.1016/0022-460X(79)90868-X
96. Welch, D., Shepherd, D., Dirks, K. N., McBride, D., & Marsh, S. (2013). Road traffic noise and health-related quality of life: A cross-sectional study. *Noise and Health*, *15*(65), 224. doi: 10.4103/1463-1741.113513.
97. Wothge, J., Belke, C., Möhler, U., Guski, R., & Schreckenberg, D. (2017). The Combined Effects of Aircraft and Road Traffic Noise and Aircraft and Railway Noise on Noise Annoyance—An Analysis in the Context of the Joint Research Initiative NORAH. *International Journal of Environmental Research and Public Health*, *14*(8), 871. doi: 10.3390/ijerph14080871.
98. Wu, J., Zou, C., He, S., Sun, X., Wang, X., & Yan, Q. (2019). Traffic noise exposure of high-rise residential buildings in urban area. *Environmental Science and Pollution Research*, *26*(9), 8502-8515. doi: 10.1007/s11356-019-04640-1
99. Yokoo, Y., & Mitani, Y. (1982). The effects of chronic exposure to train noise on the mental efficiency of elementary school children (report 2). *Nippon Eiseigaku Zasshi (Japanese Journal of Hygiene)*, *37*(5), 753-761. doi: 10.1265/jjh.37.753
100. Yoshida, T., & Nakamura, S. (1988). Subjective ratings of health status and railway noise. *Journal of Sound and Vibration*, *127*(3), 593-598. doi: 10.1016/0022-460X(88)90388-4
101. Zijlema, W. L., Morley, D. W., Stolk, R. P., & Rosmalen, J. G. M. (2015). Noise and somatic symptoms: A role for personality traits?. *International Journal of Hygiene and Environmental Health*, *218*(6), 543-549. doi: 0.1016/j.ijheh.2015.05.001

Reviews/ Letters/ Editorials (n = 51)

1. Adams, L. (2017). Dementia: Could heavy traffic drive dementia risk?. *Nature Reviews Neurology*, *13*(3), 128. doi: 10.1038/nrneurol.2017.6
2. Anonymous (1979). Minds under the flightpath. *Lancet*, 2(8156-8157), 1343.
3. Anonymous (1992). Health effects of the noise of low-flying aircrafts. [German]. *Zeitschrift für Lärmbekämpfung*, 39(2), 52-54.
4. Anonymous (2002). Noise hurts boys and girls. *Environmental Health Perspectives,* 110(10), A569.
5. Babisch, W. (1985). Danger to health caused by environmental noise?. *Schriftenreihe des Vereins für Wasser-, Boden-und Lufthygiene*, *65*, 425.
6. Babisch, W. (2000). Health effects of traffic noise. [German]. *Schriftenreihe des Vereins für Wasser-, Boden-und Lufthygiene*, 106, 178-192.
7. Babisch, W. (2004). Health aspects of extra-aural noise research. *Noise and Health*, *6*(22), 69.
8. Björklid, P. (1994). Children-traffic-environment. *Architecture & Comportement*, *10*(4), 361-369.
9. Bröer, C. (2007). Aircraft noise and risk politics. *Health, Risk & Society*, *9*(1), 37-52. https://doi.org/10.1080/13698570601181631
10. Camard, J. P., Lefranc, A., Gremy, I., & Ferry, R. (2004). Noise-induced health effects: Recent epidemiological studies. *Environnement, Risques & Santé*, *3*(4), 235-242.
11. Cho, H. W. & Chu, C. (2014). Sound in the air. Osong Public Health and Research Perspectives, 5(2): 75-76. doi: 10.1016/j.phrp.2014.04.001
12. Chowns, R. H., Abey-Wickrama, I., A'Brook, M. F., Gattoni, F. E. G., & Herridge, C. F. (1970). Mental-hospital admissions and aircraft noise. *The Lancet*, *295*(7644), 467-468. doi: 10.1016/S0140-6736(70)90858-5
13. Dalle, B. (1966). Troubles du comportement et vie dans les nouveaux ensembles urbains.

L'Évolution Psychiatrique.
14. Dora, C., & Phillips, M. (Eds.). (2000). *Transport, environment and health* (No. 89). WHO Regional Office Europe.
15. Esser, A. H. (1974). Environment and mental health. *Science, Medicine and Man*, *1*(3), 181-193.
16. Evans, G. W. (2003). The built environment and mental health. *Journal of Urban Health*, *80*(4), 536-555. doi:10.1093/jurban/jtg063
17. Gottlob, D. & Meurers, H. (1984). Effects of road traffic noise. Umweltpolitik 7 (1), 41-59.
18. Haines, M., & Stansfeld, S. (2003). Ambient neighbourhood noise and children's mental health. *Occupational and Environmental Medicine*, *60*(2), 146-146. doi: 10.1136/oem.60.2.146
19. Health Council of the Netherlands: Committee on the Health Impact of Large Airports. Public Health
20. Herridge, C. F. (1974). Aircraft noise and mental health. *Journal of Psychosomatic Research*, *18*(4), 239-243. doi: 10.1016/0022-3999(74)90004-X
21. Impact of Large Airports. The Hague: Health Council of the Netherlands, 1999 (1999/14E).
22. Ising, H., Babisch, W., & Kruppa, B. (1998). Ergebnisse epidemiologischer Forschung im Bereich Lärm. In Gesundheitsrisiken durch Lärm-Tagungsband zum Symposium Veranstaltungen im Rahmen der Initiative „Schritte zu einer nachhaltigen, umweltgerechten Entwicklung". Wissenschaftszentrum Bonn, 10. Februar 1998.
23. Jansen, G. (1986). Zur „erheblichen Belästigung "und „Gefährdung "durch Lärm. *Zeitschrift für Lärmbekämpfung*, *33*, 2-7.
24. Krichagin, V. J. (1978). Health effects of noise exposure. *Journal of Sound and Vibration*, *59*(1), 65-71. doi: 10.1016/0022-460X(78)90479-0
25. Kryter, K. D. (1966). Psychological reactions to aircraft noise. *Science*, 151(716), 1346-1355. doi: 10.1126/science.151.3716.1346
26. Kryter, K. D. (1968). An example of" engineering psychology": The aircraft noise problem. *American Psychologist*, *23*(4), 240. doi: 10.1037/h0026248
27. Kumar, D. (2010). Noise pollution in India. *Journal of the Indian Medical Association*, 108(3), 139.
28. McLean, E. K., & Tarnopolsky, A. (1977). Noise, discomfort and mental health: a review of the socio-medical implications of disturbance by noise. *Psychological Medicine*, *7*(1), 19-62. doi: 10.1017/S0033291700023138
29. Meyer, A. (2003). Wenn kaum noch jemand weiter weiß. Eine repräsentative sozio-psychologische Fluglärmuntersuchung als Ausweg/ When no-one knows how to continue-A representative socio-psychological aircraft noise study as a solution. *Zeitschrift für Lärmbekämpfung*, *50*(5).
30. Moudon, A. V. (2009). Real noise from the urban environment: how ambient community noise affects health and what can be done about it. *American Journal of Preventive Medicine*, *37*(2), 167-171. doi: 10.1016/j.amepre.2009.03.019.
31. Murillo, I. C. (2007). How does noise affect us? In our health, life styles and environs. *Revista de enfermeria (Barcelona, Spain)*, *30*(10), 13-6.
32. Kryter, K. D. & Grandjean, E. (1960): *Mensch* & *Umwelt*. Die *Wirkungen des Lärms auf den Menschen.* Verlag: J. R. Geigy, Basel
33. Ortscheid, J., & Wende, H. (2000). Fluglärmwirkungen, Umweltbundesamt.
34. Pichot, P. (1992). Noise, sleep and behavior. *Bulletin de l'Academie Nationale de Médecine*, *176*(3),

393-9.

35. Pirrera, S., De Valck, E., & Cluydts, R. (2010). Nocturnal road traffic noise: A review on its assessment and consequences on sleep and health. *Environment International*, *36*(5), 492-498. doi: 10.1016/j.envint.2010.03.007
36. Röösli, M. (2013). Health effects of environmental noise exposure. *Therapeutische Umschau. Revue Therapeutique*, *70*(12), 720-724. doi: 10.1024/0040-5930/a000470
37. Schick, A., & Meis, M. (2001). Reducing individual noise-induced health risks with health psychology and stress coping. *Schriftenreihe des Vereins für Wasser-, Boden-und Lufthygiene*, (111), 106.
38. Schust, M. (2004). Effects of low frequency noise up to 100 Hz. *Noise and Health*, *6*(23), 73.
39. Shepherd, M. (1975). Pollution, noise, and mental health. *The Lancet*, *305*(7902), 322-324. doi: 10.1016/S0140-6736(75)91222-2
40. Spooner, H. J. (1922). Health problems involved in noise and fatigue. *Nation's Health*, *4*(91-95), 156-159
41. Spreng, M. (2004). Noise induced nocturnal cortisol secretion and tolerable overhead flights. *Noise and Health*, *6*(22), 35.
42. Stansfeld, S. A., Haines, M. M., Burr, M., Berry, B., & Lercher, P. (2000). A review of environmental noise and mental health. *Noise and Health*, *2*(8), 1.
43. Stansfeld, S. A. & Clark, C. (2008). Noise and psychiatric disorder. In: The impact of the Environment on Psychiatric Disorder (Freeman, H. & Stansfeld, S. eds), Routledge, Taylor & Francis Group. London & New York
44. Stansfeld, S., & Clark, C. (2015). Health effects of noise exposure in children. *Current Environmental Health Reports*, *2*(2), 171-178. doi: 10.1007/s40572-015-0044-1
45. Talbott, E., & Thompson, S. J. (1995). Health Effects from Environmental Noise. In: An Introduction to Environmental Epidemiology (Talbott, E. & Craun, G. F. eds.), Lewis Publishers. Boca Ranton, New York, London & Tokyo. 209.
46. Tarnopolski, A. (1979). The effects of aircraft noise. *Journal of Psychosomatic Research*, 23(6), 371-372.
47. Thompson, S. (1996). Non-auditory healty effects of noise: updated review. *Noise Control-The Next 25 Years, Proceedings of the Internoise'96, Institute of Acoustics*, 2177-2182.
48. Van Kamp, I., & Davies, H. (2008). Environmental noise and mental health: Five year review and future directions. In *Proceedings of the 9th International Congress on Noise as a Public Health Problem*.
49. van Kamp, I., van Kempen, E., Baliatsas, C., & Houthuijs, D. (2013). Mental health as context rather than health outcome of noise: competing hypotheses regarding the role of sensitivity, perceived soundscapes and restoration. In *Proceedings Internoise*.
50. Ward, W D. (1988). Jet noise and mental health. *British Journal of Audiology*, 22(2), 157-158.
51. Yano, T., Gjestland, T., & Lee, S. (2012). Community response to noise. *Noise and Health*, *14*(61), 303. doi: 10.4103/1463-1741.104898

Exposure (not noise, or noise not measured or modelled) (n = 40)

1. Abey-Wickrama, I., a'Brook, M. F., Gattoni, F. E. G., & Herridge, C. F. (1969). Mental-hospital admissions and aircraft noise. *The Lancet*, *294*(7633), 1275-1277. doi: 10.1016/S0140-6736(69)90810-1

2. Chen, H., Kwong, J. C., Copes, R., Tu, K., Villeneuve, P. J., Van Donkelaar, A., ... & Wilton, A. S. (2017). Living near major roads and the incidence of dementia, Parkinson's disease, and multiple sclerosis: a population-based cohort study. *The Lancet*, *389*(10070), 718-726. doi:10.1016/S0140-6736(16)32399-6
3. Devroey, D., Betz, W., & Coigniez, P. (2002). Influence of noise on the patients, health perception: An epidemiological registration. *Tijdschrift voor Geneeskunde*, *58*(21), 1392-7.
4. Dreger, S., Meyer, N., Fromme, H., & Bolte, G. (2015). Environmental noise and incident mental health problems: A prospective cohort study among school children in Germany. *Environmental Research*, *143*, 49-54. doi: 10.1016/j.envres.2015.08.003
5. Dzhambov, A. M., Markevych, I., Tilov, B., Arabadzhiev, Z., Stoyanov, D., Gatseva, P., & Dimitrova, D. D. (2018). Pathways linking residential noise and air pollution to mental ill-health in young adults. *Environmental Research*, *166*, 458-465. doi: 10.1016/j.envres.2018.06.031
6. Firdaus, G., & Ahmad, A. (2010). Noise pollution and human health: a case study of municipal corporation of Delhi. *Indoor and Built Environment*, *19*(6), 648-656. doi: 10.1177/1420326X10370532
7. Francois, J. (1981). Effect of aircraft noise on the equilibrium of airport residents: Longitudinal study around Roissy, phase 3.
8. Fyhri, A., & Klæboe, R. (2009). Road traffic noise, sensitivity, annoyance and self-reported health—A structural equation model exercise. *Environment International*, *35*(1), 91-97. doi: 10.1016/j.envint.2008.08.006
9. Gattoni, F., & Tarnopolsky, A. (1973). Aircraft noise and psychiatric morbidity. *Psychological Medicine*, *3*(4), 516-520. doi: 10.1017/S0033291700054337
10. Gielkens C, Roberts A. Geluidshinder en de (ervaren) gezondheid: het vervolg. geluid. 2003;1:9–12.
11. Hand, D. J., Tarnopolsky, A., Barker, S. A., & Jenkins, L. M. (1980). Relationships between psychiatric hospital admissions and aircraft noise: A new study. *Proc. Int. Congr. Noise as a Public Health Problem, ASHA Rep*, *10*, 277-282.
12. Hardoy, M. C., Carta, M. G., Marci, A. R., Carbone, F., Cadeddu, M., Kovess, V., ... & Carpiniello, B. (2005). Exposure to aircraft noise and risk of psychiatric disorders: the Elmas survey. *Social Psychiatry and Psychiatric Epidemiology*, *40*(1), 24-26. doi:10.1007/s00127-005-0837-x
13. Hatfield, J., Job, R. F. S., Carter, N. L., Peploe, P., Taylor, R., & Morrell, S. (2001). The influence of psychological factors on self-reported physiological effects of noise. *Noise and Health*, *3*(10), 1.
14. Hatfield, J., Job, R. S., Hede, A. J., Carter, N. L., Peploe, P., Taylor, R., & Morrell, S. (2002). Human response to environmental noise: the role of perceived control. *International Journal of Behavioral Medicine*, *9*(4), 341-359. doi: 10.1207/S15327558IJBM0904_04
15. Hattori, M. (2000). A field study of health effects of aircraft noise in adults around Komatsu Air Base (1998). *[Nihon koshu eisei zasshi] Japanese Journal of Public Health*, *47*(1), 20-31.
16. Herridge, C. F. (1972). Aircraft noise and mental hospital admission. *British Journal of Audiology*, *6*(2), 32-36. doi: 10.3109/00381797209075555
17. Jenkins, L. M., Tarnopolsky, A., Hand, D. J., & Barker, S. M. (1979). Comparison of three studies of aircraft noise and psychiatric hospital admissions conducted in the same area. *Psychological Medicine*, *9*(4), 681-693. doi: 10.1017/S0033291700033997

18. Jenkins, L., Tarnopolsky, A., & Hand, D. (1981). Psychiatric admissions and aircraft noise from London Airport: four-year, three-hospitals' study. *Psychological Medicine*, *11*(4), 765-782. doi: 10.1017/S0033291700041271
19. Jensen, H. A., Rasmussen, B., & Ekholm, O. (2018). Neighbour and traffic noise annoyance: a nationwide study of associated mental health and perceived stress. *European Journal of Public Health*, *28*(6), 1050-1055. doi: 10.1093/eurpub/cky091.
20. Kishikawa, H., Matsui, T., Uchiyama, I., Miyakawa, M., Hiramatsu, K., & Stansfeld, S. A. (2009). Noise sensitivity and subjective health: Questionnaire study conducted along trunk roads in Kusatsu, Japan. *Noise and Health*, *11*(43), 111. doi: 10.4103/1463-1741.50696
21. Knipschild, P. (1977). V. Medical effects of aircraft noise: community cardiovascular survey. *International Archives of Occupational and Environmental Health*, *40*(3), 185-190. doi: 10.1007/BF01842081
22. Knipschild, P. (1977). VI. Medical effects of aircraft noise: general practice survey. *International Archives of Occupational and Environmental Health*, *40*(3), 191-196. doi: 10.1007/BF01842082
23. Knipschild, P., & Oudshoorn, N. (1977). VII. Medical effects of aircraft noise: drug survey. *International Archives of Occupational and Environmental Health*, *40*(3), 197-200. doi: 10.1007/BF01842083
24. Koszarny, Z., Szata, W., & Goryński, P. (1982). Comparative evaluation of indicators of street noise with reference to the effect on the Warsaw's population. *Roczniki Panstwowego Zakladu Higieny*, *33*(5-6), 469-481.
25. Koszarny, Z. (2000). The effect of intensive traffic noise on well-being and self-assessed health status of urban population. *Roczniki Panstwowego Zakladu Higieny*, *51*(2), 191-201.
26. Kryter, K. D. (1990). Aircraft noise and social factors in psychiatric hospital admission rates: a re-examination of some data. *Psychological Medicine*, *20*(2), 395-411. doi: 10.1017/S0033291700017712
27. Kryter, K. D. (1990). "Aircraft noise and social factors in psychiatric hospital admission rates: A re-examination of some data": Erratum.
28. Lee, K. W., Kim, S. J., Park, J. B., Min, K. B., Kil, H. G., Lee, C., & Lee, K. J. (2011). Relationship Between Aircraft Noise Exposure and Depression, Anxiety, Stress in Korea. *Epidemiology*, *22*(1), S258.
29. Miyakawa, M., Matsui, T., Uchiyama, I., Hiramatsu, K., Hayashi, N., Morita, I., ... & Ohashi, S. (2008). Relationship between subjective health and disturbances of daily life due to aircraft noise exposure—Questionnaire study conducted around Narita International Airport—. In *Proc. 9th International conference on Noise as a Public Health Problem* (pp. 314-321).
30. Niemann, H., Maschke, C., & Hecht, K. (2005). Noise induced annoyance and morbidity. Results from the pan European LARES-survey. *Bundesgesundheitsblatt, Gesundheitsforschung, Gesundheitsschutz*, *48*(3), 315-328. doi: 10.4103/1463-1741.33537
31. Niemann, H., Bonnefoy, X., Braubach, M., Hecht, K., Maschke, C., Rodrigues, C., & Robbel, N. (2006). Noise-induced annoyance and morbidity results from the pan-European LARES study. *Noise and Health*, *8*(31), 63. doi: 10.4103/1463-1741.33537
32. Pisani, S., Bonarrigo, D., Gambino, M., Macchi, L., Banfi, F., Verri, A. M., ... & Cortinovis, I. (2003). Salus domestica epidemiological study: evaluation of health harm in a sample of women living close to Malpensa 2000 Airport. *EPIDEMIOLOGIA E PREVENZIONE*, (4),

234-241.

33. Putrik, P., de Vries, N. K., Mujakovic, S., van Amelsvoort, L., Kant, I., Kunst, A. E., ... & Jansen, M. (2015). Living environment matters: relationships between neighborhood characteristics and health of the residents in a Dutch municipality. *Journal of Community Health*, *40*(1), 47-56. doi: 10.1007/s10900-014-9894-y
34. Ristovska, G., Gjorgjev, D., & Jordanova, N. P. (2004). Psychosocial effects of community noise: cross sectional study of school children in urban center of Skopje, Macedonia. *Croatian Medical Journal*, *45*(4), 473-476.
35. Tarnopolsky, A., Barker, S. M., Wiggins, R. D., & McLean, E. K. (1978). The effect of aircraft noise on the mental health of a community sample: a pilot study. *Psychological Medicine*, *8*(2), 219-233. doi: 10.1017/S0033291700014276
36. Tarnopolsky, A., Watkins, G., & Hand, D. J. (1980). Aircraft noise and mental health: I. Prevalence of individual symptoms. *Psychological Medicine*, *10*(4), 683-698. doi: 10.1017/S0033291700054982
37. Tarnopolsky, A., Morton-Williams, J., & Barker, S. M. (1980). *Aircraft noise and prevalence of psychiatric disorders*. Social & Community Planning Research.
38. Turnovska, T., Staykova, J., & Petkov, T. (2004). Health assessment of populations living close to the airport of Bourgas, Bulgaria. *Arhiv za Higijenu rada i Toksikologiju*, *55*(1), 5-10.
39. Watkins, G., Tarnopolsky, A., & Jenkins, L. M. (1981). Aircraft noise and mental health: II. Use of medicines and health care services. *Psychological Medicine*, *11*(1), 155-168 doi: 10.1017/S003329170005337X.
40. Weigl, K., Herr, C. E., Meyer, N., Otto, C., Stilianakis, N., Bolte, G., ... & Kolb, S. (2018). Predictors of Health-related Quality of Life in Bavarian Preschool Children. *Gesundheitswesen, 80* (S 01): S1-S4. doi: 10.1055/s-0042-104117

Unrelated research topic (n = 12)

1. Agaeva, M. (2010). Precedence effect for moving sound. *International Journal of Psychophysiology*, *77*(3), 302. doi: 10.1016/j.ijpsycho.2010.06.195
2. Baliatsas, C., van Kamp, I., Swart, W., Hooiveld, M., & Yzermans, J. (2016). Noise sensitivity: Symptoms, health status, illness behavior and co-occurring environmental sensitivities. *Environmental Research*, *150*, 8-13. doi: 10.1016/j.envres.2016.05.029
3. Cantrell, R. W. (1974). Prolonged exposure to intermittent noise: audiometric, biochemical, motor, psychological and sleep effects. *The Laryngoscope*, *84* (10 Pt 2 Suppl 1), 1.
4. Favre, B. (1985). Relationship between traffic noise and effect in people; development of an exposure model. *Rev Acoust.* 18(74), 409-421.
5. Favre, B. (1985). Relationship between traffic noise and effect in people; application to a typical urban environment. *Rev Acoust.* 18(74), 423-438.
6. Hayashi, C., Hayashi, F., Kodama, H., & Kondo, S. (1973). Construction of PAANI (Psychological Assessment of Aircraft Noise Index) using statistical and psychological ideas. *Proceedings of the Institute of Statistical Mathematics, 1*(21).
7. Kryter, K. D. (2007). Acoustical, sensory, and psychological research data and procedures for their use in predicting effects of environmental noises. *The Journal of the Acoustical Society of America*, *122*(5), 2601-2614. doi: 10.1121/1.2782748
8. Lercher, P., Schmitzberger, R., & Kofler, W. (1995). Perceived traffic air pollution, associated

behavior and health in an alpine area. *Science of the Total Environment*, *169*(1-3), 71-74. doi: 10.1016/0048-9697(95)04634-D

9. Stansfeld, S. A., Clark, C. R., Jenkins, L. M., & Tarnopolsky, A. (1985). Sensitivity to noise in a community sample: I. Measurement of psychiatric disorder and personality. *Psychological Medicine*, *15*(2), 243-254. doi: 10.1017/S0033291700023527
10. Stansfeld, S. A., Gallacher, J. E. J., Sharp, D. S., & Yarnell, J. W. G. (1991). Social factors and minor psychiatric disorder in middle-aged men: a validation study and a population survey. *Psychological Medicine*, *21*(1), 157-167. doi: 10.1017/S0033291700014744
11. Stansfeld, S. A. (1992). Noise, noise sensitivity and psychiatric disorder: epidemiological and psychophysiological studies. *Psychological Medicine Monograph Supplement*, *22*, 1-44. doi: 10.1017/S0264180100001119
12. Wunderli, J. M., Pieren, R., Habermacher, M., Vienneau, D., Cajochen, C., Probst-Hensch, N., ... & Brink, M. (2016). Intermittency ratio: A metric reflecting short-term temporal variations of transportation noise exposure. *Journal of Exposure Science and Environmental Epidemiology*, *26*(6), 575. doi:10.1038/jes.2015.5

Full-Text could not be obtained (n = 9)

1. Albertini, C. (1931). La lotta contro i rumori ei rumori della strada. Vita Italiana
2. Guthof, O., & Gableske, R. (1968). Effects of traffic noise on human life. *Das Öffentliche Gesundheitswesen*, *30*(1), 1-6.
3. Haider, M. (1976). Air pollution, noise-current environmental problems. *Wiener Medizinische Wochenschrift (1946)*, *126*(6), 65.
4. Ising, H. & Rebentisch, E. (1992). Environmental noise: Exposure, annoyance and long-term effects on health. [German]. *Wissenschaft und Umwelt*, (2), 147-150.
5. Kelly, A. (1986). Effects of aircraft noise and noise sensitivity on the physical and mental well-being of older adults. Keine weiteren Angaben vorhanden.
6. Lohman, A. (2007). The impact of a freeway on neighborhood: Sense of community, size, and methods of measurement. Dissertation. The Claremont Graduate University. AAI3233758.
7. Schick, A. (1992): The psychological aspects of noise research. *Zeitschrift für Lärmbekämpfung*, 39 (4) 113-117.
8. Vartiainen, A.-K., Turunen, A. W., Ung-Lanki, S. & Lanki, T. (2015). Meluherkkyydellä on tärkeä rooli melun kokemisessa. *Psykologia, 50(4)*, 244-256.
9. Yamamoto, K. (1993). Living environment stress and mental health--From the ten years' study of psychosocial stress in inhabitants along the loop road 7 in Metropolitan Tokyo. *Journal of Mental Health*, 39, 41-54.

Screening instrument was not validated (n = 6)

1. Belojević, G., Jakovljević, B., & Aleksić, O. (1997). Subjective reactions to traffic noise with regard to some personality traits. *Environment International*, *23*(2), 221-226. doi: 10.1016/S0160-4120(97)00008-1
2. Öhrström, E. (1989). Sleep disturbance, psycho-social and medical symptoms—a pilot survey among persons exposed to high levels of road traffic noise. *Journal of Sound and Vibration*, *133*(1), 117- doi: 10.1016/0022-460X(89)90986-3
3. Öhrström, E. (1991). Psycho-social effects of traffic noise exposure. *Journal of Sound and Vibration*, *151*(3), 513-517. doi: 10.1016/0022-460X(91)90551-T

4. Öhrström, E., & Skånberg, A. (2000). Adverse health effects in relation to noise mitigation—A longitudinal study in the city of Göteborg. In *Proceedings of the 29th International Congress and Exhibition on Noise Control Engineering, Nice, France* (pp. 27-30).
5. Öhrström, E. (2004). Longitudinal surveys on effects of changes in road traffic noise—annoyance, activity disturbances, and psycho-social well-being. *The Journal of the Acoustical Society of America*, *115*(2), 719-729. doi: 10.1121/1.1639333
6. Yoshida, T., Osada, Y., Kawaguchi, T., Hoshiyama, Y., Yoshida, K., & Yamamoto, K. (1997). Effects of road traffic noise on inhabitants of Tokyo. *Journal of Sound and Vibration*, *205*(4), 517-522. doi: 10.1006/jsvi.1997.1020

Industry-/ Military Aircraft Noise (n = 4)

1. Hiramatsu, K., Yamamoto, T., Taira, K., Ito, A., & Nakasone, T. (1997). A survey on health effects due to aircraft noise on residents living around Kadena air base in the Ryukyus. *Journal of Sound and Vibration*, *205*(4), 451-460. doi: 10.1006/jsvi.1997.1011
2. Hiramatsu, K., Matsui, T., Miyakita, T., Ito, A., Tokuyama, T., Osada, Y., & Yamamoto, T. (2002). Population-based questionnaire survey on health effects of aircraft noise on residents living around US airfields in the Ryukyus—Part II: An analysis of the discriminant score and the factor score. *Journal of Sound and Vibration*, *250*(1), 139-144. doi: 10.1006/jsvi.2001.3896
3. Matsui, T. (2013, September). Psychosomatic disorder due to aircraft noise and its causal pathway. In *INTER-NOISE and NOISE-CON Congress and Conference Proceedings*, *247*(8), 605-609. Institute of Noise Control Engineering.
4. Stošić, L., & Blagojević, L. (2011). Environmental noise and mental disturbances in urban population. *Acta Medica Medianae*, *50*(3), 34-39. doi: 10.5633/amm.2011.0306

Population of employees considered (not general population) (n = 3)

1. Akan, Z., Yilmaz, A., Özdemir, O., & Korpinar, M. A. (2012). Noise pollution, psychiatric symptoms and quality of life: noise problem in the east region of Turkey. *Journal of Inonu University Medical Faculty*, *19*(2), 75-81. doi: 10.7247/jiumf.19.2.3
2. Calapaj, G. G., & Bellia, G. (1969). Psychometric studies of subjects exposed to the noise of jet engines. *La Medicina del Lavoro*, *60*(1), 43.
3. Chiovenda, P., Pasqualetti, P., Zappasodi, F., Ercolani, M., Milazzo, D., Tomei, G., ... & Tecchio, F. (2007). Environmental noise-exposed workers: Event-related potentials, neuropsychological and mood assessment. *International Journal of Psychophysiology*, *65*(3), 228-237. doi:10.1016/j.ijpsycho.2007.04.009

Experimental study design (n = 3)

1. Naqvi, F., Haider, S., Perveen, T., & Haleem, D. J. (2012). Sub-chronic exposure to noise affects locomotor activity and produces anxiogenic and depressive like behavior in rats. *Pharmacological Reports*, *64*(1), 64-69. doi: 10.1016/S1734-1140(12)70731-4
2. Granati, *A.*, Angeleri, F., Lenzi, R. (1959). *L'*influenza dei rumori sul sistema nervoso – Folia Medica: XLII (11): 1314-1325.
3. Schönpflug, W., Kausche, J. & Wieland, R. (1978). Traffic noise during leisure time. [German]. *Kampf dem Lärm*, 25(1): 21-25.

Results not usable (see reference for more details) (n = 3)

1. Bodin, T., Albin, M., & Bjork, J. (2013). Road traffic noise and mental health-Preliminary results from a cross-sectional study in southern Sweden. In *INTER-NOISE and NOISE-CON Congress*

and Conference Proceedings, 247(2), 5768-5772. Institute of Noise Control Engineering.

Only descriptive results depicted without numbers;

Author did not respond to requests for more information

2. Greiser, E., Greiser, C., & Janhsen, K. (2007). Night-time aircraft noise increases prevalence of prescriptions of antihypertensive and cardiovascular drugs irrespective of social class—the Cologne-Bonn Airport study. *Journal of Public Health*, *15*(5), 327-337. doi: 10.1007/s10389-007-0137-x

Anxiolytics were evaluated together with blood pressure and heart medications

3. Houthujis, D. J. M. & van Wiechen, C. M. A. G. (2006). Monitoring van gezondheid en beleving rondom de luchthaven Schiphol. RIVM rapport 630100003/2006

Author did not respond to requests for more information

Convenience Sample (n = 2)

1. Dzhambov, A., Tilov, B., Markevych, I., & Dimitrova, D. (2017). Residential road traffic noise and general mental health in youth: the role of noise annoyance, neighborhood restorative quality, physical activity, and social cohesion as potential mediators. *Environment International*, *109*, 1-9. doi: 10.1016/j.envint.2017.09.009.
2. Maschke, C., & Hecht, K. (2005). Tag-Nacht Unterschiede in der multifaktoriellen Genese von lärminduzierten Erkrankungen–Ergebnisse einer epidemiologischen Studie. *Somnologie*, *9*(2), 96-104. doi: 10.1111/j.1439-054X.2005.00046.x

Repeat publication (n = 1)

1. Greiser, E., & Glaeske, G. (2013). Social and economic consequences of night-time aircraft noise in the vicinity of Frankfurt/Main airport. *Gesundheitswesen (Bundesverband der Ärzte des Öffentlichen Gesundheitsdienstes (Germany))*, *75*(3), 127-133.

Outcome not differentiated (n = 1)

1. Wright, D. M., Newell, K., Maguire, A., & O'Reilly, D. (2018). Aircraft noise and self-assessed mental health around a regional urban airport: a population based record linkage study. *Environmental Health*, *17*(1), 74. doi: 10.1186/s12940-018-0418-6.

3. Leave-one-out Analysis

Table 3. Leave-one-out analysis for depression and exposure to road traffic noise.

Study Omitted	ES	95% CI	
Floud 2011	1.03	0.99	1.07
Halonen 2013	1.03	0.99	1.07
Klompmaker 2019	1.04	1.00	1.07
Leijssen 2019	1.03	0.99	1.07
Okokon	1.02	0.99	1.06
Orban 2016	1.02	0.99	1.05
Seidler 2017	1.02	0.98	1.07
Stansfeld 1993, 1996	1.02	0.99	1.06
Sygna 2014	1.03	0.99	1.06
Tzivian 2018	1.02	0.99	1.06
Zock 2018	1.03	0.99	1.06

ES Effect estimate, CI confidence interval.

Table 4. Leave-one-out analysis for depression and exposure to aircraft noise.

Study Omitted	ES	95% CI	
Baudin 2018	1.14	1.12	1.15
Floud 2011	1.14	1.12	1.16
Schreckenberg 2009	1.14	1.12	1.15
Seidler 2017	1.06	0.95	1.19
Van Kamp 2007	1.14	1.12	1.15

ES Effect estimate, CI confidence interval.

Table 5. Leave-one-out analysis for anxiety and exposure to road traffic noise.

Study Omitted	ES	95% CI	
Bocquier 2014	1.07	1.01	1.13
Floud 2011	1.02	0.98	1.07
Halonen 2013	1.03	0.99	1.07
Klompaker 2019	1.00	0.98	1.02
Okokon 2018	1.02	0.98	1.06
Zock 2018	1.02	0.98	1.07

ES Effect estimate, CI confidence interval.

4. Publication Bias

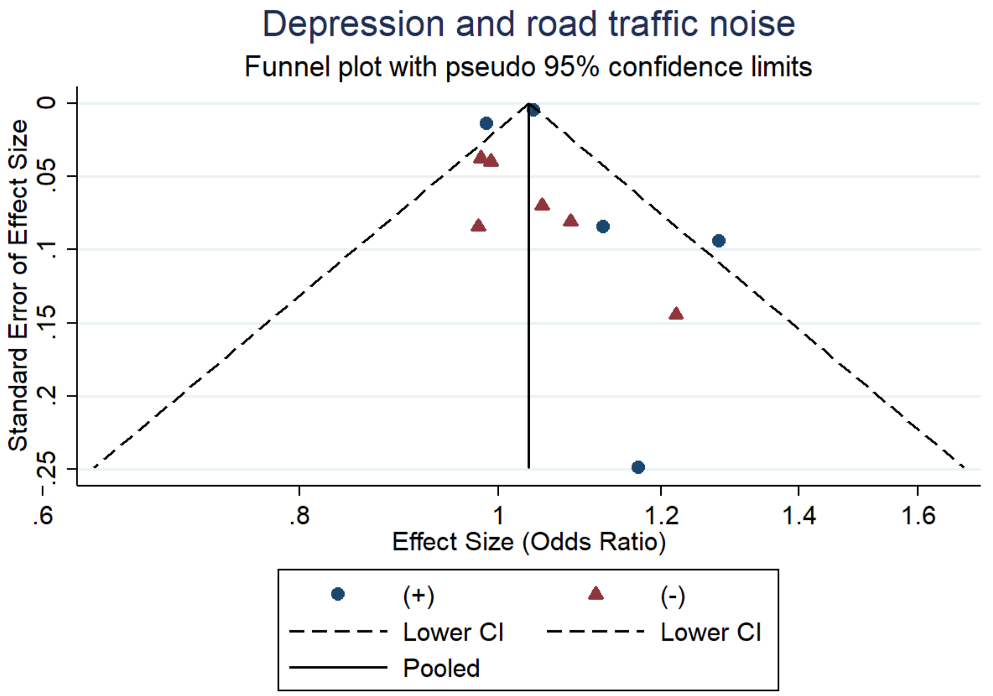

Figure 1. Funnel plot for depression (based on anti-depressant use, depressive episodes diagnoses, detected with validated a screening instrument) and road traffic noise.

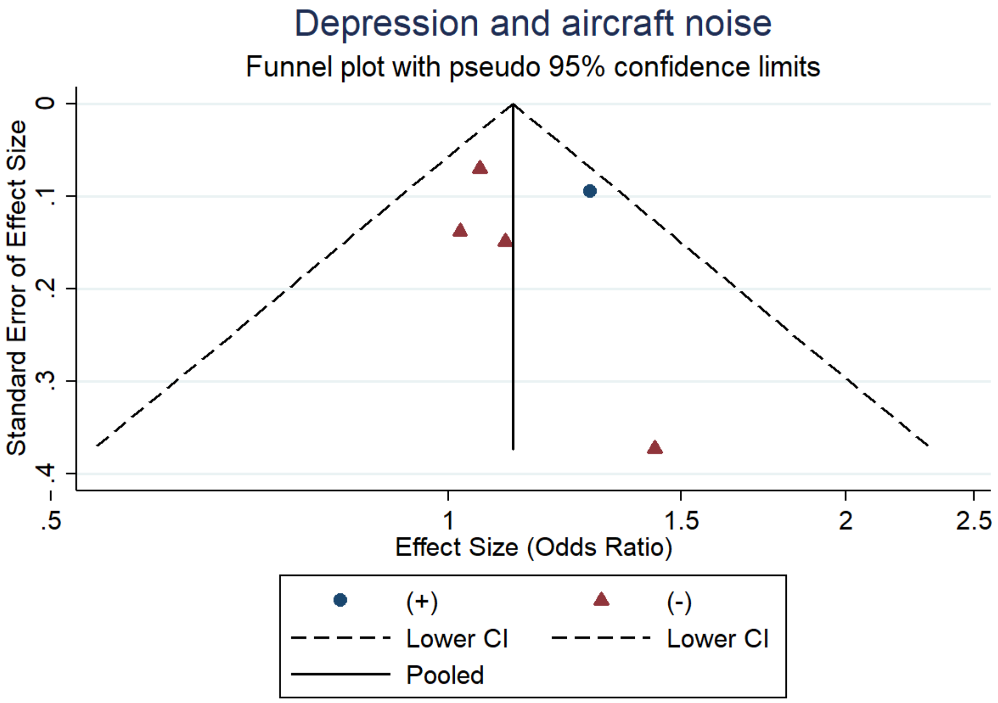

Figure 2. Funnel plot for depression (based on anti-depressant use, depressive episodes diagnoses, detected with validated a screening instrument) and aircraft noise.

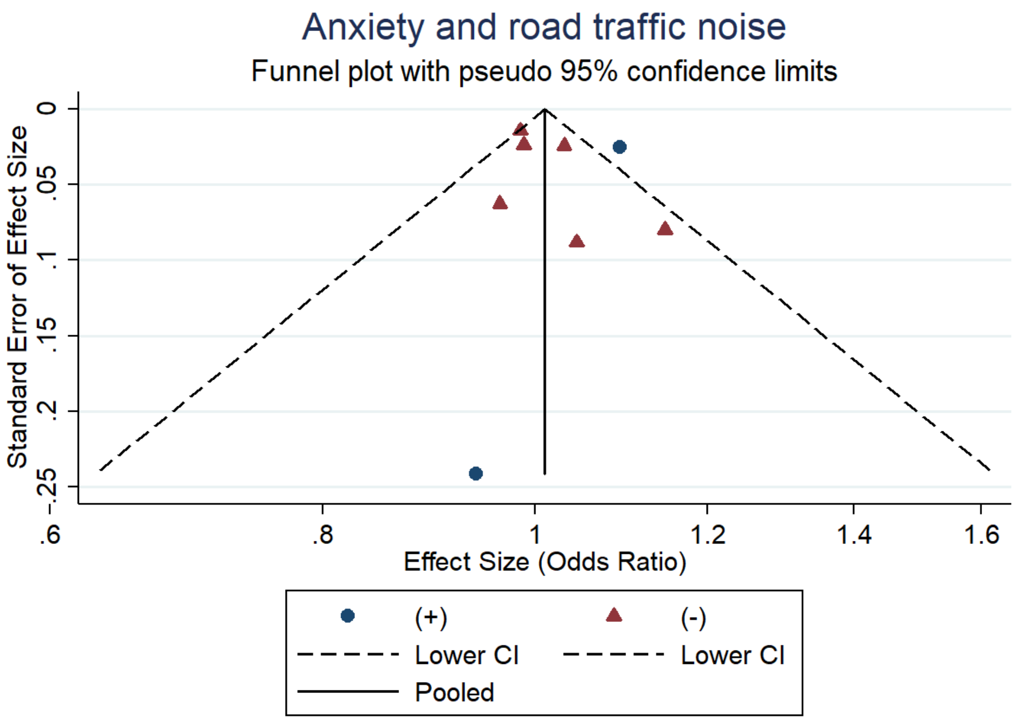

Figure 3. Funnel plot for anxiety (based on anxiolytics use, diagnoses, detected with validated a screening instrument) and road traffic noise.
